# Guest-Induced Vibration Locking and Fluorescence Switching in a Pyrene-Functionalized Covalent Organic Framework (COF) for Perfluorooctanoic Acid (PFOA) Recognition

**DOI:** 10.1021/jacs.6c01527

**Published:** 2026-08-28

**Authors:** Gobinda Das, Thirumurugan Prakasam, Nour Alkhatib, Sudhir Kumar Sharma, Hoda Al-assaad, Farah Benyettou, Rasha G. AbdulHalim, Hamzeh Sabouni, Kareem Mazen, Ramesh Jagannathan, Charles J.-A. Jackson, Felipe Gándara, Andreas Mavrandonakis, Mark A. Olson, Samer Aouad, Mohamad El-Roz, Serdal Kirmizialtin, Sabu Varghese, Ali Trabolsi

**Affiliations:** † Science Division, New York University Abu Dhabi, Saadiyat Island, P.O. Box 129188, Abu Dhabi, United Arab Emirates; ‡ Engineering Division, New York University Abu Dhabi, Saadiyat Island, P.O. Box 129188, Abu Dhabi, United Arab Emirates; § ENSICAEN, UNICAEN, CNRS, Laboratoire Catalyse et Spectrochimie, Normandie Université, 14000 Caen, France; ∥ Materials Science and Engineering Program, College of Arts and Sciences, 47767 American University of Sharjah, POB 26666, Sharjah, United Arab Emirates; ⊥ Department of Physical and Environmental Sciences, 14738 Texas A&M University Corpus Christi, 6300 Ocean Dr., Corpus Christi, Texas 78412, United States; # Instituto de Ciencia de Materiales de Madrid-CSIC, C. Sor Juana Inés de la Cruz 3, 28049, Madrid, Spain; ∇ Department of Chemistry,University of Balamand, P.O. Box, 100 Lebanon, Lebanon; ○ 535277 CTP, New York University Abu Dhabi, Saadiyat Island, P.O. Box 129188, Abu Dhabi, United Arab Emirates; ◆ Water Research Center, New York University Abu Dhabi, Saadiyat Island, P.O. Box 129188, Abu Dhabi, United Arab Emirates

## Abstract

Light-up luminescent covalent organic framework (COF) sensors hold promise for rapid and selective detection of molecular pollutants, yet their performance is often limited by insufficient guest-induced rigidity and incomplete mechanistic understanding. Here, we introduce a pyrene-functionalized flexible covalent organic framework (PY-TTA COF) that undergoes a guest-induced molecular-locking process upon exposure to perfluorooctanoic acid (PFOA), producing a pronounced fluorescence turn-on response in aqueous media. The pendent pyrene chromophores, which project into the pore interior, create an adaptable microenvironment in which PFOA binding, involving hydrogen-bonding interactions, possible proton-transfer processes at imine/pyridine nitrogen sites, and C–F···π interactions, restricts local molecular motions and enhances radiative emission. Solid-state NMR spectroscopy, supported by semiempirical calculations, provides molecular-level insight into the hydrogen-bonding and fluorine–aromatic proximities associated with this locking effect, while PXRD and TEM reveal guest-induced structural modulation while confirming preservation of framework crystallinity. This guest-locked luminescence enhancement enables fluorescence-based and recyclable detection of PFOA with a limit of detection of 2.7 μg L^–1^. The demonstrated mechanism establishes a design strategy for developing stimuli-responsive COFs with tunable photophysical properties and environmentally relevant sensing capabilities.

## Graphical abstract

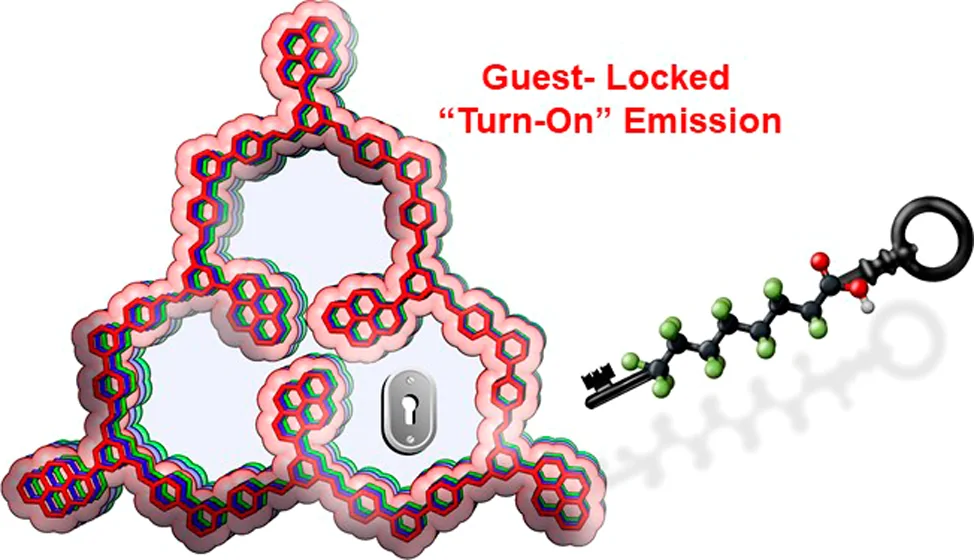

## Introduction

Light-up luminescent sensing,^1^ in which guest binding triggers a pronounced emission enhancement, offers an elegant strategy for achieving high sensitivity and selectivity. Among the various signal-amplification mechanisms, restriction of host molecular vibrations at room temperature has recently emerged as a powerful strategy for suppressing nonradiative decay pathways and enhancing radiative emission.^1–5^ In this process, the binding of a guest sterically or electronically restricts the dynamic motions of fluorophore units that would otherwise dissipate excitation energy nonradiatively, resulting in an immediate fluorescence “turn-on” response.^6–8^ This behavior resembles a molecular lock-and-key event, wherein only a complementary guest can effectively restrict the host’s vibrational degrees of freedom.^9^ Such vibration-locking provides two major advantages: (i) intrinsic fluorescence amplification, in which even weak host–guest interactions can produce large emission increases,^10^ and (ii) the potential to achieve molecular selectivity, enabling discrimination among closely related analytes.^11^

The ability to modulate molecular vibrations within ordered porous materials^12^ has therefore become a compelling strategy for tuning photophysical properties relevant for chemical sensing,^13^ gas storage,^14–16^ and separations.^17^ To date, most demonstrations of guest-induced motion locking have been realized in systems in which specific nodes, linkers, or supramolecular contacts permit local conformational freedom,^18–20^ such as metal–organic frameworks (MOFs),^9,21–23^ hydrogen-bonded frameworks (HOFs),^18^ and supramolecular organic frameworks (SOFs)^24^ due to their inherent flexibility. In contrast, analogous examples in covalent organic frameworks (COFs), where dynamic molecular motions can be coupled to guest recognition and optical response, remain comparatively underexplored.

COFs are crystalline porous materials constructed from organic linkers connected through robust covalent bonds.^25,26^ Like MOFs, SOFs, and HOFs, COFs offer precise and predictable control over pore size, shape, and chemical functionality, making them attractive platforms for the development of responsive materials.^27–29^ Their ordered pores also provide an opportunity to introduce freely mobile functional units whose motion can become restricted upon engagement with complementary guests.^28^ This architectural feature enables lock-and-key-type molecular recognition processes in which suitable guests restrict the vibrational freedom of these dangling units, offering a route to modulate emission through controlled host–guest interactions.^11,27,30–32^ Despite these advantages, examples in which guest-induced restriction of local molecular motion is deliberately engineered to generate fluorescence turn-on responses in COFs remain scarce.^7,8,33,34^ Developing such systems would provide a means to directly couple molecular recognition with photophysical amplification in a crystalline porous framework.

We recently reported a guanidinium-based COF that becomes emissive at cryogenic temperatures due to the suppression of intramolecular motion under these conditions.^6^ Although this observation provides valuable mechanistic insight, its practical utility is limited by the requirement of extremely low temperatures. Achieving vibration locking at room temperature would significantly broaden the applicability and technological relevance of this sensing concept.

At the same time, the detection of per- and polyfluoroalkyl substances (PFAS), particularly perfluorooctanoic acid (PFOA), remains an important challenge because of their environmental persistence, bioaccumulation, and widespread occurrence in contaminated water systems.^35–37^ Accordingly, designing porous materials capable of translating PFAS recognition into a measurable optical response represents a timely and important objective.

Herein, we report a pyrene-functionalized covalent triazine framework (PY-TTA COF, Figure 1a) synthesized by microwave-assisted Schiff-base condensation. Unlike conventional pyrene-based 2D COFs,^38–43^ where pyrene units are rigidly embedded in the framework backbone, the PY-TTA COF contains dangling pyrene chromophores that project toward the pore interior. Pyrene was selected because its strong fluorescence, extended aromatic surface, and ability to undergo local rotational and vibrational motion when incorporated as a pendant group make it particularly well suited for probing guest-induced rigidification within the pore environment. In the guest-free state, these moieties retain substantial rotational freedom, promoting nonradiative vibrational relaxation, which results in weak intrinsic emission. Upon binding guests of complementary size and shape, a shape-recognition-driven locking process occurs: the pore acts as the “lock,” the guest as the “key,” and the interaction selectively restricts the motion of the pyrene units. This targeted molecular recognition event produces a pronounced fluorescence turn-on response toward PFOA at room temperature.

**Figure 1:**
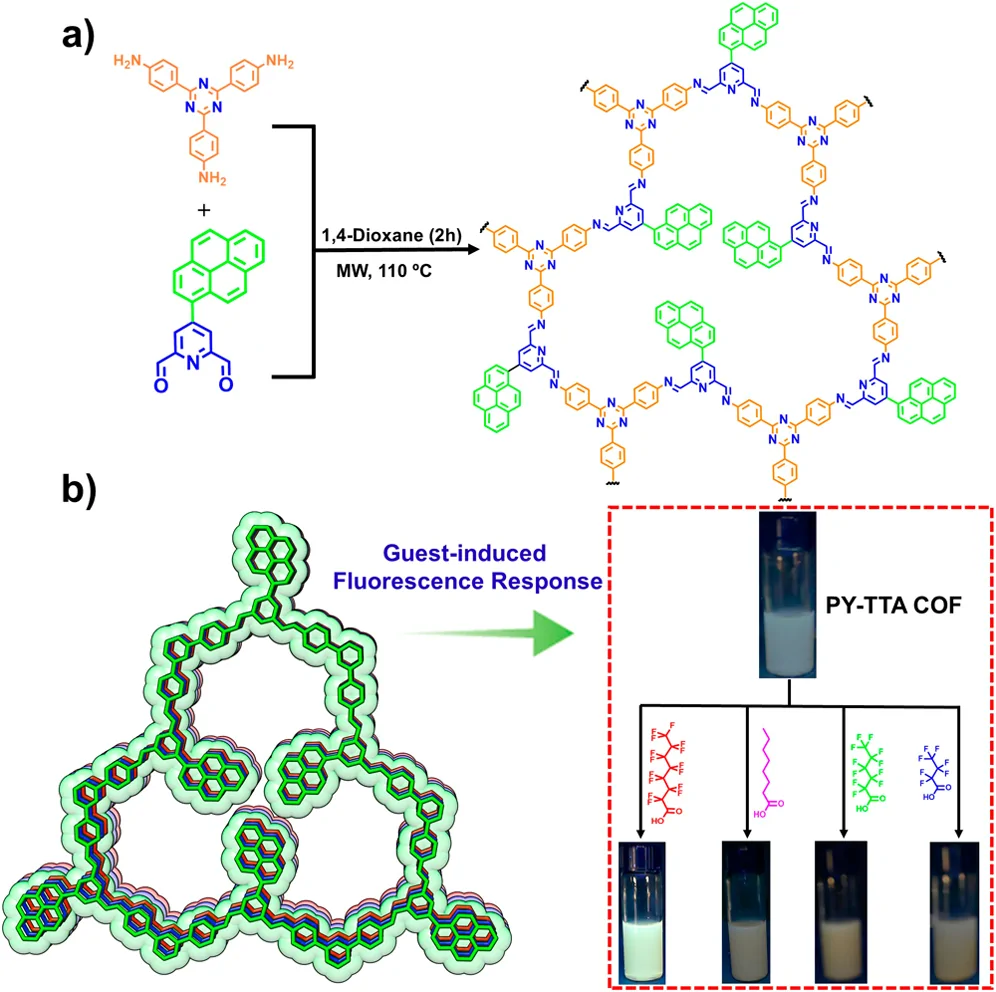
(a) Synthesis and chemical structure of PY-TTA COF prepared via imine condensation of 4,4′,4′′-(1,3,5-triazine-2,4,6-triyl)­trianiline (TTA, orange) and 4-(pyren-2-yl)­pyridine-2,6-dicarbaldehyde (PY, blue) in 1,4-dioxane under microwave (MW) irradiation at 110 °C for 2 h. (b) Schematic illustration of the PY-TTA COF pore architecture, highlighting the pendent pyrene chromophores projecting into the pore interior and the corresponding fluorescence response of the COF suspension toward PFOA and representative control analytes, including octanoic acid (OA), perfluorohexanoic acid (PFHA), and perfluorobutanoic acid (PFBA).

Through a combination of solid-state NMR spectroscopy, adsorption studies, structural characterization, and semiempirical calculations, we elucidate the molecular-level interactions responsible for the guest-locking process and demonstrate how controlled local dynamics within a flexible COF can be harnessed for PFAS recognition and fluorescence sensing.

## Results and Discussion

The PY-TTA COF was synthesized via an imine condensation reaction between 4-(pyren-2-yl)­pyridine-2,6-dicarbaldehyde (PY) and 4,4′,4′′-(1,3,5-triazine-2,4,6-triyl) trianiline (TTA) under microwave irradiation at 110 °C for 2 h (see Supporting Information for details). The reaction afforded a yellow powder in 54% isolated yield. Formation of the extended COF network was confirmed by Fourier transform infrared (FT-IR, Figure S1) spectroscopy and solid-state ^13^C cross-polarization/magic angle spinning nuclear magnetic resonance spectroscopy (CP/MAS NMR, Figure S2). FT-IR spectra showed the disappearance of the aldehyde C=O stretching band at 1701 cm^–1^, indicating the complete consumption of the monomers and successful formation of the imine-linked framework.

The ^13^C CP/MAS NMR spectrum of PY-TTA COF provides clear evidence for imine bond formation, showing a characteristic C=N resonance at ∼147 ppm together with the expected triazine carbon resonance (∼169.3 ppm) and no detectable residual carbonyl signal from the PY precursor (Figure S2). Powder X-ray diffraction (PXRD) analysis confirmed the crystalline nature of the framework. The PXRD pattern (Figure S3) shows a prominent reflection at 2θ = 5.7°, indexed as the (110) plane, along with weaker reflections at 2θ = 10.3°, 12°, and 14.8°, corresponding to the (42̅0), (400), and (51̅0) planes, respectively. A broad reflection at 2θ = 26.3° is assigned to the (001) reflection, indicative of interlayer stacking. Based on the anticipated connectivity of the monomers, a distorted honeycomb structure with an ABC eclipsed stacking arrangement^44^ (Figure 1b) was constructed using Materials Studio. The simulated PXRD pattern based on this structural model reproduces the positions of the principal diffraction features observed experimentally, providing support for the proposed ABC-eclipsed stacking arrangement of PY-TTA COF.

Nitrogen adsorption–desorption measurements at 77 K exhibit a Type I isotherm with pronounced uptake at low relative pressures (Figure S4), consistent with the microporous nature of the framework. The Brunauer–Emmett–Teller (BET) surface area of the PY-TTA COF was calculated to be 373 m^2^/g. Nonlocal DFT (NLDFT) analysis of the pore size distribution yielded a dominant pore diameter of 1.8 nm (Figure S5), consistent with the value predicted from structural modeling.

The morphology of PY-TTA COF was investigated by scanning electron microscopy (SEM, Figure 2a and Figure S6), high-resolution transmission electron microscopy (HRTEM, Figure 2b-f and Figure S7), and tapping mode atomic force microscopy (AFM, Figure 2g, 2h, and Figure S8). SEM images show aggregates of spherical particles with a rough surface texture. HRTEM images show that the particles have an average diameter of approximately 240 nm and exhibit a spiked surface texture. AFM measurements revealed particle heights of approximately 10 nm, reflecting the vertical dimension of particles deposited on the substrate. The apparent discrepancy between the AFM height and the lateral particle diameter observed by TEM (∼240 nm) arises because the two techniques probe different geometric dimensions, and partial particle flattening during deposition can further reduce the measured AFM height. The surface roughness (Sa) was measured to be 1.4–2.6 nm (Figure S9), further supporting the textured surface morphology observed by AFM and TEM. At higher magnification, well-defined lattice fringes with an interplanar spacing of 0.34 nm (Figure 2f) confirm the long-range periodic order of the framework, in good agreement with the PXRD results.

**Figure 2:**
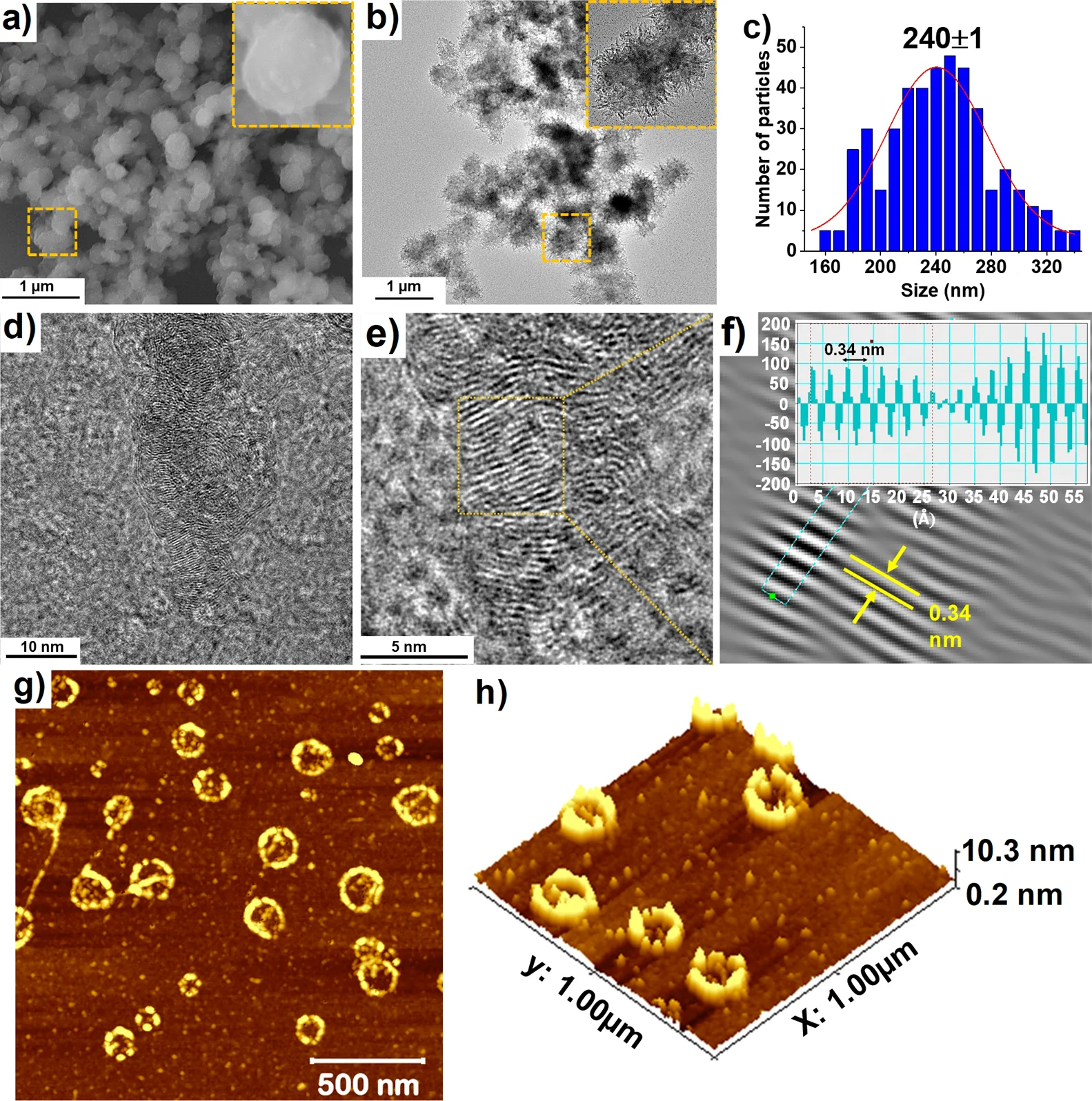
(a) Scanning electron microscopy (SEM) and (b) transmission electron microscopy (TEM) images showing the spiked spherical morphology of the PY-TTA COF. (c) Particle size distribution histogram indicating an average particle size of 240 ± 1 nm. (d, e) High-resolution TEM (HRTEM) images showing the ordered crystalline domains and layer-stacked framework. (f) Lattice fringe analysis confirming an interlayer spacing of 0.34 nm, consistent with π-π stacking between adjacent layers. (g, h) Tapping-mode atomic force microscopy (AFM) images showing well-defined spherical particles bearing nanoscale protrusions, consistent with the spiked morphology observed by HRTEM.

### Guest-Responsive Fluorescence Sensing and Restriction of Host Flexibility

The structural flexibility of the PY-TTA COF was evaluated using dynamic water vapor adsorption–desorption isotherm measurements, which revealed characteristic stepped profiles and pronounced hysteresis loops (Figure 3a). In flexible COFs, such stepped uptake and hysteresis arise from cooperative structural responses to guest adsorption, where the framework undergoes breathing-like pore-opening transitions.^45,46^ These features are consistent with guest-induced structural adaptability in PY-TTA COF. To further probe this behavior, molecular dynamics (MD) simulations^47^ were performed, revealing that the interlayer spacing expands from ∼3.1 Å in vacuum to ∼3.4 Å under aqueous conditions (Figure 3b), confirming water-induced swelling of the framework.

**Figure 3:**
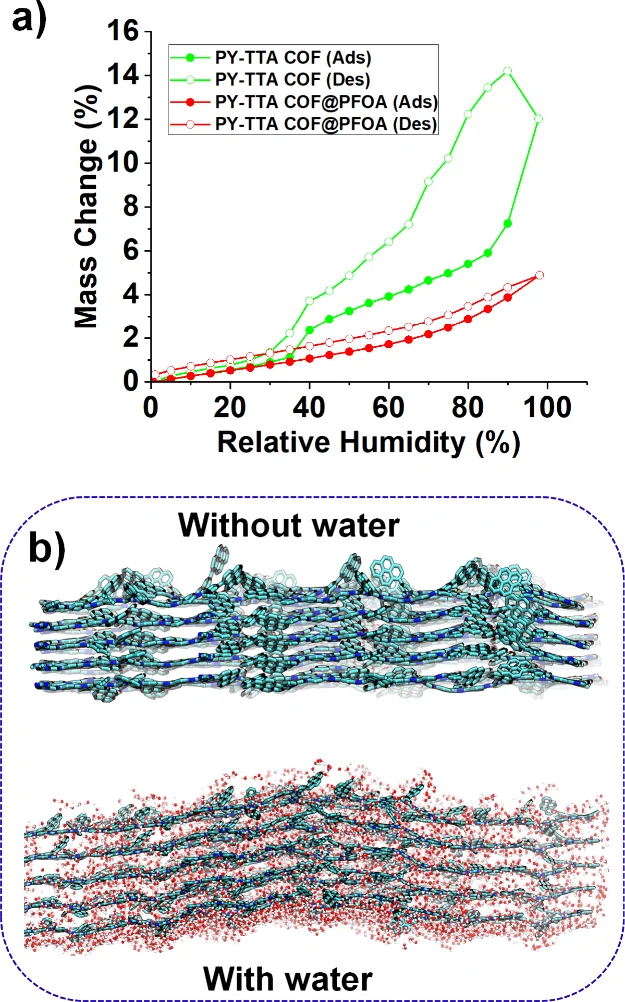
(a) Room-temperature dynamic vapor adsorption–desorption isotherms of PY-TTA COF and PY-TTA COF@PFOA showing characteristic stepped uptake and hysteresis consistent with guest-responsive structural flexibility. (b) MD simulation snapshots of the stacking structure of PY-TTA COF in the absence (top) and the presence (bottom) of water molecules.

The combination of this intrinsic flexibility and the presence of pendent pyrene chromophores projecting into the pore interior motivated us to explore the PY-TTA COF as a guest-responsive luminescent material. When dispersed in water by ultrasonication (500 mg L^–1^) and excited at 365 nm, the COF exhibits an emission centered at 492 nm (Figure 4a). Given the hydrophobic and electron-rich nature of the pyrene fluorophores, we reasoned that the COF might interact strongly with hydrophobic, electron-rich, or highly polarizable guests, leading to fluorescence modulation. The dispersed PY-TTA COF suspension in water remained stable for several days, as confirmed by HRTEM analysis and luminescence studies (Figures S10–S11). No noticeable change in particle size/shape or emission intensity was observed over time, indicating good dispersion stability and fluorescence reproducibility under the experimental conditions.

**Figure 4:**
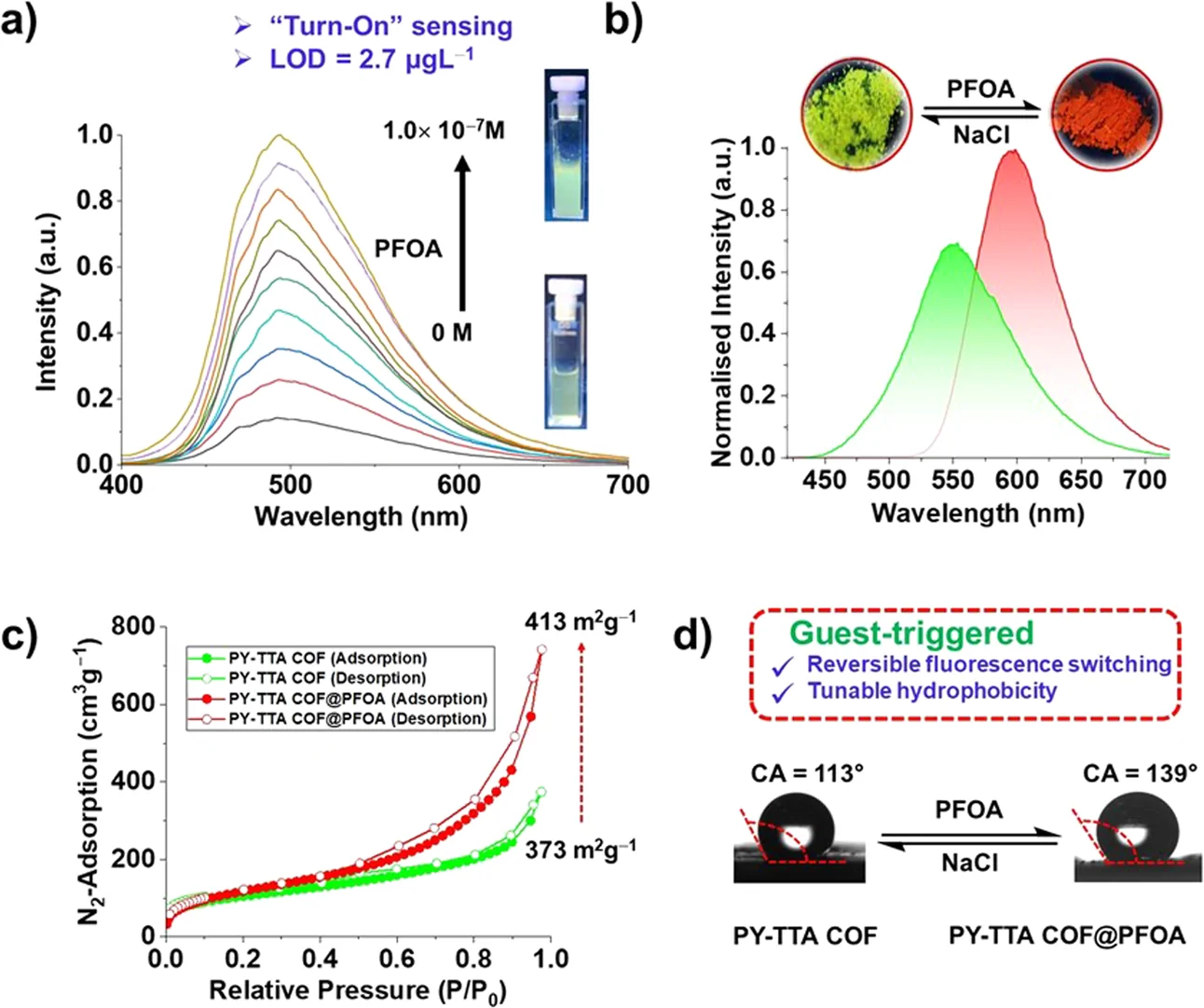
Guest-triggered structural and optical transformations of PY-TTA COF upon PFOA adsorption. (a) Fluorescence ″turn-on″ response of PY-TTA COF to increasing PFOA concentrations. Inset: photographs of aqueous PY-TTA COF dispersions under 365 nm UV light before and after PFOA addition. (b) Solid-state luminescence spectra of PY-TTA COF and PY-TTA COF@PFOA, showing a distinct redshift in emission upon PFOA exposure. Insets: corresponding solid-state emission color changes. (c) N_2_ adsorption–desorption isotherms (77 K) of PY-TTA COF (green) and PY-TTA COF@PFOA (red), highlighting changes in pore accessibility and BET surface area following PFOA adsorption. (d) Water contact angle measurements of PY-TTA COF and PY-TTA COF@PFOA demonstrating increased hydrophobicity following PFOA adsorption and recovery of the original surface wettability after NaCl treatment.

As a proof of concept, we examined the sensing performance of PY-TTA COF toward perfluorooctanoic acid (PFOA), a persistent and toxic environmental contaminant. Upon addition of PFOA, the aqueous PY-TTA COF dispersion displays a clear fluorescence “turn-on” response, with the emission intensity at 492 nm increasing proportionally with PFOA concentration (Figure 4a). The fluorescence response follows a linear calibration (Figure S12), and the limit of detection (LOD), calculated using the 3σ/slope method, was determined to be 2.7 μg L^–1^.

The sensitivity of PY-TTA COF toward PFOA is moderate and comparable to that of other reported fluorescence-based PFOA sensors (Table S1).^7,48,49^ Although the present detection limit does not reach the ng L^–1^ levels required for regulatory compliance monitoring, the rapid fluorescence response and straightforward optical readout make the platform attractive for preliminary screening and contamination assessment applications. Notably, the material exhibits a substantially stronger fluorescence response toward PFOA than toward the other investigated analytes. The fluorescence response of PY-TTA COF toward several fluorinated and nonfluorinated analytes was evaluated (Figures S13 and S14), including perfluorodecanoic acid (PFDA), perfluorohexanoic acid (PFHA), perfluorooctanesulfonic acid (PFOS), perfluorobutanoic acid (PFBA), octanoic acid (OA), and tetrabutylammonium fluoride (TBAF). The PY-TTA COF exhibited substantially weaker fluorescence responses toward these analytes than toward PFOA (Figure S13), consistent with less favorable interactions within the pyrene-functionalized pore environment. Among the PFAS examined, PFDA and PFOS produced weaker fluorescence enhancements than PFOA, indicating that the sensing behavior is not governed solely by fluorocarbon chain length or degree of fluorination.

In addition, common inorganic salts such as NaCl and Na_2_SO_4_ were also examined as potential interferents relevant to contaminated water systems. These salts did not produce any noticeable fluorescence response from the PY-TTA COF (Figures S13 and S14), indicating that the sensing behavior is not significantly influenced by ionic strength or nonspecific electrolyte effects. To further evaluate the sensing performance of PY-TTA COF, additional experiments were conducted in which the interferent compounds were mixed together with PFOA at equal concentrations (Figure S15). As shown in Figure S15, the fluorescence turn-on response induced by PFOA is largely retained in the presence of the competing analytes, indicating minimal interference from coexisting species under the investigated conditions. In addition to long-chain carboxylic acids and common inorganic salts, the interference study was expanded to include representative natural organic matter (NOM) analogues. As shown in Figure S16, representative NOM analogues 2-hydroxynaphthalene and benzoic acid produce only modest fluorescence responses relative to PFOA, and the characteristic PFOA-induced fluorescence enhancement is largely retained in their presence. These results indicate that representative aromatic and carboxylic NOM analogues do not significantly interfere with the PFOA sensing response and that the characteristic fluorescence turn-on behavior is retained in their presence under the investigated conditions. These additional experiments further support the preferential response of the material toward PFOA under more realistic interference conditions. Among the analytes investigated, only PFOA produces both the most pronounced fluorescence enhancement and measurable structural and physicochemical changes in the framework. These results indicate that PY-TTA COF is a promising platform for fluorescence-based detection of PFOA in aqueous media. Time-dependent fluorescence measurements were also performed (Figure S17) by monitoring the evolution of the emission intensity after the addition of PFOA at different concentrations. The fluorescence intensity increased progressively with time and reached a stable plateau within approximately 3 min across the investigated concentration range (Figure S17), indicating rapid host–guest recognition and fluorescence signal generation. Upon PFOA binding, the average fluorescence lifetime increased from 1.81 to 2.96 ns (Figure S18), indicating a measurable change in the excited-state dynamics of the system. To further support this interpretation, temperature-dependent fluorescence measurements were performed (Figure S19). As the temperature decreases, the fluorescence intensity increases, consistent with reduced thermally activated vibrational motion and suppression of local nonradiative relaxation. This trend is consistent with the proposed restriction of local pyrene motion upon guest binding. The recyclability of the fluorescence sensing response in aqueous suspension was evaluated over six consecutive sensing cycles (Figure S20). After each cycle, the PY-TTA COF was regenerated and reused for PFOA detection under identical conditions. The material retained a comparable fluorescence turn-on response throughout the six cycles, with only minor variations in emission intensity, indicating that the sensing performance is largely preserved upon repeated use. These results, together with the PXRD recovery data, demonstrate the robustness and reusability of the PY-TTA COF sensing platform.

To further elucidate the sensing behavior of the material, solid-state photoluminescence measurements were performed on the pristine PY-TTA COF and the PFOA-loaded sample (PY-TTA COF@PFOA). As shown in Figure 4b, excitation at 375 nm results in an emission maximum at 552 nm for PY-TTA COF, which redshifts to 600 nm upon incorporation of PFOA. This shift corresponds to a visible change in emission color from green to red. The redshift is consistent with guest-induced electronic interactions that reduce the calculated HOMO–LUMO energy gap between adjacent COF layers, as determined by DFT calculations (Figure S21), thereby lowering the energy of the emissive transition. This pronounced green-to-red emission shift is observed only in the solid state, where high local PFOA loading within the COF pores can substantially modify the emissive environment. In contrast, the aqueous sensing experiments primarily exhibit fluorescence enhancement with minimal change in emission wavelength, reflecting the more dynamic and solvated nature of the dispersed COF particles.

The structural changes occurring in the COF network upon PFOA adsorption were further examined by HRTEM, which revealed pronounced aggregation of the spiked spherical particles following PFOA exposure (Figure S22). Although increased aggregation is observed following PFOA addition, the fluorescence enhancement is accompanied by distinct spectroscopic and structural signatures of host–guest binding, indicating that aggregation is a consequence of PFOA incorporation rather than being the primary origin of the fluorescence response. To probe surface charge variations, zeta potential (ζ) measurements were performed on aqueous suspensions of PY-TTA COF (Figure S23). The pristine COF exhibits a slightly positive surface charge (ζ = +6.44 ± 4 mV), whereas incorporation of PFOA leads to a substantial decrease to ζ = −26.7 ± 12 mV. This pronounced shift in ζ-potential provides strong evidence for association of anionic PFOA species with the PY-TTA COF surface and pore environment.

To assess the structural response of the framework upon PFOA incorporation, PXRD measurements were performed on PY-TTA COF@PFOA (Figure S24). Although slight changes in peak position and intensity are observed, the characteristic diffraction features of the pristine COF are retained, confirming preservation of framework crystallinity following guest uptake.

To evaluate the reversibility of the solid-state fluorescence switching behavior, PY-TTA COF@PFOA was regenerated using a saturated NaCl/water/methanol solution and subsequently re-exposed to PFOA. As shown in Figure S25, the material retained its characteristic fluorescence color-switching behavior over six consecutive loading–regeneration cycles, demonstrating reversible guest uptake and release within the framework. These results confirm the robustness of the solid-state fluorescence switching process and the reusability of the material under repeated operating conditions. The retention of fluorescence switching behavior over multiple loading–regeneration cycles further supports the reversible nature of the host–guest interaction and complements the recyclability observed in the aqueous sensing experiments (Figure S20). The PXRD pattern of the regenerated material closely matches that of the pristine COF (Figure S26), confirming structural recovery of the framework after repeated regeneration cycles. Compared with the as-synthesized COF, PY-TTA COF@PFOA exhibits clear changes in the relative intensities of the low-angle diffraction features (Figure S24). This behavior is attributed to changes in the relative contributions of partially overlapping reflections, particularly those associated with the calculated (110) and (200) planes, upon guest incorporation. Together with the preservation of the remaining diffraction features, these changes indicate that PFOA incorporation modulates the pore environment while retaining the overall COF structure. This modulation is consistent with guest-induced restriction of local framework dynamics, including the motion of the luminescent pyrene pendants, which contributes to the enhanced fluorescence response of the PFOA-loaded COF.

The strong affinity of PY-TTA COF for PFOA is further supported by the marked alterations in the water sorption behavior (Figure 3a), reflected primarily in the transformation of the isotherm from a Type V profile for the pristine COF into a Type VII profile after PFOA loading. This transition is consistent with a substantially more hydrophobic pore environment arising from the introduction of the perfluoroalkyl chains within the pores and is accompanied by a further suppression of the already low water uptake. In addition, the pronounced hysteresis observed for the pristine framework disappears upon PFOA binding, consistent with guest molecules interacting with the pyrene moieties and restricting local framework dynamics, thereby suppressing the cooperative breathing transitions responsible for the hysteresis response. Inclusion of PFOA within the COF channels leads to a slight reduction in pore diameter, from 1.8 to 1.3 nm (Figure S27), accompanied by minor changes in the N_2_ sorption behavior (Figure 4c), consistent with guest occupation of the pore space while preserving the framework porosity.^50^

The restriction of structural flexibility upon PFOA inclusion is consistent with strong host–guest interactions between PFOA molecules and the PY-TTA COF. These interactions are proposed to involve protonation of the pyridine nitrogen together with hydrophobic C–F···π interactions, in which the fluorinated tail of PFOA aligns with the aromatic π-surfaces of the pyrene moieties. Increased hydrophobicity in PY-TTA COF@PFOA further supports this interaction pattern, as evidenced by surface contact angle (CA) measurements: the pristine COF exhibits a contact angle of 113.0°, which increases to 139.2° after PFOA loading (Figure 4d).

As a control, the structural response of PY-TTA COF toward the isostructural nonfluorinated guest octanoic acid (OA) was examined. In contrast to PFOA, OA induces no detectable structural change, with the PXRD pattern remaining essentially identical to that of the pristine COF (Figure S28). Water sorption isotherms and contact angle analyses were additionally conducted on the OA-treated COF (Figure S29). These measurements did not reveal any significant deviations in sorption characteristics compared to the pristine material. This behavior is consistent with weaker interactions between OA and the pyrene-functionalized pore environment. Similarly, no structural changes were observed upon exposure to shorter-chain fluorinated guests such as PFHA and PFBA (Figure S28), highlighting that the dynamic structural response is most pronounced for PFOA among the analytes examined.

To further emphasize the critical role of pore functionalization in influencing the structural dynamics of the PY-TTA COF, the previously reported pyrene-free analogue, TTA-DFP COF, was evaluated as a reference (structure shown in Figure S31). Following exposure to PFOA, the PXRD pattern of TTA-DFP COF exhibits only minimal changes (Figure S30), indicating that pyrene functionalization is essential for inducing guest-induced structural flexibility and dynamic behavior in the PY-TTA COF. Additional control experiments using TTA-DFP COF (Figure S31) revealed negligible fluorescence changes upon PFOA addition. The absence of a pronounced fluorescence response in the pyrene-free analogue indicates that the fluorescence enhancement observed for PY-TTA COF is not solely a consequence of guest-induced aggregation or framework reorganization, but instead requires the pyrene-functionalized pore environment. Taken together, these results highlight the importance of the pyrene pendants in mediating both the structural and photophysical responses of the framework toward PFOA.

The interactions between PFOA and PY-TTA COF were further investigated by solid-state NMR spectroscopy (Figure 5). The ^13^C CP/MAS spectra of the pristine COF and the PFOA-loaded sample (Figure 5b) show no major chemical shift changes for the framework carbons, indicating that the overall COF structure remains intact upon guest binding. However, closer inspection of the aromatic region (Figure 5b) reveals a small but reproducible shift (∼0.5 ppm) for the most shielded aromatic carbon at ∼116 ppm, assigned to the pyridine carbon. This subtle perturbation suggests involvement of the pyridine units in the host–guest interaction. In contrast, the chemical shifts associated with the triazine carbon atoms (∼169.3 ppm) remain unchanged, indicating negligible perturbation of the triazine ring environment. The ^13^C CP/MAS spectrum of the PY-TTA COF@PFOA complex also displays characteristic signals of the guest molecule, including the carbonyl carbon (∼165 ppm) and CF_2_ resonances (∼110 ppm), confirming successful incorporation of PFOA into the COF.

**Figure 5:**
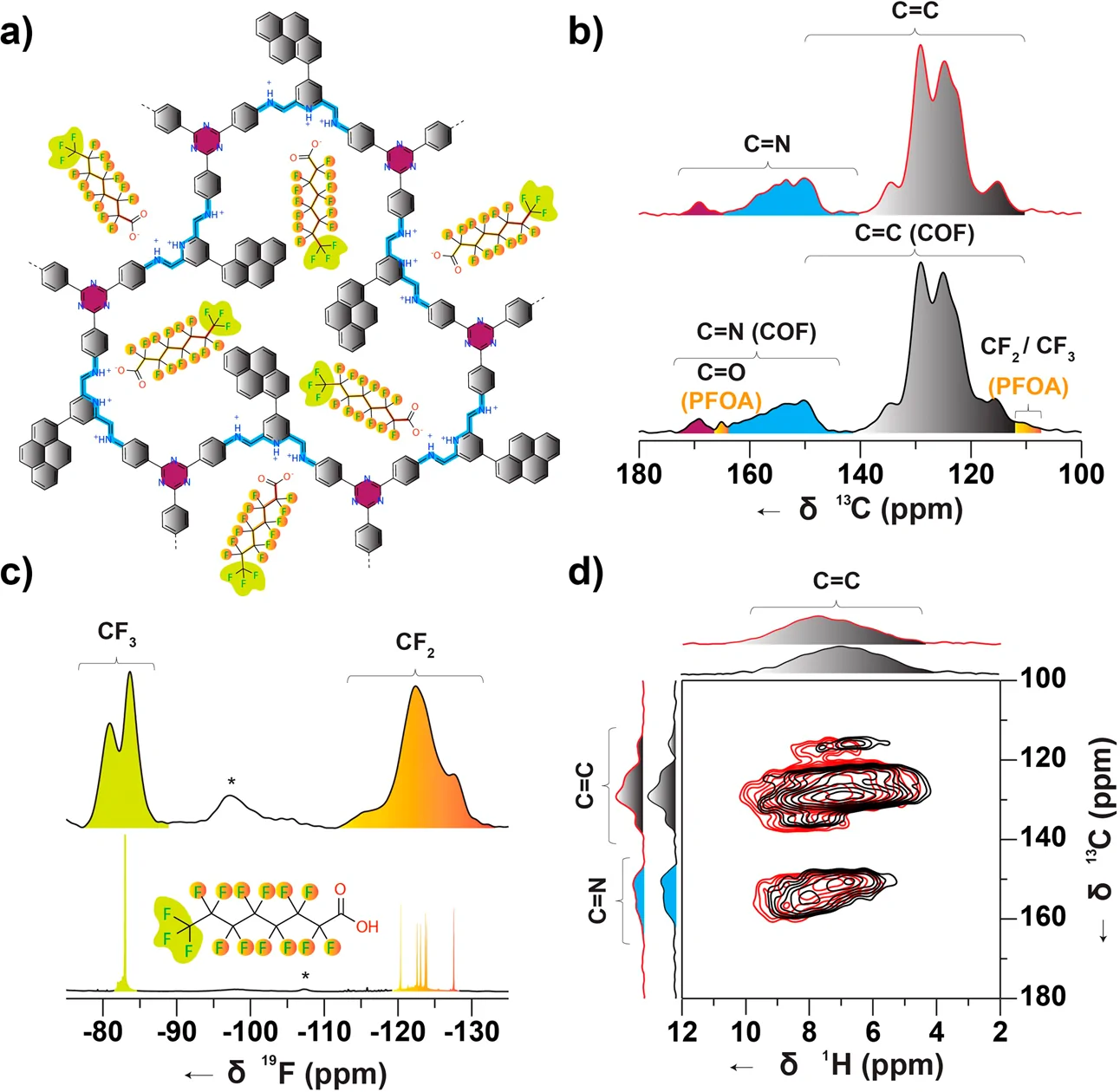
Solid-state NMR characterization of PFOA binding to PY-TTA COF. (a) Simplified schematic illustration showing the interaction of PFOA molecules with the PY-TTA COF framework. (b) Stacked one-dimensional fast-MAS ^13^C CP/MAS solid-state NMR spectra of PY-TTA COF (black, bottom) and PY-TTA COF@PFOA (red, top). (c) Stacked one-dimensional ^19^F solid-state NMR spectra of pure PFOA (bottom) and PY-TTA COF@PFOA (top). (d) Overlay of the two-dimensional fast MAS ^13^C-^1^H HETCOR solid-state NMR spectra of PY-TTA COF (black contours) and PY-TTA COF@PFOA (red contours). Asterisks (*) mark spinning sidebands.

To further monitor changes in fluorine environments, ^19^F solid-state NMR spectra were collected for pure PFOA and the PY-TTA COF@PFOA complex (Figure 5c), providing additional confirmation of host–guest interactions. The ^19^F MAS NMR spectrum of pure PFOA is well resolved, displaying sharp resonances from the terminal CF_3_ group (∼−83 ppm) and the internal CF_2_ units (−120 to −130 ppm). The narrow line shapes reflect significant molecular mobility, which averages out anisotropic interactions under MAS. In contrast, the ^19^F spectrum of the PY-TTA COF@PFOA exhibits markedly broadened resonances. The CF_3_ signals appear between −77 and −87 ppm, while the CF_2_ signals span a wider region (−110 to −130 ppm), accompanied by prominent spinning sidebands. This broadening indicates a substantial reduction in motional averaging upon binding to the COF, consistent with restricted mobility of PFOA within the confined pore environment. The broadened ^19^F features also reflect the heterogeneous local chemical environments experienced by PFOA, arising from strong host–guest interactions, including C–F···π contacts and electrostatic interactions, and from intermolecular noncovalent contacts within the pores. The distribution of binding geometries produces a wide range of ^19^F chemical shifts. Collectively, the loss of motional averaging, the appearance of sidebands, and the broadening/splitting of ^19^F signals indicate that PFOA molecules experience restricted mobility and diverse local magnetic environments within the PY-TTA COF. To probe local proton environments and spatial proximities, two-dimensional ^13^C–^1^H HETCOR spectra were acquired using ^1^H-detected fast MAS (40 kHz). Comparison of the HETCOR spectra for the pristine COF and the COF–PFOA complex (Figure 5d) corroborates the small shift of the ∼116 ppm pyridine carbon, consistent with the 1D data. More pronounced differences are observed in the proton dimension: the ^1^H chemical shifts of PY-TTA COF exhibit an overall downfield shift (∼1 ppm) upon PFOA binding. This deshielding is consistent with hydrogen-bonding and/or dipolar interactions between aromatic or imine protons of the COF and electronegative atoms (F or O) within PFOA. These spectral changes indicate that PFOA binding primarily affects the local environments of the aromatic and imine functionalities within the framework. The NMR results are consistent with guest-induced structural rearrangements, potentially involving changes in interlayer organization and π–π interactions, as also suggested by the PXRD and TEM analyses.

To evaluate changes in the nitrogen environments upon PFOA binding, ^15^N CP/MAS solid-state NMR spectra were collected for both the pristine PY-TTA COF and the PY-TTA COF@PFOA (Figure 6a). The spectrum of the pure COF displays two dominant resonances: one arising from triazine nitrogens (∼248.5 ppm) and another corresponding to the pyridine and imine nitrogens (∼335.8 ppm). In contrast, the spectrum of the PFOA-loaded COF shows the emergence of a new nitrogen resonance at ∼124.2 ppm. The appearance of this additional resonance indicates strong host–guest interactions involving protonation and/or hydrogen bonding at the more basic nitrogen sites (pyridine and/or imine) by the carboxylic acid group of PFOA, consistent with previously reported proton-transfer interactions between perfluorinated carboxylic acids and basic nitrogen centers.^51,52^ Notably, the chemical shifts associated with the triazine nitrogens remain unchanged upon PFOA incorporation, indicating that these nitrogens are minimally affected by the interaction, an observation that aligns with the ^13^C CP/MAS NMR data. The observed ^15^N chemical shift perturbations correlate well with the relative basicities of the nitrogen atoms in the COF. The pyridine and imine nitrogens are substantially more basic than those of the triazine units and therefore more prone to protonation or strong hydrogen bonding with PFOA. These results suggest that pyridine and imine functionalities play a key role in mediating PFOA binding, while the triazine centers remain largely uninvolved. This interpretation is consistent with the minimal perturbation observed for the triazine ^15^N resonance and with the lower basicity generally associated with s-triazine nitrogen atoms relative to pyridine and imine functionalities.^53^

**Figure 6:**
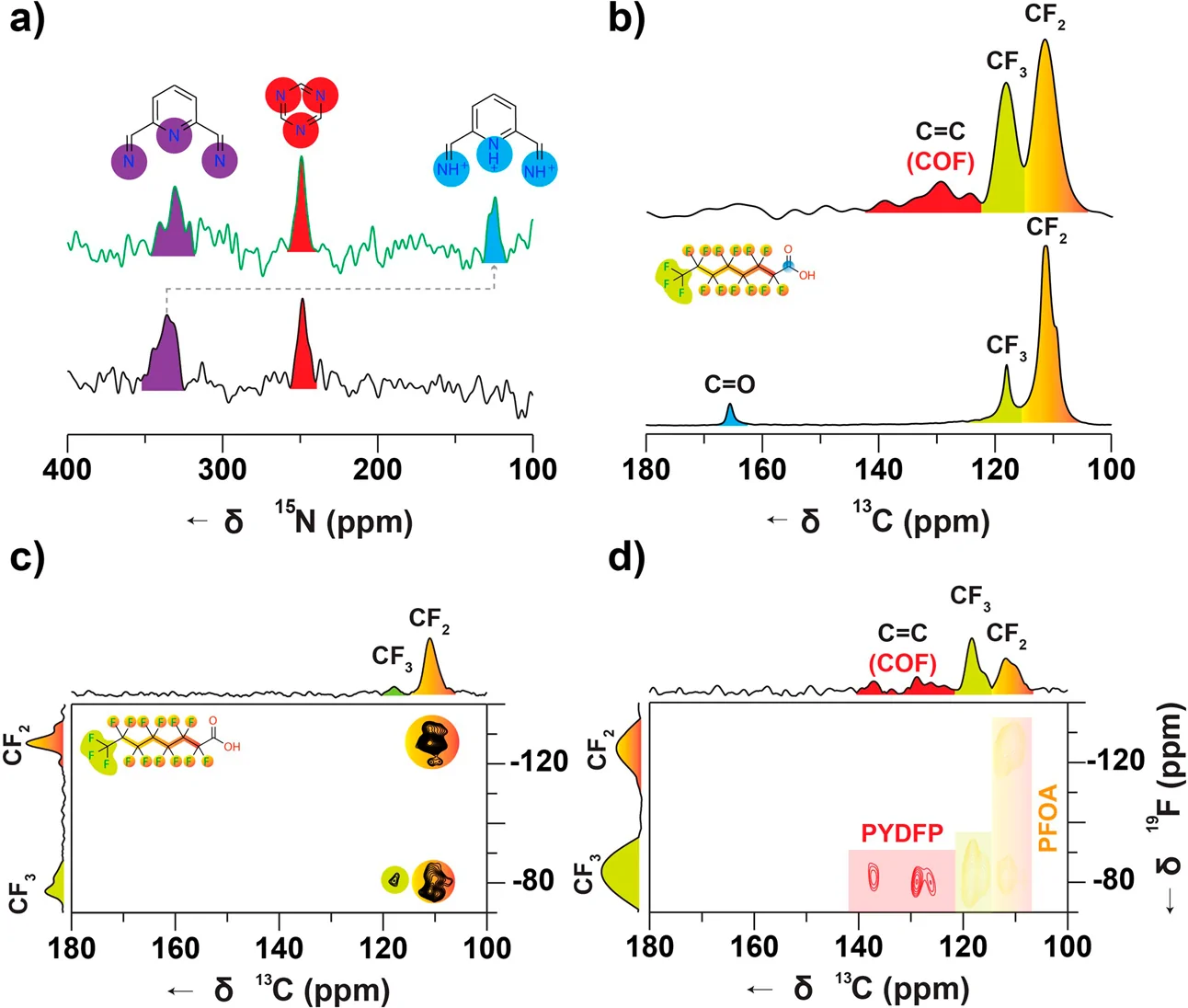
Solid-state ^15^N and ^19^F–^13^C NMR analyses revealing the binding interactions between PFOA and PY-TTA COF. (a) Stacked ^15^N CP/MAS solid-state NMR spectra of PY-TTA COF (black, bottom) and PY-TTA COF@PFOA (green trace, top) highlighting potential nitrogen-based interaction sites. (b) Stacked ^19^F→^13^C CP/MAS solid-state NMR spectra comparing pure PFOA (bottom) and PY-TTA COF@PFOA (top), (c) two-dimensional ^19^F–^13^C HETCOR solid-state NMR spectrum of pure PFOA illustrating fluorine–carbon correlations in the absence of the COF framework, and (d) two-dimensional ^19^F–^13^C HETCOR solid-state NMR spectrum of PY-TTA COF@PFOA, showing the specific ^19^F–^13^C interactions within the COF environment after PFOA adsorption.

The interaction mechanism between PFOA and PY-TTA COF was further investigated by fast MAS (40 kHz) ^1^H solid-state NMR experiments (Figure S32). The ^1^H spectrum of pristine PY-TTA COF displays aromatic proton resonances at ∼6.9 ppm and signals from adsorbed solvents at ∼2.5 ppm. In contrast, the spectrum of the PY-TTA COF@PFOA exhibits a new high-frequency resonance at ∼16.8 ppm, characteristic of strongly hydrogen-bonded carboxylic protons from PFOA. The aromatic proton signals also shift slightly downfield (∼7.9 ppm), while the solvent peaks remain unchanged (∼2.5 ppm). The appearance of the ∼16.8-ppm peak provides direct evidence of strong hydrogen-bonding interactions between the PFOA carboxyl group and the basic nitrogen sites (pyridine/imine) of the COF.

Furthermore, to probe spatial proximities and intermolecular contacts, fast MAS (40 kHz) ^1^H–^1^H NOESY-type solid-state NMR experiments were performed (Figure S33). The spectrum of PY-TTA COF reveals dominant correlation peaks between aromatic protons (∼7 ppm) and the adsorbed solvent (∼2.6 ppm). Upon PFOA binding, additional cross-peaks emerge at ∼16.9/6 ppm, correlating the hydrogen-bonded PFOA proton (∼16.9 ppm) with the aromatic protons of the COF (∼6 ppm), while the original aromatic-solvent correlations (∼7/2.6 ppm) remain present. These newly observed correlations confirm the close spatial proximity of PFOA’s carboxylic group to the COF’s aromatic framework, corroborating the hydrogen-bonding interaction inferred from the ^1^H NMR data. Together, these results provide compelling evidence that PFOA binds to PY-TTA COF through hydrogen-bonding interactions and possible proton-transfer processes involving the pyridine and/or imine functionalities. To assess the influence of pH on the fluorescence response of PY-TTA COF, photoluminescence measurements were conducted over a wide pH range (pH 1, 2, 3, 7, 10, and 11). As shown in Figure S34, only modest variations in fluorescence intensity were observed across the investigated pH range. Importantly, these changes are substantially smaller than the fluorescence enhancement observed upon PFOA binding. These results indicate that the sensing response cannot be attributed to simple pH-induced effects or proton-transfer processes. Instead, they support the proposed mechanism involving specific host–guest interactions between PFOA and the PY-TTA COF, which are proposed to restrict the local motion of the pendant pyrene groups and enhance fluorescence emission.

To probe through-space proximities between fluorine and carbon sites, ^19^F→^13^C CP/MAS and 2D ^19^F–^13^C HETCOR solid-state NMR experiments were performed on pure PFOA and PY-TTA COF@PFOA (Figure 6b–d). The ^13^C CP/MAS spectrum of pure PFOA (Figure 6b, bottom) is well resolved, exhibiting characteristic resonances from the CF_3_ carbon (∼118 ppm), the CF_2_ carbons (∼111 ppm), and the carbonyl carbon (∼165.5 ppm). In the PY-TTA COF@PFOA spectrum (Figure 6b, top), these guest-derived peaks are observed alongside the aromatic carbons of the COF (120–140 ppm), confirming successful incorporation of PFOA into the framework.

The 2D ^19^F–^13^C HETCOR spectrum of pure PFOA (Figure 6c) displays strong intramolecular correlations: CF_3_ F/CF_3_ C (−81/118 ppm), CF_2_ F/CF_2_ C (−127/111 ppm), and cross-correlations between CF_3_ fluorine and CF_2_ carbon sites (−81/111 ppm). In contrast, the HETCOR spectrum of the PY-TTA COF@PFOA (Figure 6d) reveals new intermolecular correlations between the CF_3_ fluorine atoms of PFOA and the aromatic carbons of the pyrene units in the COF, indicating close spatial proximity and supporting the presence of C–F···π interactions. Notably, no intermolecular correlations are detected between CF_2_ fluorine atoms and COF aromatic carbons, suggesting a preferred orientation of the PFOA tail in which the terminal CF_3_ group lies closest to the pyrene surfaces, while the internal CF_2_ segments remain more distant.

Together, the comprehensive solid-state NMR results combined with PXRD, TEM, and sorption measurements provide a coherent molecular-level picture of the interaction between PFOA and PY-TTA COF. The binding mechanism is proposed to involve a synergistic combination of (i) hydrogen-bonding interactions and possible proton-transfer processes between the PFOA carboxyl group and the basic pyridine/imine nitrogen sites of the COF, and (ii) hydrophobic and C–F···π interactions between the fluorinated tail of PFOA and the pyrene moieties. These cooperative interactions are consistent with confinement of PFOA within the pore environment and restriction of the dynamic motions of the pendant pyrene units, while inducing supramolecular-level structural rearrangements, such as changes in stacking, increased local ordering, and partial pore contraction, without disrupting the integrity of the COF framework. Collectively, these observations support a model in which specific host–guest interactions couple molecular recognition to the observed fluorescence response.

Semiempirical calculations based on the GFN2-xTB method (SI), followed by single-point energy DFT calculations using the r^2^SCAN-3c composite method combined with the SMD solvation model, were carried out to examine the interactions between PFOA and the PY-TTA COF and to identify the most favorable adsorption sites. The structural model employed in the calculations is shown in Figure S35, while the optimized adsorption geometries and relative energies are summarized in Figure 7, Figures S36–S37, and Tables S2–S3. The calculations show that the most thermodynamically stable adsorption configuration involves proton transfer from PFOA to an imine nitrogen of the COF. Protonation of the pyridine or triazine nitrogen sites is also possible but is energetically less favorable by ∼ 16 and ∼ 43 kJ mol^–1^, respectively, than protonation of the imine nitrogen. All binding configurations are stabilized by a combination of strong hydrogen-bonding and dispersive C–F···π interactions. In particular, dispersion contacts are observed between the aryl C–H groups of the pyrene moieties and the CF_3_ terminus of PFOA, while hydrogen bonds form between the carboxylate group of deprotonated PFOA and the protonated imine/pyridine/triazine sites.

**Figure 7:**
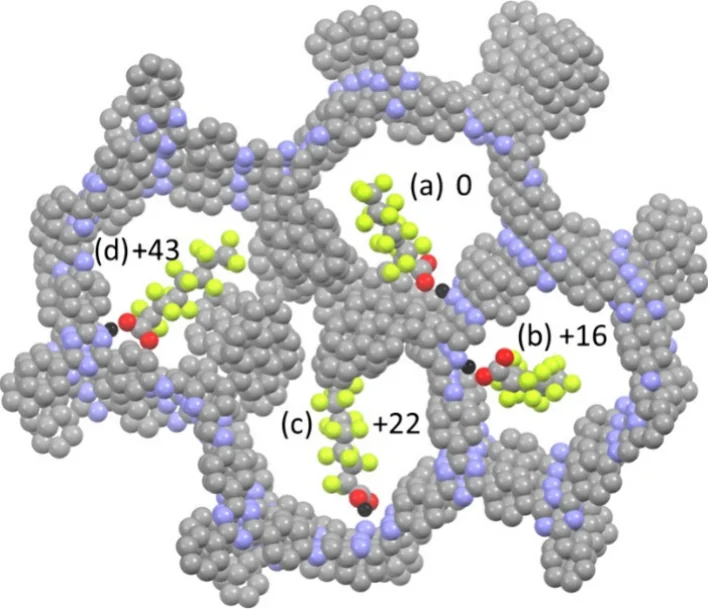
GFN2-xTB–optimized adsorption geometries of PFOA within the PY-TTA COF and the corresponding single-point DFT relative energies (kJ mol^–1^) calculated using the r^2^SCAN-3c/def2-mTZVPP with the SMD solvation model. (a) Most stable configuration: proton transfer from PFOA to an imine nitrogen, forming a deprotonated PFOA species. (b) Proton transfer to a pyridine site (+16 kJ mol^–1^). (c) Neutral PFOA hydrogen-bonded to a pyridine nitrogen (+22 kJ mol^–1^). (d) Proton transfer to a triazine site (+43 kJ mol^–1^). In all panels, PFOA is shown in the optimized binding orientation, and the transferable proton is highlighted in black.

In the third most stable adsorption mode, which is higher in energy by ∼22 kJ mol^–1^, PFOA retains its proton and forms a hydrogen bond with a pyridine nitrogen. The hierarchy of adsorption energies and the nature of the predicted interactions are in good agreement with the solid-state NMR observations (Figures 5 and 6), which collectively support the involvement of hydrogen-bonding interactions, possible proton-transfer processes, and C–F···π contacts in the host–guest interaction.

Although DLS and TEM measurements reveal increased aggregation following PFOA addition, the combined experimental and computational results suggest that this aggregation is a consequence of PFOA binding rather than the primary cause of the fluorescence enhancement. In conventional aggregation-induced emission (AIE) systems, fluorescence enhancement typically arises from nonspecific aggregation. In contrast, the fluorescence response of PY-TTA COF is accompanied by distinct spectroscopic, structural, and computational signatures of specific host–guest interactions. PXRD, solid-state NMR, fluorescence lifetime measurements, adsorption studies, and GFN2-xTB calculations collectively support a model in which PFOA binding restricts the local motion of the pendant pyrene groups within the COF pores. Because the pyrene units are covalently anchored within the framework, the fluorescence enhancement is attributed primarily to guest-induced restriction of local pyrene motion, while aggregation appears to be a secondary consequence of the host–guest recognition process.

## Conclusion

In summary, we have shown that the pyrene-functionalized PY-TTA COF undergoes a pronounced guest-induced fluorescence enhancement upon interaction with PFOA. The combination of a flexible porous framework and pendent pyrene chromophores enables specific host–guest interactions that restrict local molecular motion and produce a robust fluorescence “turn-on” response while retaining the crystallinity of the COF. Solid-state NMR spectroscopy provides molecular-level insight into the binding process, revealing hydrogen-bonding interactions, possible proton-transfer processes involving pyridine and imine nitrogen sites, and C–F···π contacts that position the fluorinated PFOA tail in close proximity to the pyrene units. Complementary PXRD, TEM, DLS, sorption, fluorescence lifetime, and temperature-dependent fluorescence measurements demonstrate that PFOA binding modulates local structural dynamics and alters the pore environment without compromising the covalent integrity of the framework.

The resulting fluorescence enhancement and the detection limit of 2.7 μg L^–1^ demonstrate the potential of PY-TTA COF for fluorescence-based detection of PFOA in aqueous media. More broadly, this work establishes a design strategy in which controlled molecular flexibility and strategically oriented emissive units can be leveraged to generate responsive optical behavior in COFs. By coupling molecular recognition with photophysical amplification through controlled local dynamics, this study provides a framework for the development of stimuli-responsive COFs with tunable optical properties for sensing and environmental remediation applications. Beyond PFAS sensing, this work suggests that controlling local molecular dynamics inside COFs may provide a general strategy for developing of stimuli-responsive porous materials. The combination of flexible framework architectures with pendent emissive units may be extended to other molecular recognition processes in which guest-induced restriction of motion can be translated into amplified optical responses.

## Supplementary Material



## Notes

Detailed experimental protocols, complete characterization of the COF materials (Figure S1–S37), luminescence sensing performance, mechanistic insights into guest-induced vibration locking, and theoretical calculations supporting the proposed sensing mechanism are provided in the Supporting Information. (PDF)
