# Supplementary material for: Guest-Induced Vibration Locking and Fluorescence Switching in a Pyrene-Functionalized Covalent Organic Framework (COF) for Perfluorooctanoic Acid (PFOA) Recognition
Source: J Am Chem Soc. 2026 Aug 28;148(35):37651–62. doi: 10.1021/jacs.6c01527 (PMC13564431; doi:10.1021/jacs.6c01527)
Supplement: Supplementary file 1 [file ja6c01527_si_001.pdf]

Guest-Induced Vibration Locking and Fluorescence Switching in a Pyrene-Functionalized COF for PFOA Recognition

Gobinda Das,[a ¶] Thirumurugan Prakasam,[a ¶] Nour Alkhatib,[a] Sudhir Kumar Sharma,[b] Hoda Al-assaad,[c] Farah Benyettou,[a] Rasha G. AbdulHalim,[a] Hamzeh Sabouni,[d] Kareem Mazen,[a] Ramesh Jagannathan,[b] Charles J.-A. Jackson,[e] Felipe Gándara,[f] Andreas Mavrandonakis,[f] Mark A. Olson,[e] Samer Aouad,[a,g] Mohamad El-Roz,[c] Serdal Kirmizialtin,*[a] Sabu Varghese,*[h] and Ali Trabolsi*[a,i]

[a] Science Division, New York University Abu Dhabi, Saadiyat Island, PO Box 129188, Abu Dhabi, United Arab Emirates.

[b] Engineering Division, New York University Abu Dhabi, Saadiyat Island, PO Box 129188, Abu Dhabi, United Arab Emirates.

[c] ENSICAEN, UNICAEN, CNRS, Laboratoire Catalyse et Spectrochimie, Normandie Université, Caen 14000, France

[d] Materials Science and Engineering Program, College of Arts and Sciences, American University of Sharjah, POB 26666, Sharjah, United Arab Emirates

[e] Department of Physical and Environmental Sciences, Texas A&M University Corpus Christi, 6300 Ocean Dr., Corpus Christi, TX 78412 USA.

[f] Instituto de Ciencia de Materiales de Madrid-CSIC, C. Sor Juana Inés de la Cruz 3, 28049 Madrid, Spain.

[g] Department of Chemistry, Faculty of Arts and Sciences, University of Balamand, PO Box 100, Tripoli, Lebanon.

[h] CTP, New York University Abu Dhabi, Saadiyat Island, PO Box 129188 Abu Dhabi, United Arab Emirates.

[i] Water Research Center, New York University Abu Dhabi, Saadiyat Island, PO Box 129188, Abu Dhabi, United Arab Emirates.

Supporting Information

¶ Equal Contribution

<u>**Table of Contents**</u>

1. Materials and Methods

2. Molecular modelling setup for the COF simulations

3. Synthetic Procedures for Monomers and PY-TTA COF

4. Characterization

Figure S1. FT-IR analysis of the precursor and PY-TTA COF

Figure S2. ^{13}C CP/MAS solid-state NMR spectra of precursor and PY-TTA COF

Figure S3. PXRD- analysis

Figure S4. BET analysis

Figure S5. Non-Local Density functional theory (NLDFT)-derived pore size distribution of PY-TTA COF

Figure S6. Scanning electron microscopy (SEM) analysis of PY-TTA COF

Figure S7. Transmission electron microscopy (TEM) and high-resolution TEM (HRTEM) analysis of PY-TTA COF

Figure S8. Tapping-mode atomic force microscopy (AFM) analysis of PY-TTA COF.

Figure S9. AFM surface roughness study.

5. Photophysical studies

Figure S10. Fluorescence stability test.

Figure S11. Morphological stability of the PY-TTA COF.

Figure S12. Calibration plot for detection limit calculations.

Figure S13. Comparison of the fluorescence response of PY-TTA COF to PFOA, related PFAS, and inorganic salts

Figure S14. Emission spectra of PY-TTA COF with PFOA, PFAS, TBAF, and inorganic salts

Figure S15. Selective fluorescence response of PY-TTA COF toward PFOA in the presence of competing analytes

Figure S16. Fluorescence response of suspended PY-TTA COF to PFOA in the presence of NOM

Figure S17. Time-dependent fluorescence response of PY-TTA COF

Figure S18. Time-resolved fluorescence decay profiles of (a) PY-TTA COF and (b) PY-TTA COF@PFOA

Figure S19. Variable-temperature fluorescence spectra of PY-TTA COF

Figure S20. Recyclability of the PY-TTA COF

6. DFT-study

Figure S21. DFT-calculated HOMO–LUMO energy diagrams

7. HRTEM and DLS analysis

Figure S22. HRTEM images of PY-TTA COF before and after PFOA exposure

Figure S23. Dynamic light scattering (DLS) size distribution profiles

8. Structural stability study

Figure S24. Powder X-ray diffraction (PXRD) patterns of pristine PY-TTA COF and PFOA-loaded PY-TTA COF@PFOA

9. Solid-state luminescent and PXRD analysis

Figure S25. Reversible solid-state fluorescence switching of PY-TTA COF

Figure S26. Powder X-ray diffraction (PXRD) patterns of pristine PY-TTA COF and regenerated PY-TTA COF

10. Pore size distribution

Figure S27. NLDFT-derived pore size distribution plots of PY-TTA COF and PY-TTA COF@PFOA

11. PXRD analysis of PY-TTA COF with other PFOA analogues

Figure S28. Powder X-ray diffraction (PXRD) patterns of PY-TTA COF and its guest-loaded analogues

12. Water adsorption analysis and PXRD analysis

Figure S29. Water adsorption–desorption isotherms of PY-TTA COF@OA

Figure S30. Powder X-ray diffraction (PXRD) patterns of TTA-DFP COF and TTA-DFP COF@PFOA

13. Control luminescent study of PY-TTA COF analogue TTA-DFP COF

Figure S31. Chemical structure and Fluorescence emission spectra of TTA-DFP COF before and after PFOA treatment

14. Solid-state proton NMR and NOESY spectra

Figure S32. ^{1}H solid-state NMR

Figure S33. NOESY

15. pH dependent luminescent study

Figure S34. Fluorescence emission spectra of PY-TTA COF recorded at different pH values

16. Semiempirical and DFT calculations

Figure S35. Structural model of PY-TTA COF

Figure S36. Lowest-energy adsorption configurations obtained by initially considering neutral PFOA within the pores of the PY-TTA COF model using GFN2-xTB calculations

Figure S37. Lowest-energy adsorption configurations of deprotonated PFOA within the pores of the PY-TTA COF model obtained from GFN2-xTB calculations

17. References

1. Materials and Methods

All reagents and starting materials were purchased from Sigma-Aldrich and used without further purification. Deionized water was obtained from a Millipore Gradient Milli-Q water purification system. Thin-layer chromatography (TLC) was performed on silica gel 60 F254 (E. Merck). The plates were inspected under the UV light. Column chromatography was performed on silica gel 60F (Merck 9385, 0.040–0.063 mm). Routine nuclear magnetic resonance (NMR) spectra were recorded at 25 °C on a Bruker Avance spectrometer, with a working frequency of 500 MHz for ^{1}H and 125 MHz for ^{13}C nuclei. All chemical shifts are reported in ppm relative to the signals corresponding to the residual non-deuterated solvents (CD_3CN: δ = 1.94 ppm, CD_3OD: δ = 3.31 ppm, D_2O: δ = 4.97 ppm and DMSO-d_6: δ = 2.50 ppm). Coupling constant values (*J*) are given in hertz (Hz), the multiplicity is abbreviated in the following way: s (singlet) and d (doublet). Fourier transform infrared (FTIR) studies were carried out on the Agilent 670-IR spectrometer. Magic Angle Spinning (MAS) solid-state NMR experiments were carried out on a Bruker Avance-HD 600 MHz spectrometer operating at a static field of 14.1 T using a 4.0 mm MAS probe. Powdered dry samples were packed into 4.0 mm zirconia rotors and were spun at a MAS frequency of 14 kHz. Cross-Polarization Magic Angle Spinning (CP/MAS) experiments were performed using standard linearly ramped cross-polarization pulse sequence. ^{13}C chemical shifts were externally referenced to the adamantane CH_2 signal at 38.48 ppm on the TMS scale. ^{19}F chemical shifts were referenced using polytetrafluoroethylene (PTFE) as a secondary reference (δ = −122.7 ppm). ^{15}N chemical shifts were referenced indirectly to liquid ammonia by using glycine as an external secondary reference at (δ = 34.4 ppm). NMR data were processed using TopSpin software. Scanning electron microscopy (SEM) images were obtained from FEI Quanta 450FEG. The topography of the PY-TTA COF material was analyzed by dynamic atomic force microscopy (5500 Atomic Force Microscope; Keysight Technologies Inc., Santa Rosa, CA). We acquired topography, phase, and amplitude scans simultaneously. Silicon cantilevers (Nanosensors™, Neuchatel, Switzerland) with resonant frequencies of 250–300 kHz and force constants of 100–130 N m^{-1} were used. The set point value was kept at 2.5 V. AFM scans were collected at 1024 points/lines with scan speed of 0.20 at fixed scan angle of 0°. Scan artifacts were minimized by acquiring a typical scan at an angle of

90° under identical image acquisition parameters. We used GwyddionTM free software (version 2.47), SPM data visualization and analysis tool for post-processing the AFM scans. Size and morphology of the PY-TTA COF film were determined with a TEM (FEI-Titan 300) microscope. Samples were prepared on a carbon-coated copper grid. A drop of dispersed materials was spotted on the grid and allowed to dry overnight. Powder X-ray diffraction (PXRD) measurements were carried out using the PANalyticalX'Pert PRO MP X-ray diffractometer consisting of a focusing elliptical mirror and a fast-high resolution detector (PIXCEL) with the radiation wavelength of 0.15418 nm. Water vapor adsorption-desorption experiments were carried out to examine water vapor adsorption characteristics of PY-TTA COF using IGAsorp vapor sorption analyzer. The water vapor partial pressure was controlled automatically by mixing wet vapor feed with a dry N_2 line; thus, N_2 acts as a carrier gas for water vapor. Pre-drying of the sample was carried out at 100 °C in the presence of N_2 dry carrier. The sample "dry mass" was measured under N_2 and was at equilibrium (25 °C) before dosing water vapor progressively into the chamber. The adsorption isotherms, obtained at equilibrium, were collected between 0 and 98 % RH at room temperature. The maximum equilibration time for each RH was maintained for 2 h. Fluorescence measurements were recorded on Horiba Scientific, Fluoromax spectrofluorometer. Dynamic light scattering (DLS) measurements were performed on a Malvern Zetasizer NanoSeries to determine the Zeta(ζ)-potential as well as the hydrodynamic size of the particles.

2. Molecular modelling setup for the COF simulations

The COF unit cell was extended to create a supercell of 5 × 5 ×10. The framework was placed at the center of a 30 × 30 × 14 nm box and subsequently solvated with water molecules. The TIP3P model[1] was employed to represent the water molecules, while the Dreiding force field[2] was applied to represent the COF framework. Partial charges for COF were assigned using the Gasteiger method. The computational model has been shown to reliably replicate adsorption processes, as supported by our earlier studies.[3-5]

Molecular dynamics simulations

All simulations were performed using GROMACS.[6] The first stage involved a 1 μs molecular dynamics simulation of the COF structure in a vacuum to allow full structural relaxation. The final frame from this simulation was used as the starting point for the second stage, where the COF was solvated with water. The simulation system was first optimized using the steepest descent for 500 steps.[7] Following minimization, a 5 ns equilibration was performed in the NPT ensemble to stabilize the system's density and pressure. Subsequently, production simulations were carried out in the NVT ensemble for 300 ns using a leapfrog integrator.[8] Long-range interactions were treated with a cut-off of 1 nm for both electrostatic and van der Waals interactions. The Particle Mesh Ewald Summation method[9] was used to compute electrostatic interactions. A detailed description of the simulation details can be found in our previous works[3-5].

Quantum mechanical calculations

To compute the HOMO-LUMO levels, we utilized Gaussian.[10] We optimized the geometry of the structures using the B3LYP method and a 6-311G(d,p) basis set. Following optimization, the HOMO and LUMO were extracted from the electronic structure calculation.

3. Synthetic Procedures for Monomers and PY-TTA COF materials

4,4',4''-(1,3,5-triazine-2,4,6-triyl)trianiline (TTA): The triamine (TTA) was synthesized according to the published procedure with no modifications.[11]

4-(pyren-2-yl)pyridine-2,6-dicarbaldehyde (PY):

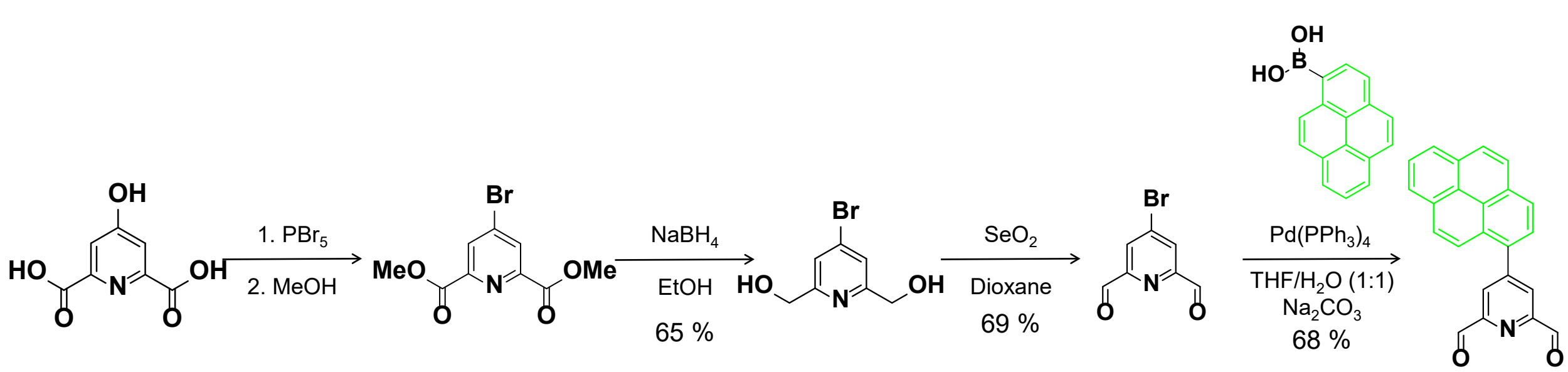

2 3 4 PyDFP

Scheme 1: synthetic protocol for preparing 4-pyrenepyridine-2,6-dicarbaldehyde

(**PyDFP**) involves starting with chelidamic acid and utilizing Suzuki coupling in the final step.

Synthesis of dimethyl-4-bromopyridine-2,6-dicarboxylate (2): To synthesize Compound 2, a method from the literature was adapted with slight modification.[12] In a 250 mL round-bottom flask fitted with a reflux condenser, 3.0 g of chelidamic acid was mixed with 15.0 g of phosphorus pentabromide. This solid mixture was then heated to 90 °C with vigorous stirring for 3 hours. After the reaction was complete, the mixture was cooled to room temperature, and 60 mL of anhydrous chloroform ($CHCl_3$) was added. Subsequently, 20 mL of anhydrous methanol (MeOH) was added dropwise into the reaction mixture at 0 °C while stirring vigorously, which led to the formation of a white precipitate. This precipitate was collected through vacuum filtration, washed with methanol, and dried under reduced pressure overnight, yielding Compound **2** as a white powder (3.51 g, 78% yield). ^{1}H NMR (500 MHz, $CDCl_3$, 298K): δ 4.01 (s, 6H, Ar-OCH_3), 8.43 (s, 2H, Ar-*H*); ^{13}C NMR (125 MHz, $CDCl_3$, 298K): δ 53.6, 131.4, 135.3, 149.4, 164.1.

Synthesis of 4-bromo-2,6-pyridinedimethanol (3): 3.6 g (13.15 mmol) of dimethyl-4-bromopyridine-2,6-dicarboxylate was dissolved in 100 mL of anhydrous ethanol in a 250 mL round-bottom flask with continuous magnetic stirring. Sodium borohydride (2.99 g) was added to the reaction mixture. The reaction mixture was then refluxed at 80 °C for 20 hours. After cooling to room temperature, the solvent was evaporated under reduced pressure. The resulting crude residue was treated with 50 mL of saturated aqueous ammonium chloride (NH_4Cl) solution. The aqueous layer was subsequently extracted with ethyl acetate in four portions of 20 mL each. The combined organic extracts were dried over anhydrous magnesium sulfate ($MgSO_4$). The drying agent was removed by filtration, and the filtrate was concentrated under reduced pressure, yielding Compound 3 as a white solid (1.85 g, 65% yield). ^{1}H NMR (500 MHz, DMSO-d6, 298K): δ 4.53 (s, 4H, Ar-OCH_2), 7.52(s, 2H, Ar-*H*); ^{13}C NMR (125 MHz, $CDCl_3$, 298K): δ 64.1, 121.7, 133.8, 163.7.

Synthesis of 4-bromopyridine-2,6-dicarbaldehyde (4): To a 250 mL round-bottom flask, 4-bromo-2,6-pyridinedimethanol (1.25 g, 5.75 mmol) and selenium dioxide (0.638 g, 5.75 mmol) were added in 1,4-dioxane (40 mL) and water (2 mL). The reaction mixture was refluxed at 100 °C with continuous stirring for 20 h. Upon completion, the hot reaction

mixture was filtered through a celite filter and the filter cake was washed with CH_2Cl_2. The combined filtrates were concentrated in vacuo. The crude product was purified by column chromatography on silica gel using CH_2Cl_2/hexane (5:1 v/v) as eluent to afford compound **4** as a pale pink solid (0.84 g, 69% yield). ^{1}H NMR (500 MHz, $CDCl_3$, 298K): δ 8.30 (s, 2H, Ar-*H*), 10.13 (s, 2H, Ar-C*HO*); ^{13}C NMR (125 MHz, $CDCl_3$, 298K): δ 128.6, 135.9, 154.0, 191.3.

Synthesis of 4-(pyren-2-yl)pyridine-2,6-dicarbaldehyde (PyDFP): In a three-neck round-bottom flask, 0.29 g (1.15 mmol) of pyrene-1-boronic acid, 0.25 g (1.15 mmol) of 4-bromopyridine-2,6-dicarbaldehyde, and 0.138 g (0.12 mmol, 10 mol%) of $Pd(PPh_3)_4$ were combined. The flask was evacuated and refilled with argon three times. A mixture of H_2O and THF (1:1 v/v, 10 mL) containing 20% aqueous Na_2CO_3 was then added to the reaction mixture. This mixture was refluxed at 80 °C for 20 hours. After the reaction was complete, the mixture was poured into 100 mL of ice-water. The resulting precipitate was collected by filtration and dried under vacuum. The crude product was purified using column chromatography on silica gel, with hexane/CH_2Cl_2 (1:4 v/v) as the eluent, yielding PyDFP as a yellow powder (0.262 g, 68% yield). ^{1}H NMR (500 MHz, $CDCl_3$, 298K): δ 8.02-8.36 (m, 9H, Ar-*H*), 8.52 (s, 2H, Ar-*H*), 10.37 (s, 2H, Ar-C*H*O); ^{13}C NMR (125 MHz, $CDCl_3$, 298K): δ 123.3, 124.7, 125.1, 125.2, 125.8, 126.2, 126.7, 127.1, 127.2, 127.5, 128.2, 129.0, 129.4, 130.7, 131.5, 132.2, 132.3, 152.5, 153.5, 192.7; MS (ESI-HRMS): m/z Calcd for $(C_{23}H_{14}NO_2)^+$: 336.1024 $(M+ H)^+$, found: 336.1026 $(M+ H)^+$.

Synthesis of PY-TTA COF: A 25 mL high-pressure flask was charged with 4,4′,4″-(1,3,5-triazine-2,4,6-triyl) trianiline (TTA; 21 mg, 0.06 mmol, 2 equiv) and 4-(pyren-2-yl)pyridine-2,6-dicarbaldehyde (PY; 30.1 mg, 0.09 mmol, 3 equiv). Anhydrous 1,4-dioxane (3.0 mL) was added, and the mixture was briefly sonicated for 1 min to ensure homogenization. An aqueous acetic acid solution (0.5 mL, 3.0 M) was then introduced, immediately producing a yellow precipitate. The resulting mixture was stirred under microwave irradiation of 2.45 GHz at 110 °C for 2 h and subsequently cooled to room temperature. The solid product was isolated by centrifugation, washed five times with anhydrous ethanol and twice with deionized water, and dried under vacuum at 120 °C overnight to afford the yellow PY-TTA COF.

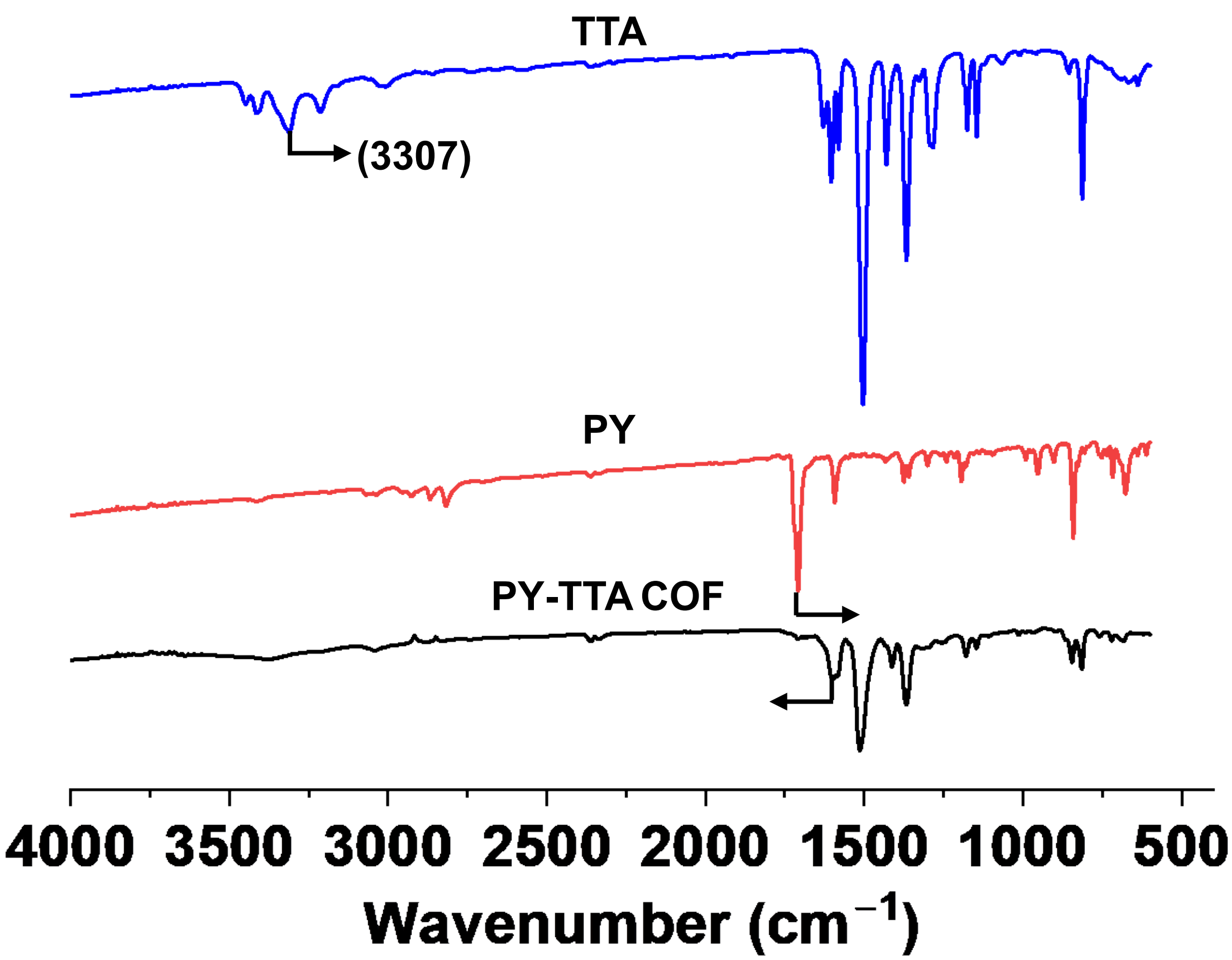

Figure S1. Stacked FT-IR spectra of 4,4',4''-(1,3,5-triazine-2,4,6-triyl) trianiline (TTA), 4-(pyren-2-yl) pyridine-2,6-dicarbaldehyde (Py), and PY-TTA COF.

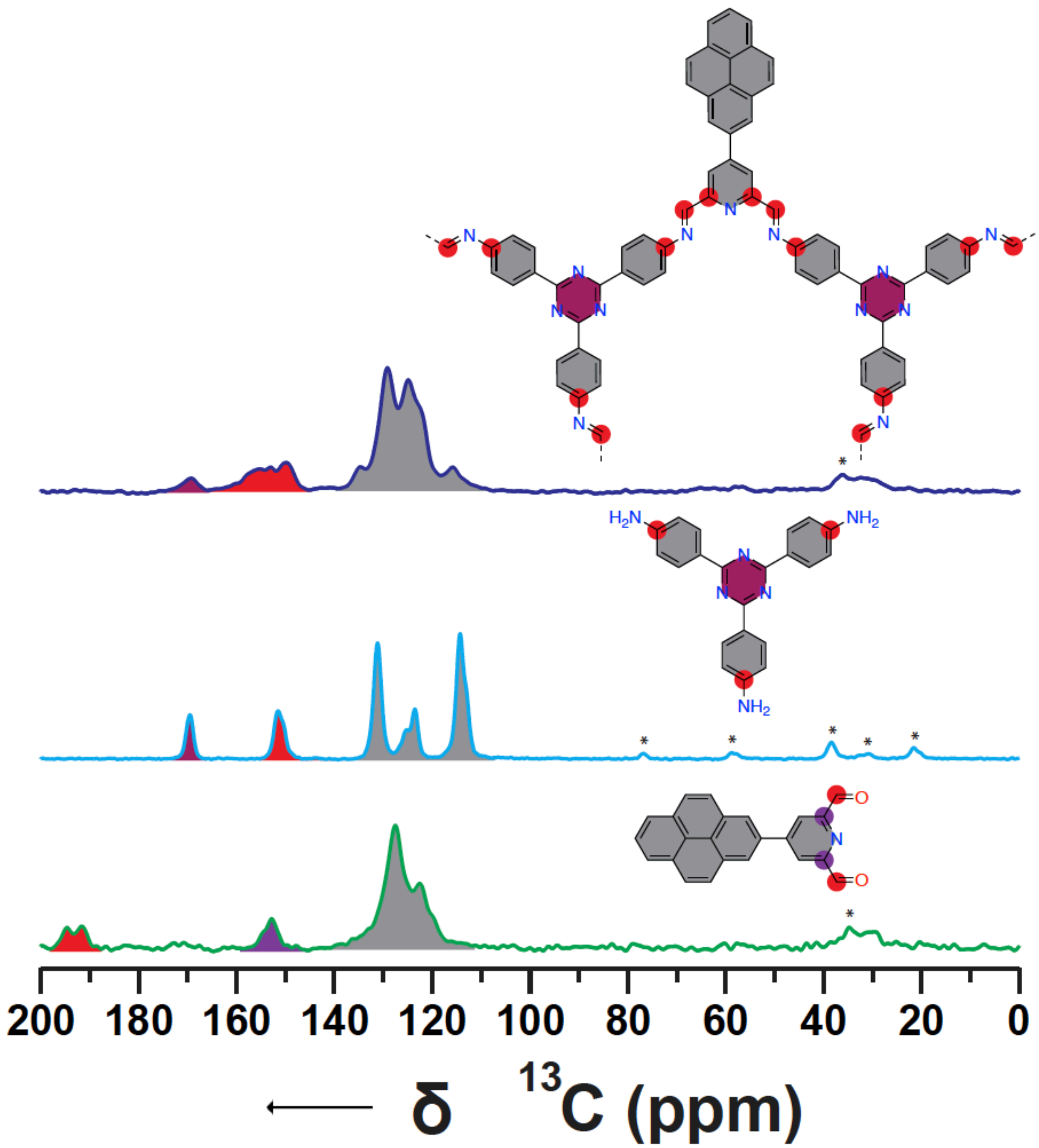

Figure S2. Stacked ¹³C CP/MAS solid-state NMR spectra of PY, TTA, and PY-TTA COF. Insets show the simplified chemical structures of the corresponding species. Asterisks (*) indicate spinning sidebands.

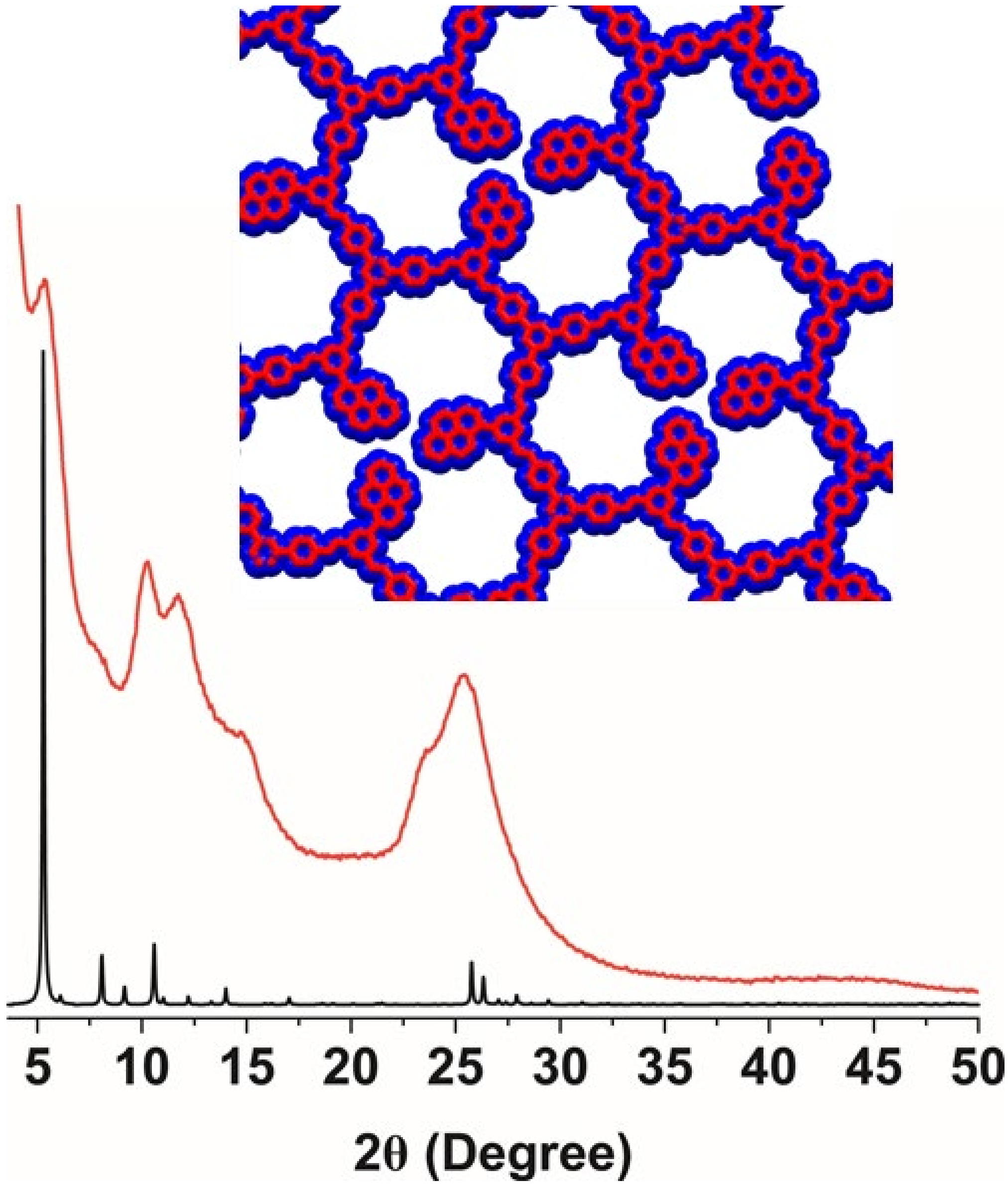

Figure S3. Experimental PXRD pattern of PY-TTA COF (red) and simulated PXRD pattern based on the proposed ABC-eclipsed stacking model (black). The simulated pattern reproduces the positions of the principal diffraction features observed experimentally.

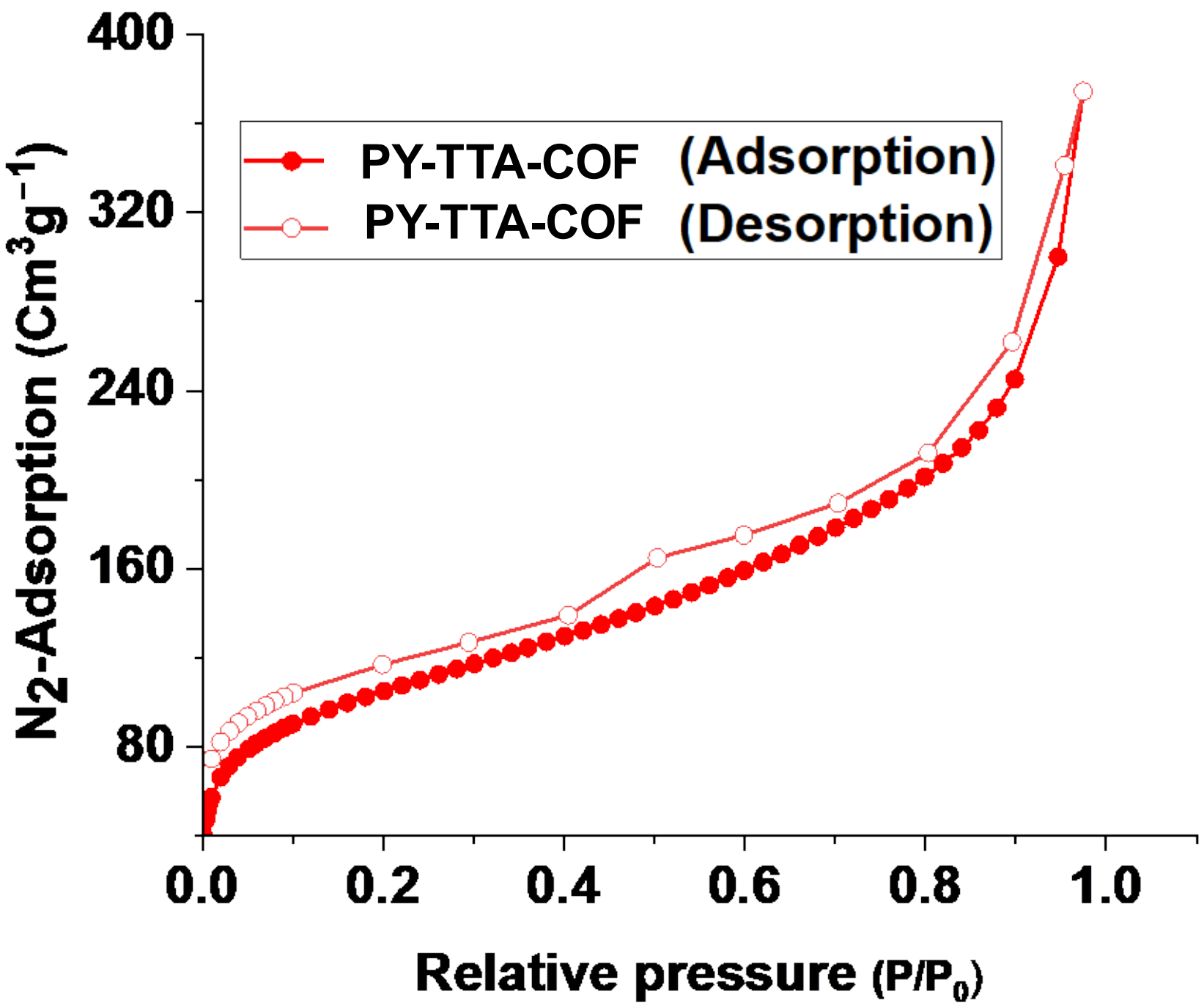

Figure S4. N_2 adsorption–desorption isotherms of PY-TTA COF measured at 77 K. BET analysis was performed after activation of the sample at 80 °C for 24 h. The calculated BET surface area is 373 $m^2\ g^{-1}$, consistent with the microporous nature of the framework.

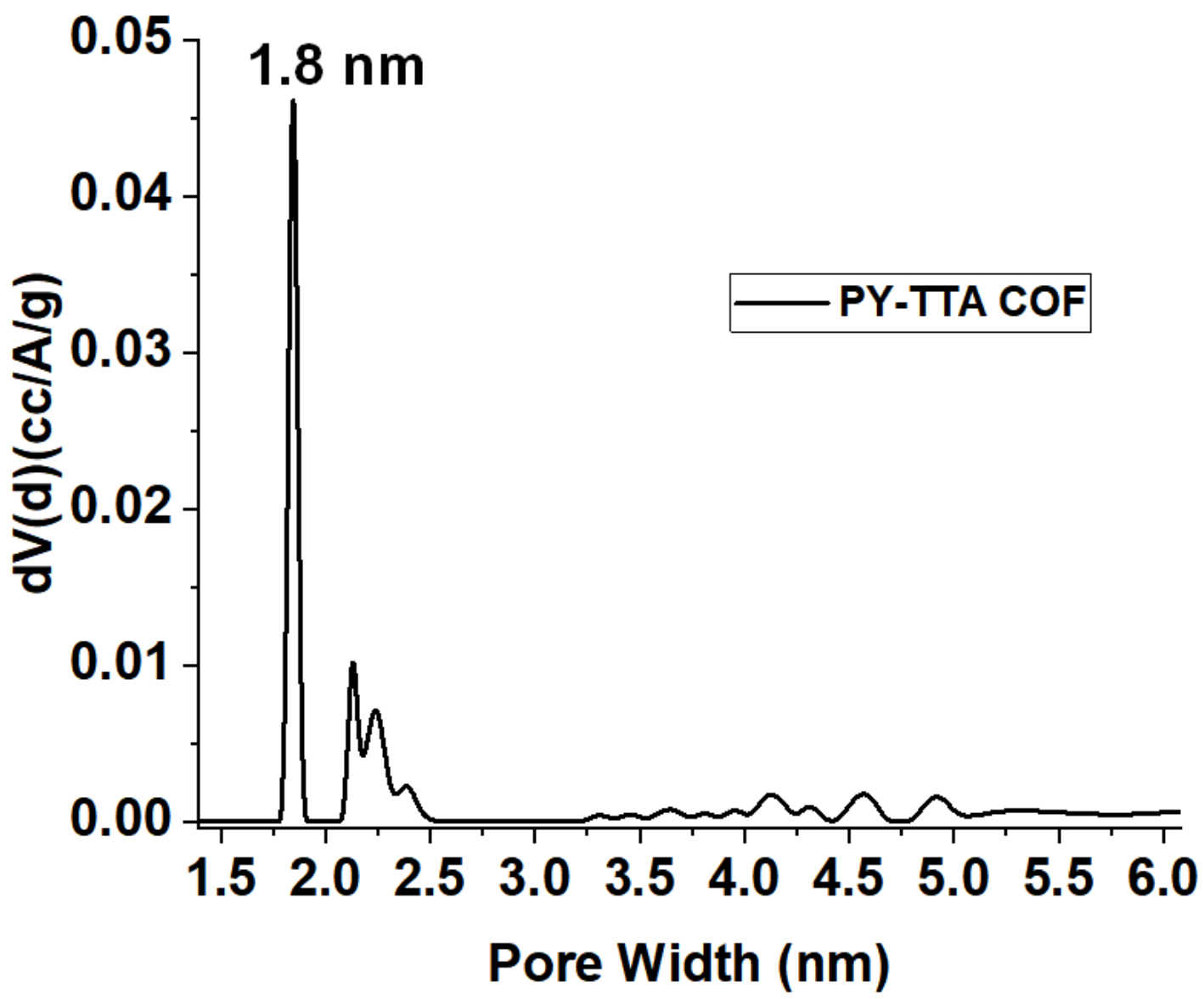

Figure S5. Non-Local Density functional theory (NLDFT)-derived pore size distribution of PY-TTA COF. The dominant pore width is centered at approximately 1.8 nm, in good agreement with the pore dimensions predicted from the structural model and consistent with the microporous nature of the framework.

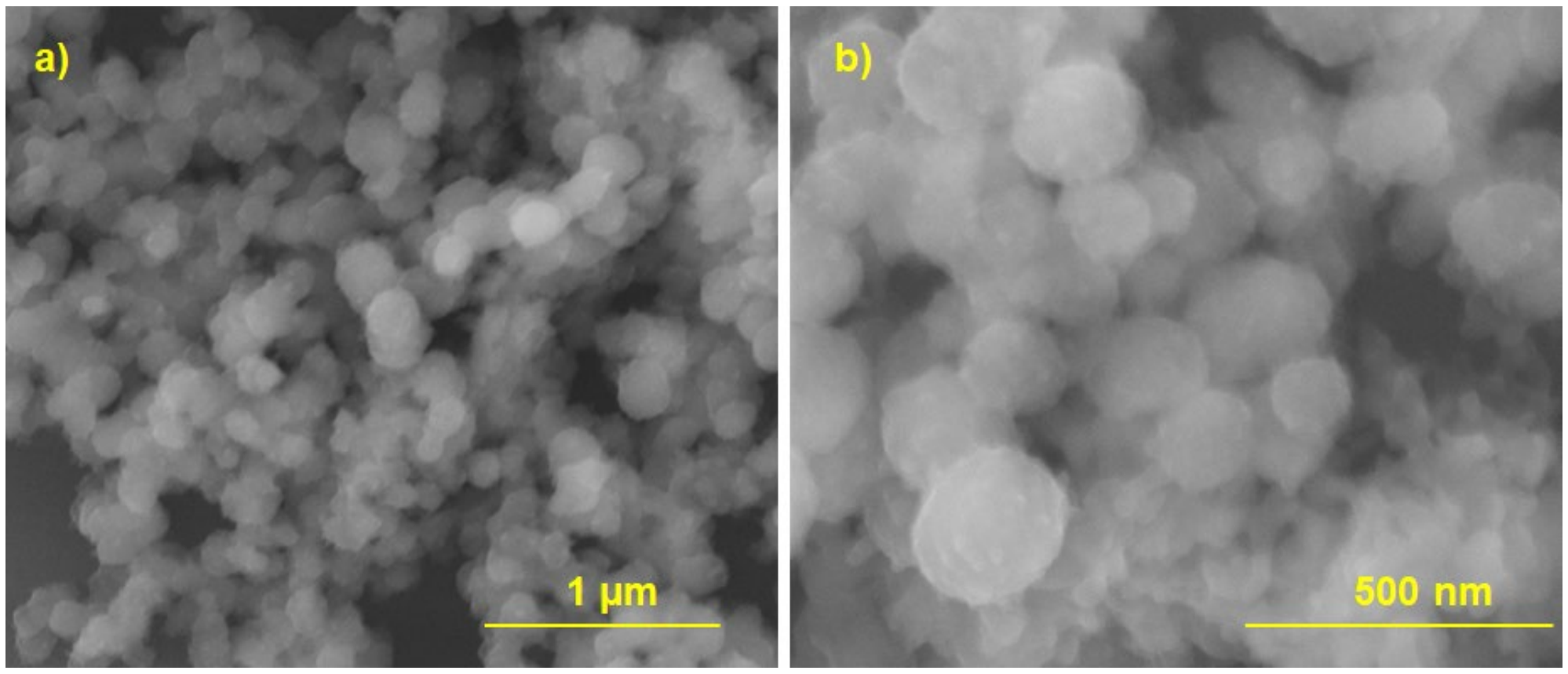

Figure S6. Scanning electron microscopy (SEM) images of PY-TTA COF recorded at different magnifications. (a) Low-magnification image showing aggregates of spherical particles. (b) Higher-magnification image highlighting the relatively uniform spherical morphology of the particles.

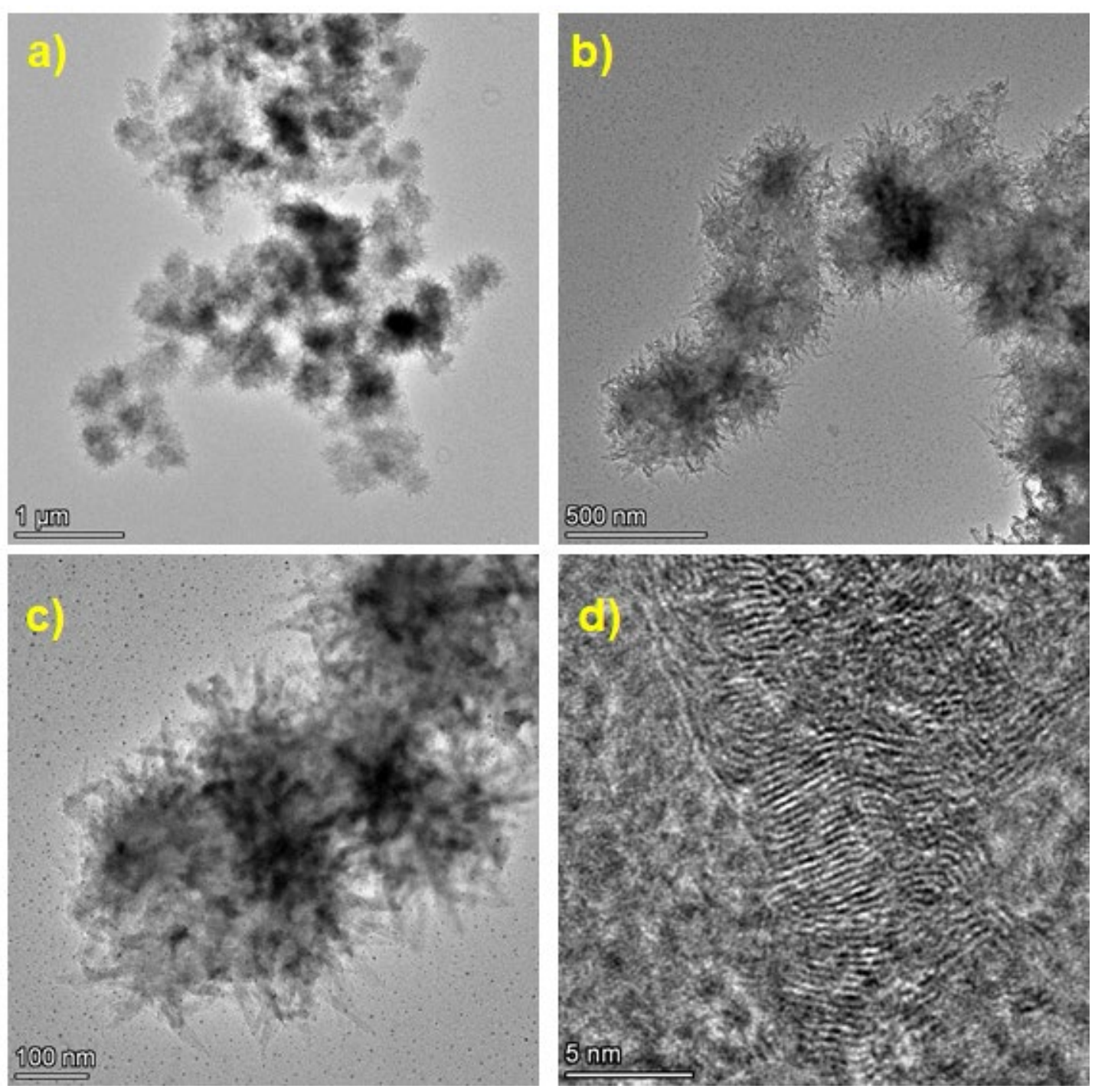

Figure S7. Transmission electron microscopy (TEM) and high-resolution TEM (HRTEM) images of PY-TTA COF at different magnifications. (a–c) TEM images showing aggregates of spherical particles with a textured, spiked surface morphology arising from radially oriented nanosheet-like features. (d) HRTEM image displaying well-defined lattice fringes, confirming the crystalline nature and long-range structural order of the PY-TTA COF.

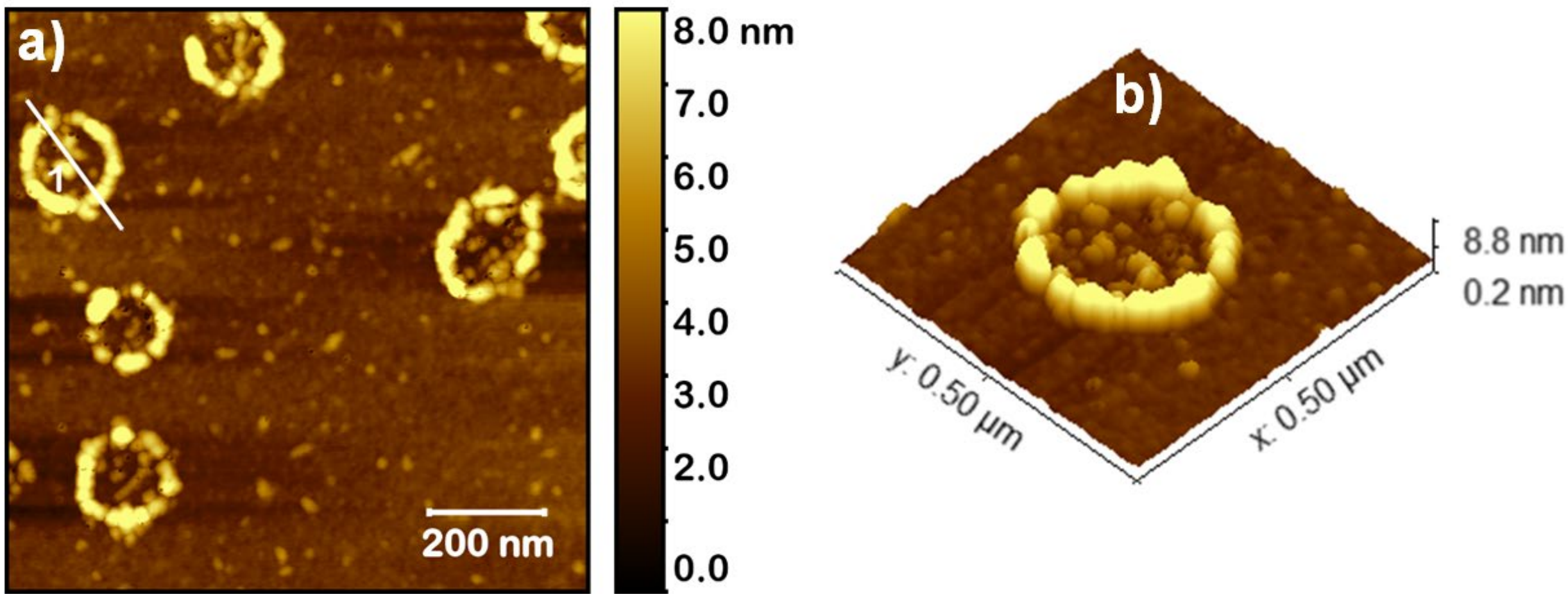

Figure S8. Tapping-mode atomic force microscopy (AFM) images of PY-TTA COF. (a) Topographic AFM image showing well-defined spherical particles with nanoscale surface protrusions, consistent with the textured morphology observed by TEM. (b) Three-dimensional AFM height rendering of an individual particle, highlighting its textured surface and nanoscale roughness.

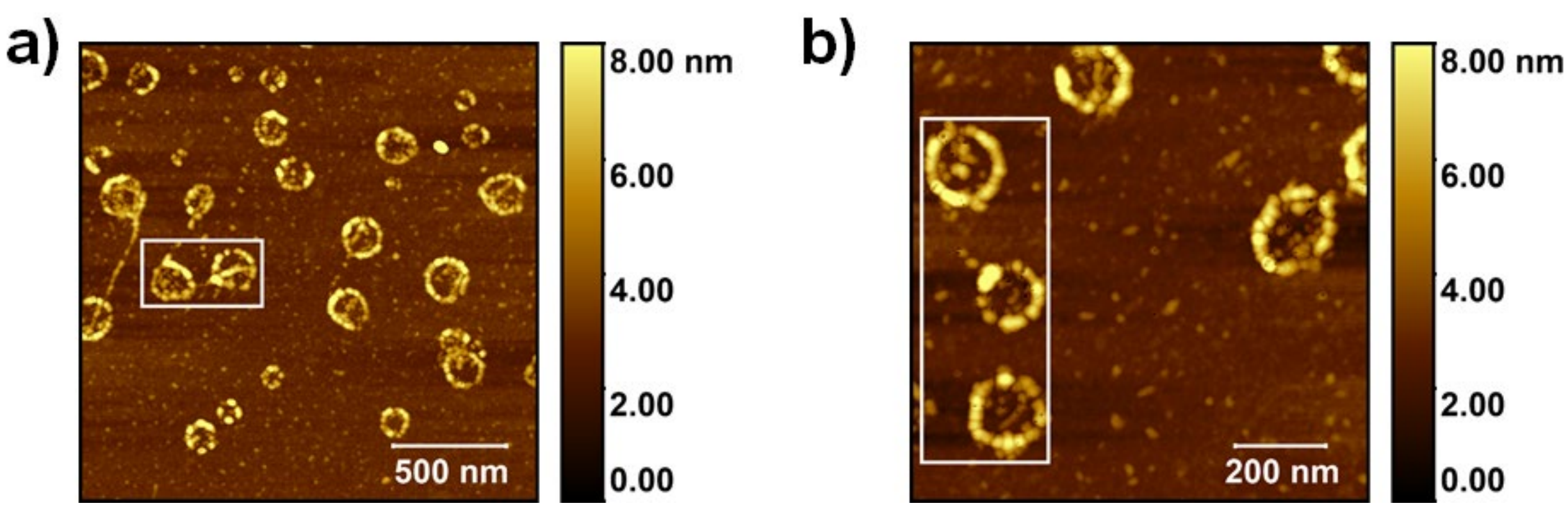

Figure S9. AFM topographic images of PY-TTA COF recorded at different regions of the sample surface. The measured mean surface roughness (Sa) ranges from 1.4 to 2.6 nm, consistent with the textured spiked morphology observed by AFM and TEM.

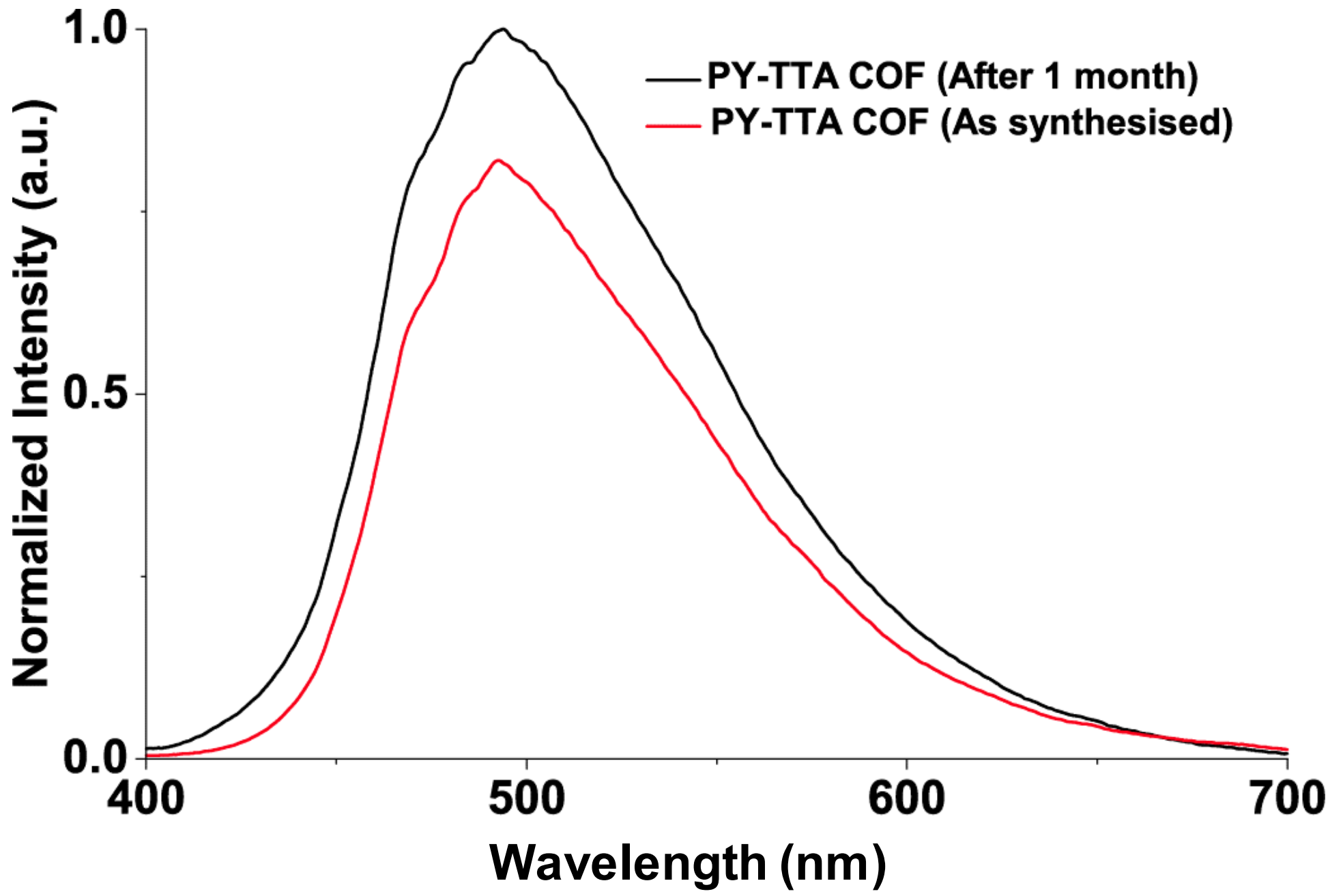

Figure S10. Comparison of the fluorescence spectra of the as-synthesized PY-TTA COF (red) and PY-TTA COF after one month of storage in aqueous suspension (black). Although modest variations in emission intensity are observed, the overall spectral profile is retained, indicating good fluorescence stability of the PY-TTA COF suspension over time.

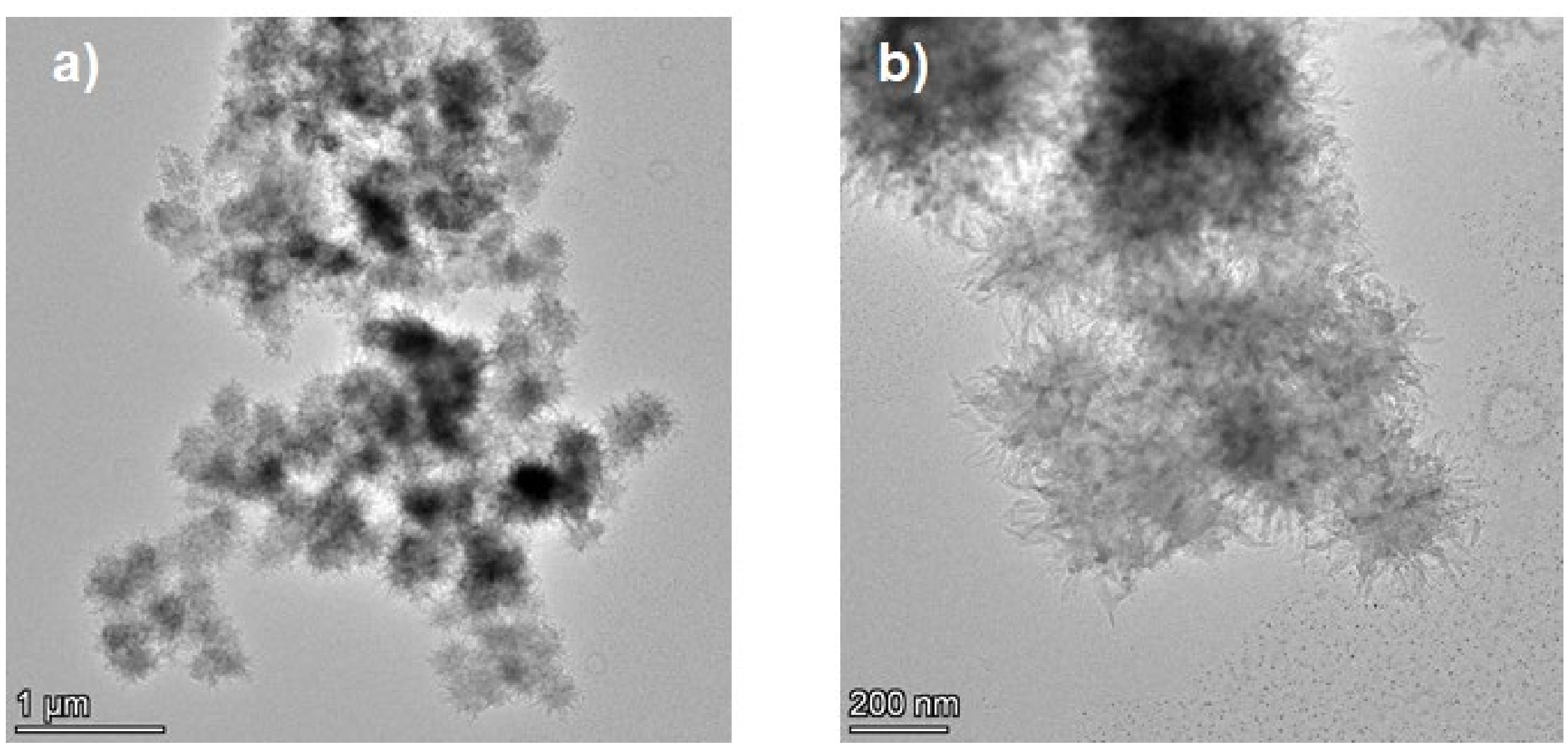

Figure S11. Comparison of HRTEM images of PY-TTA COF recorded immediately after preparation (a) and after prolonged standing in aqueous suspension (b). The characteristic spiked spherical morphology is retained after storage, indicating good morphological stability of the PY-TTA COF suspension over time.

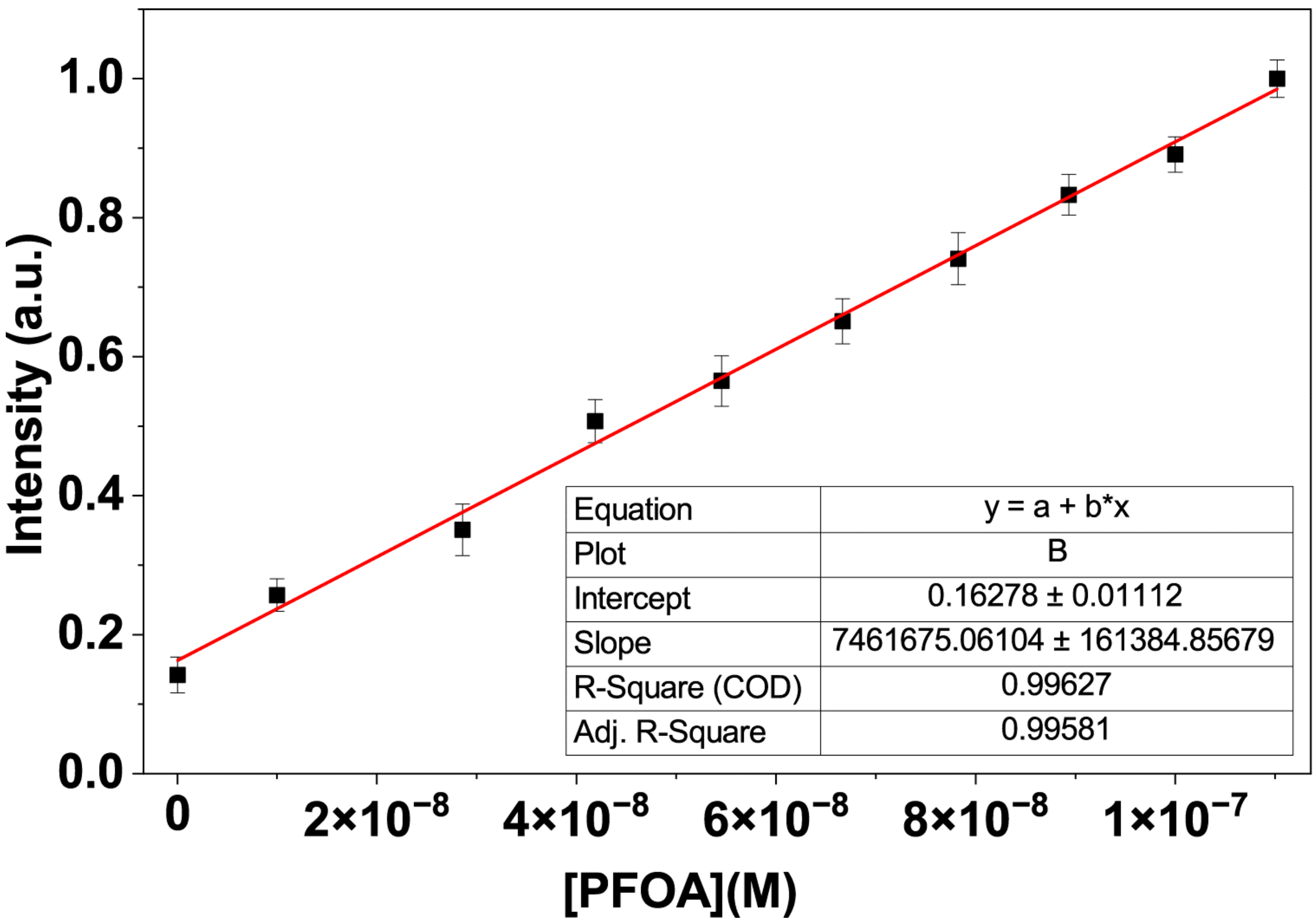

Figure S12. Calibration plot showing the relationship between fluorescence intensity of PY-TTA COF (λmax = 492 nm) and PFOA concentration. Error bars represent the standard deviation obtained from independent replicate measurements. The red line corresponds to the linear regression fit (R^2 = 0.996). The calibration curve was used to determine the limit of detection (LOD) using the 3σ/slope method, yielding an LOD of 2.7 μg L^{-1}.

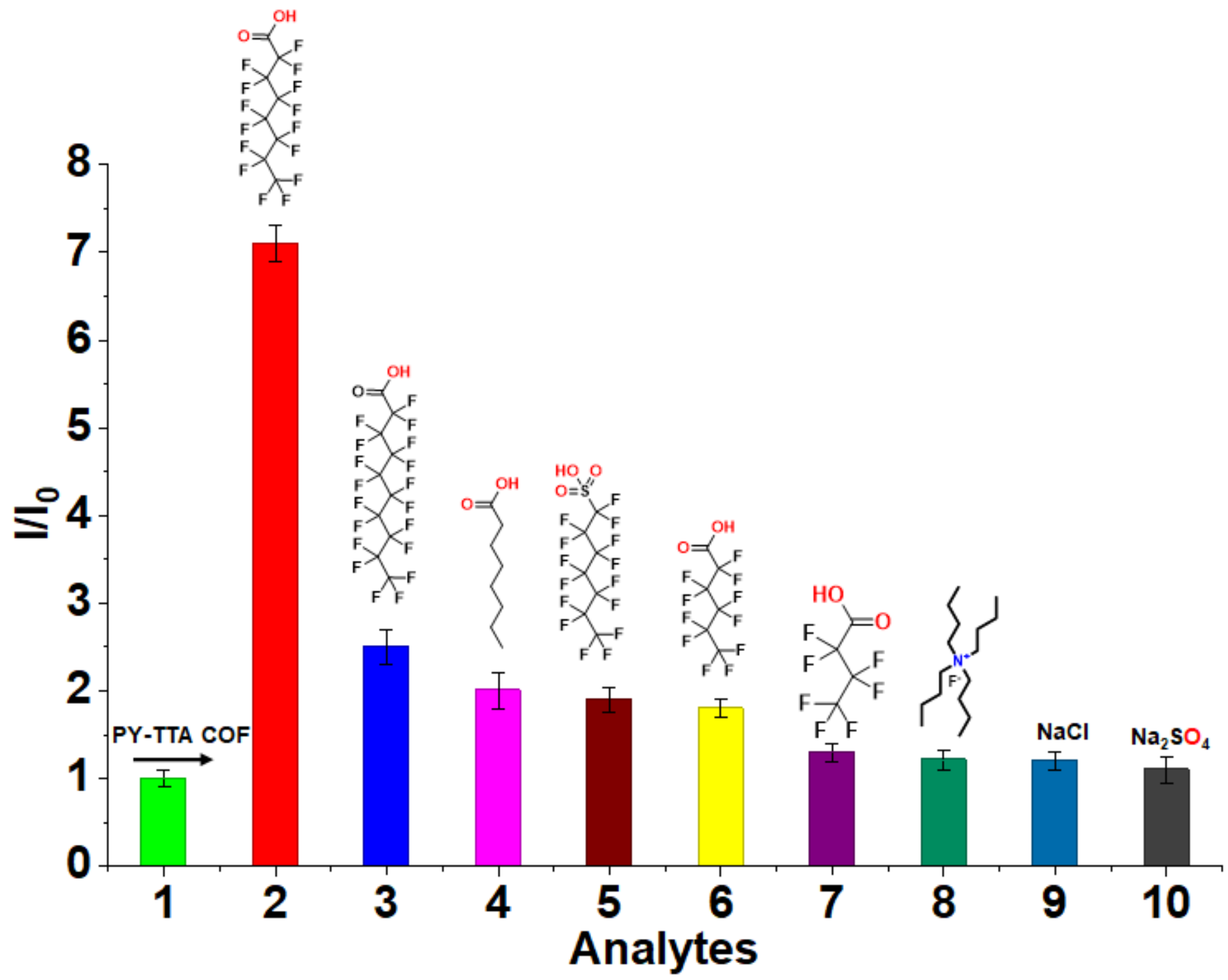

Figure S13. Relative fluorescence response (I/I_0) of PY-TTA COF toward PFOA and representative potential interferents, including PFDA, PFHA, PFOS, PFBA, octanoic acid (OA), tetrabutylammonium fluoride (TBAF), NaCl, and Na_2SO_4. Error bars represent the standard deviation obtained from independent measurements. PFOA produces the most pronounced fluorescence enhancement among the analytes examined, whereas the remaining PFAS analogues, organic compounds, and inorganic salts induce substantially weaker responses.

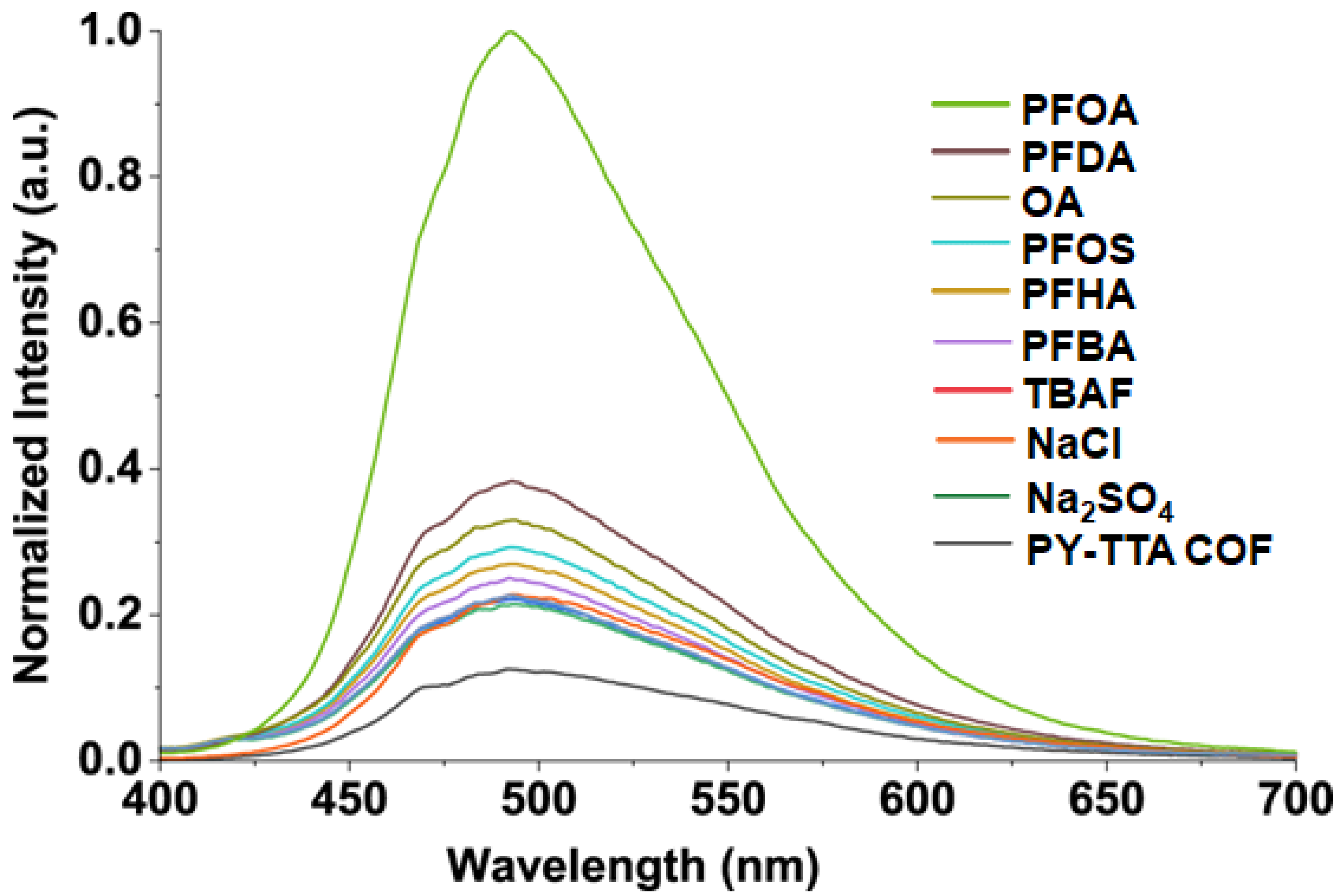

Figure S14. Fluorescence emission spectra of PY-TTA COF suspensions in the presence of PFOA, PFDA, PFHA, PFOS, PFBA, octanoic acid (OA), tetrabutylammonium fluoride (TBAF), NaCl, and Na_2SO_4. Among the analytes examined, PFOA produces the most pronounced fluorescence enhancement, whereas the remaining PFAS analogues, organic compounds, and inorganic salts induce substantially weaker responses, indicating preferential recognition of PFOA by the pyrene-functionalized COF.

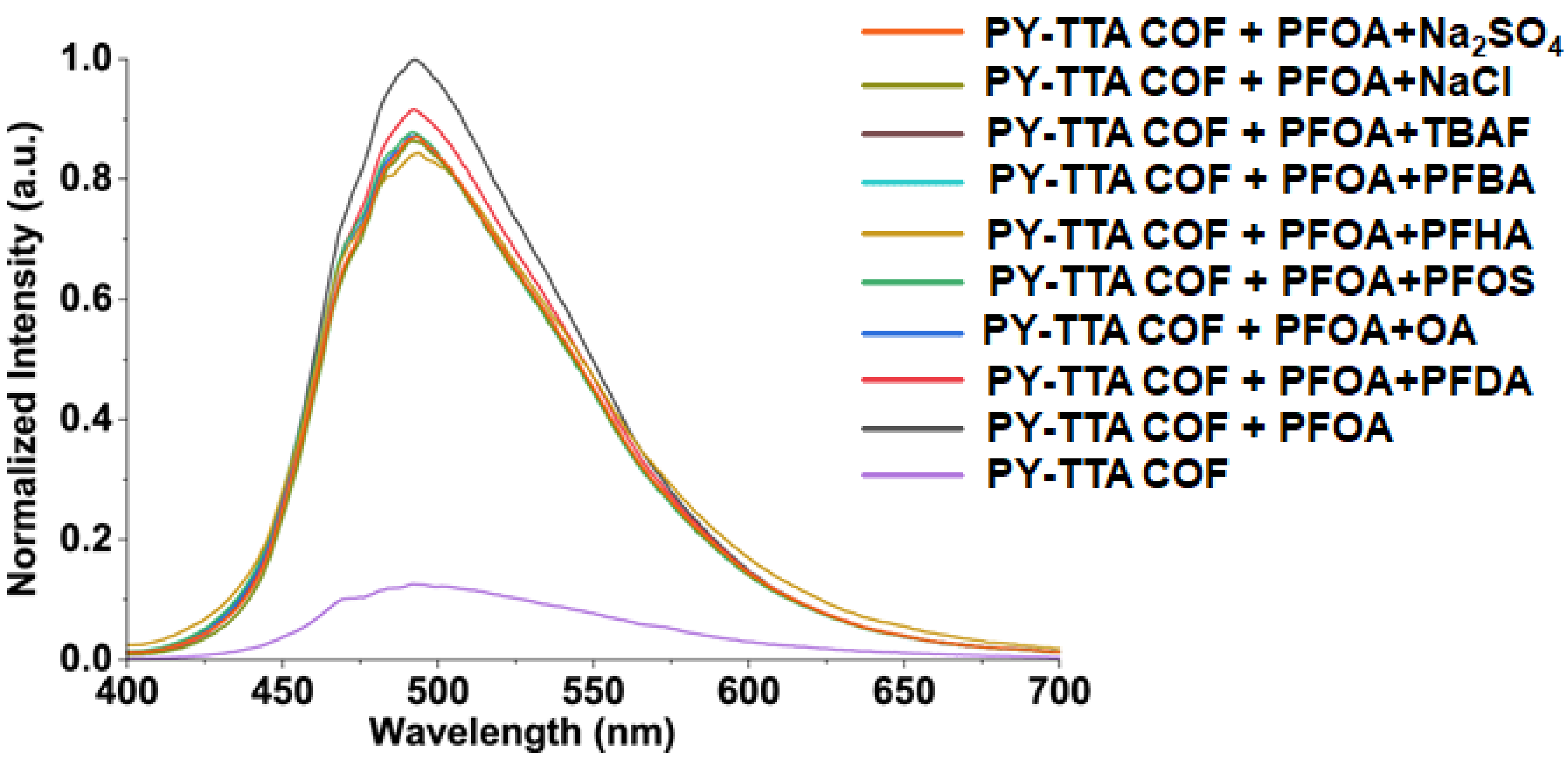

Figure S15. Fluorescence emission spectra of PY-TTA COF in the presence of PFOA (0.13 μM) and equimolar concentrations of competing analytes, including PFDA, PFHA, PFOS, PFBA, OA, TBAF, NaCl, and Na_2SO_4. The characteristic PFOA-induced fluorescence enhancement is largely retained in the presence of competing analytes, indicating minimal interference under the investigated conditions.

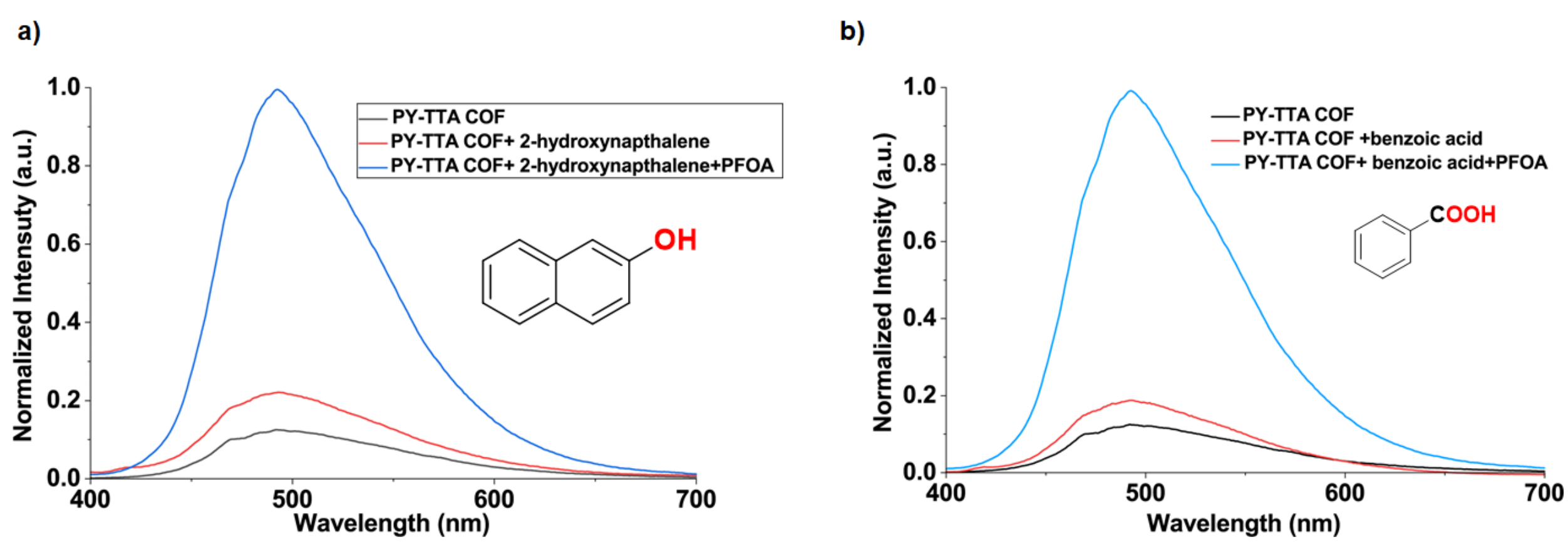

Figure S16. Fluorescence emission spectra of suspended PY-TTA COF in the absence and presence of 2-hydroxynaphthalene and benzoic acid upon subsequent addition of PFOA (0.13 μM).

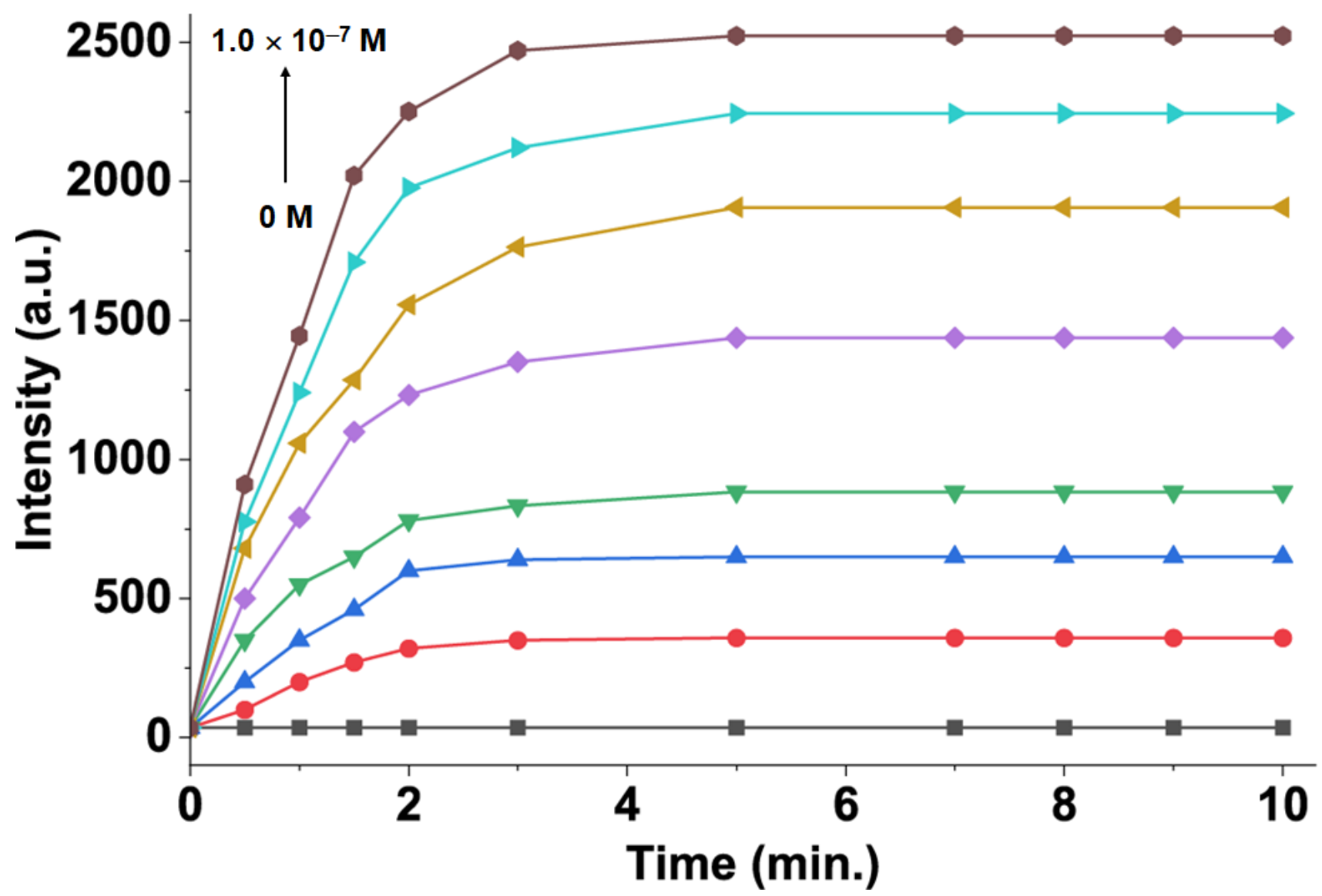

Figure S17. Time-dependent fluorescence response of PY-TTA COF following the addition of PFOA at different concentrations, showing rapid signal development and attainment of a stable fluorescence plateau within approximately 3 min.

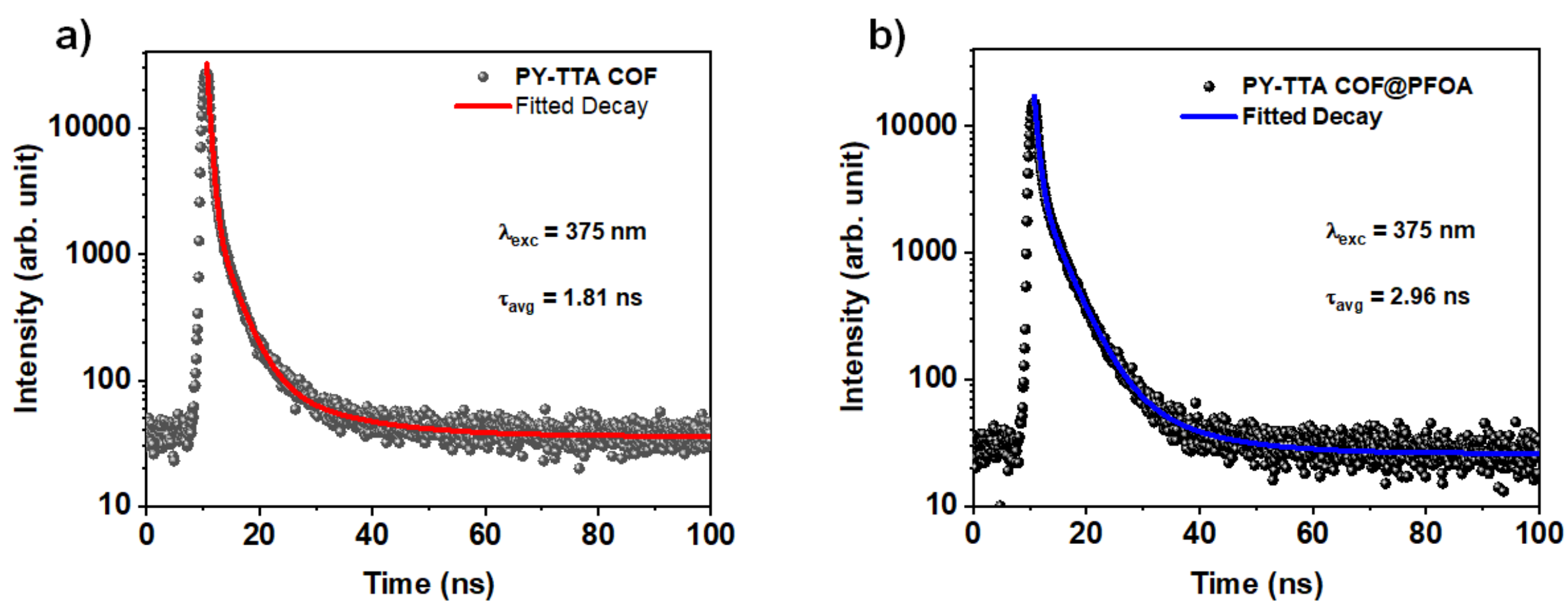

Figure S18. Time-resolved fluorescence decay profiles of (a) PY-TTA COF and (b) PY-TTA COF@PFOA recorded at λ_{ex} = 375 nm. The average fluorescence lifetime increases from 1.81 ns for PY-TTA COF to 2.96 ns upon PFOA incorporation, indicating a measurable change in the excited-state dynamics consistent with suppression of non-radiative relaxation pathways following host–guest interaction.

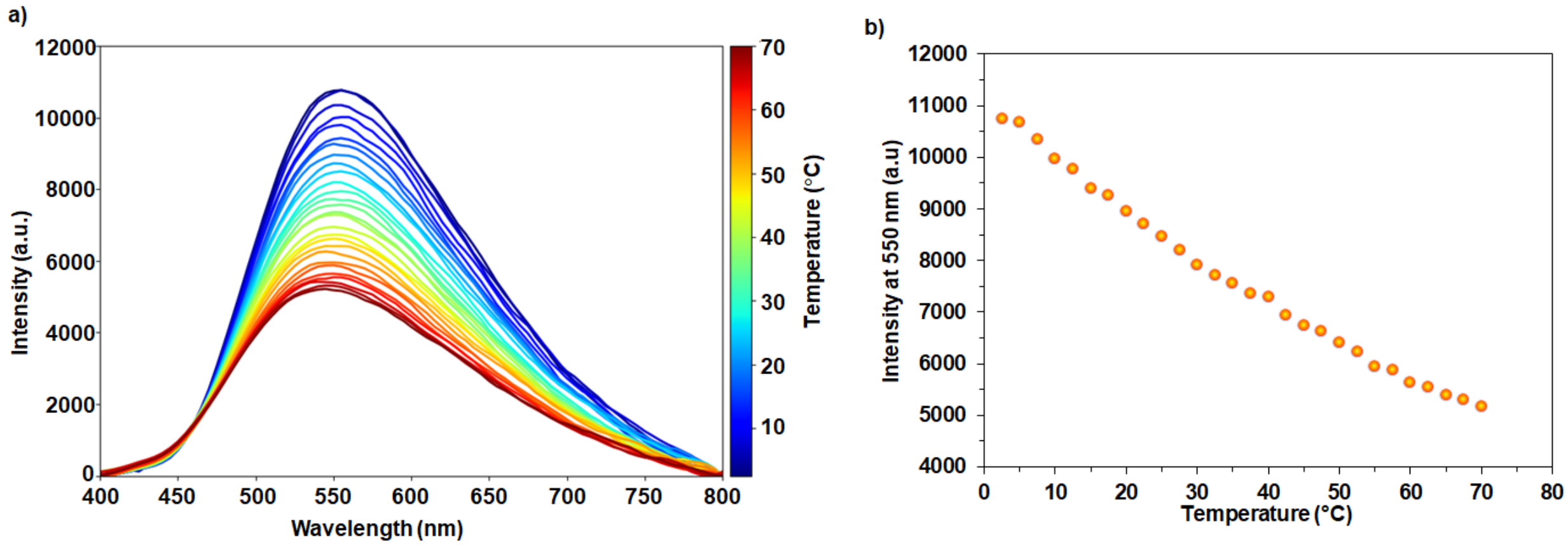

Figure S19. Variable-temperature fluorescence spectra of PY-TTA COF dispersed in water. (a) Emission spectra recorded between 400 and 800 nm over the temperature range of 2.5–70 °C under excitation at 340 nm. (b) Evolution of the emission intensity at 550 nm as a function of temperature. The fluorescence intensity increases progressively with decreasing temperature, consistent with reduced thermally activated molecular motion and suppression of non-radiative relaxation processes.

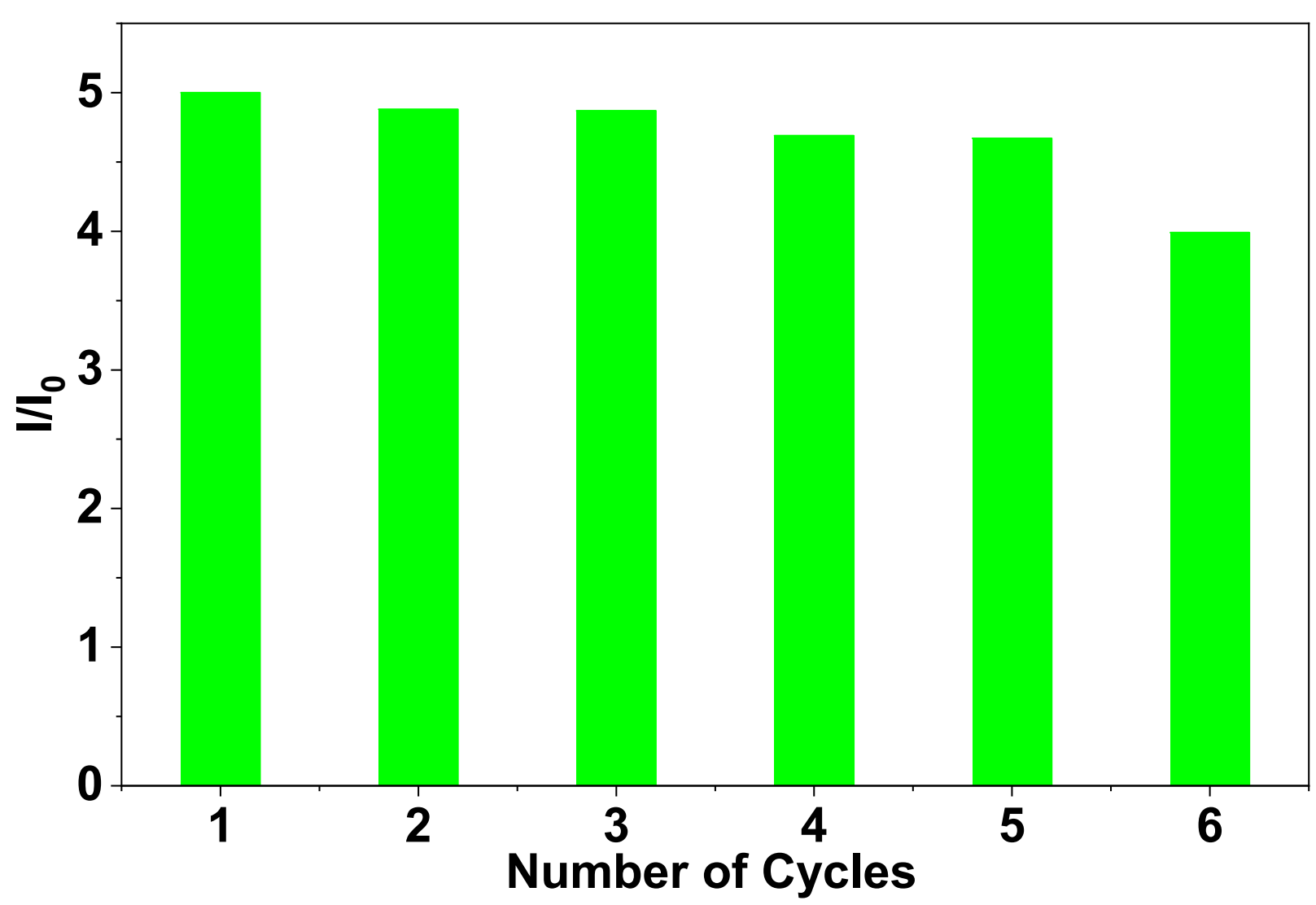

Figure S20. Recyclability of the PY-TTA COF fluorescence sensing platform over six consecutive PFOA sensing–regeneration cycles. The material retains a substantial fluorescence response over six cycles, with a moderate decrease in relative fluorescence intensity by the sixth cycle, demonstrating good reversibility and retention of sensing performance upon repeated use.

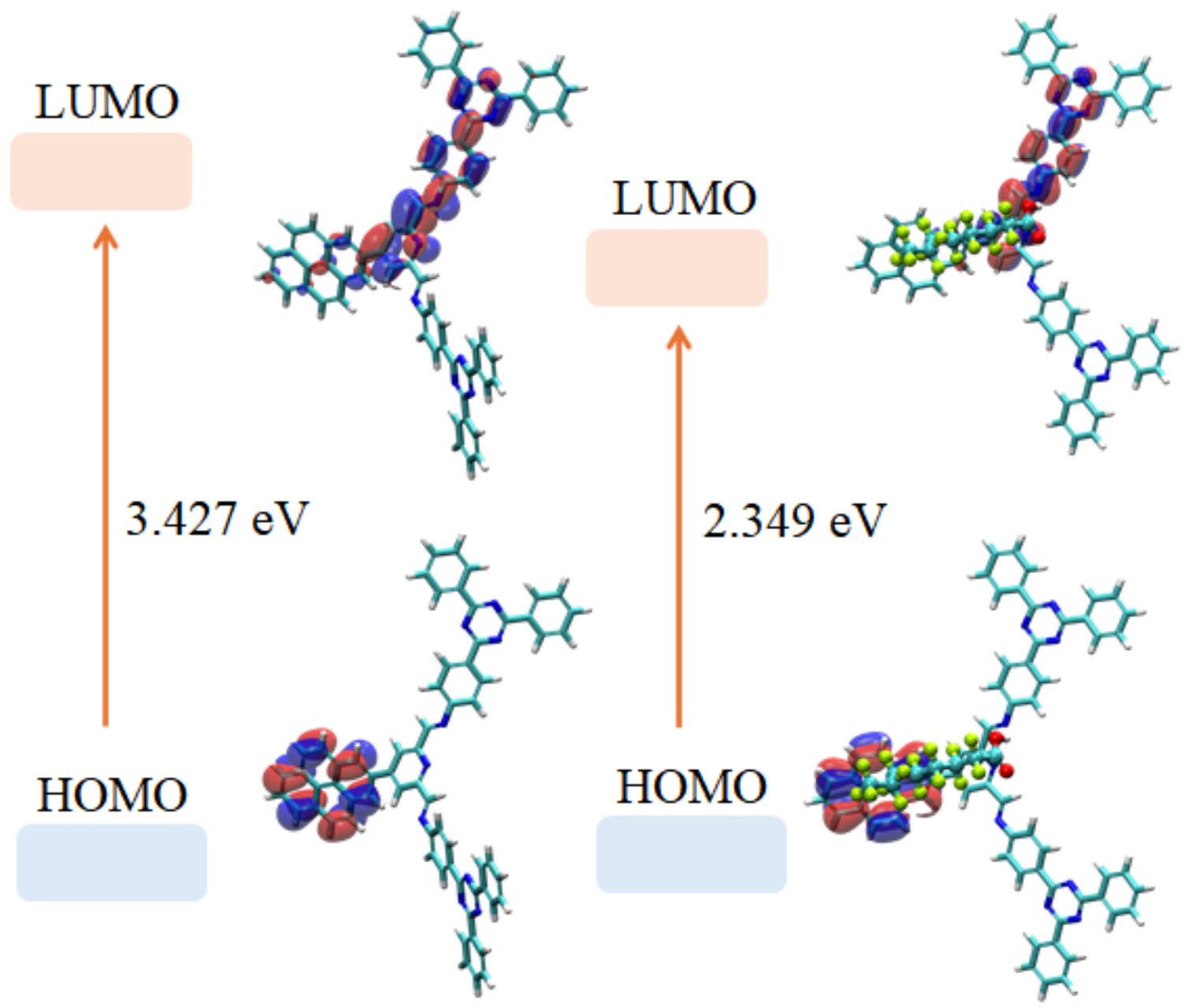

Figure S21. DFT-calculated HOMO–LUMO energy gaps and molecular orbital distributions for the pristine COF and the COF–PFOA complex. PFOA incorporation is associated with a reduction in the calculated HOMO–LUMO energy gap, consistent with guest-induced modification of the electronic structure and the experimentally observed red shift in the solid-state fluorescence emission.

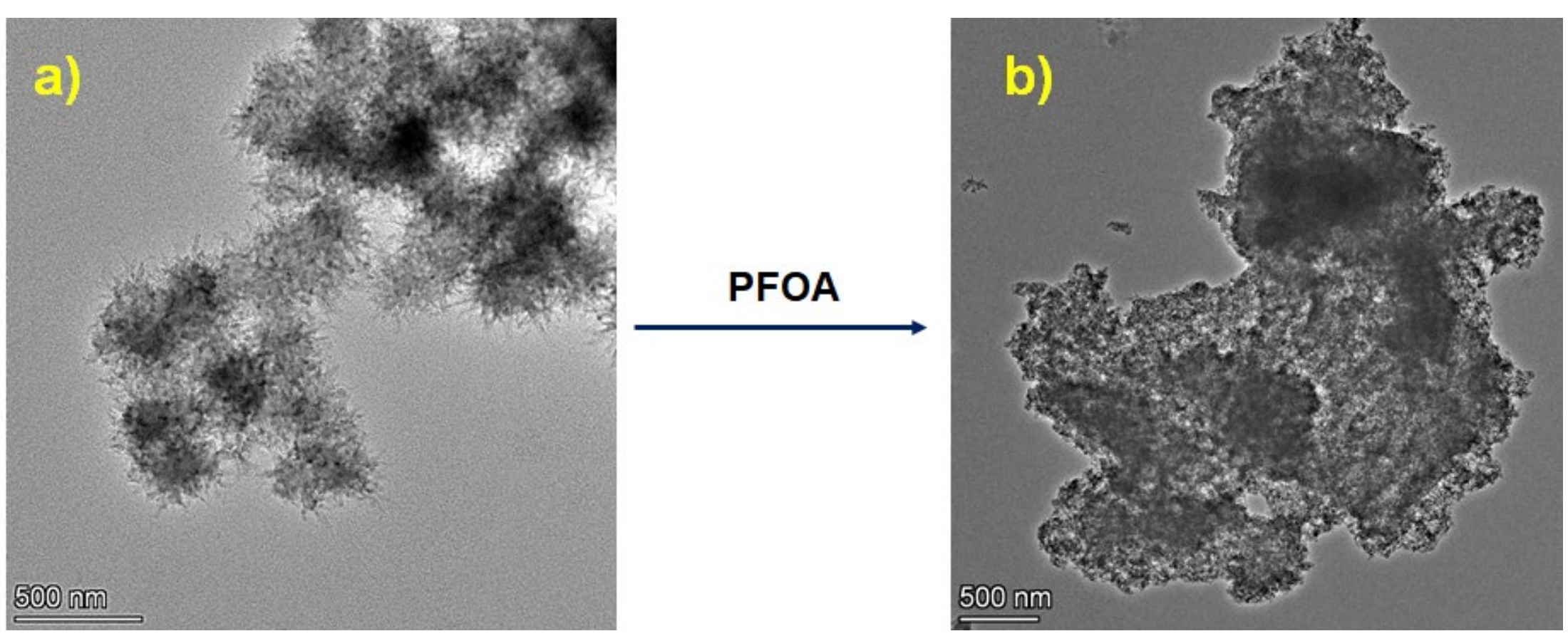

Figure S22. HRTEM images of PY-TTA COF before and after PFOA exposure. (a) Pristine PY-TTA COF showing well-defined spiked spherical particles. (b) PY-TTA COF after treatment with PFOA (PY-TTA COF@PFOA), displaying pronounced particle aggregation and reduced definition of the spiked surface morphology, consistent with guest-induced reorganization and increased interparticle association following PFOA incorporation. Scale bars: 500 nm.

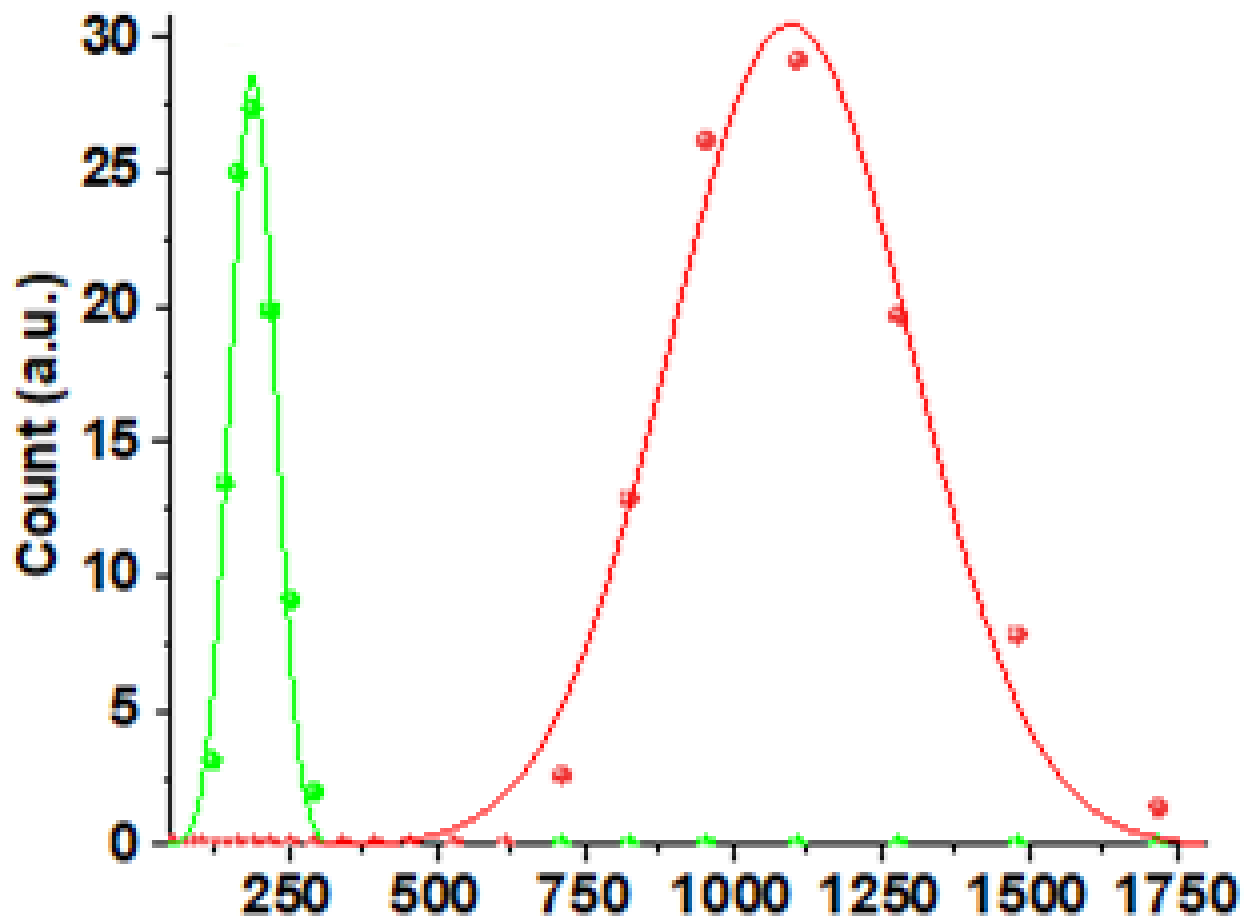

Figure S23. Dynamic light scattering (DLS) size distribution profiles of PY-TTA COF (green) and PY-TTA COF@PFOA (red). Upon PFOA incorporation, the hydrodynamic particle size increases substantially, consistent with enhanced particle aggregation. The corresponding zeta potentials (ζ = +6.44 ± 4 mV for PY-TTA COF and ζ = –26.7 ± 12 mV for PY-TTA COF@PFOA) are indicated above the distributions and reveal a pronounced shift toward negative surface charge following PFOA binding.

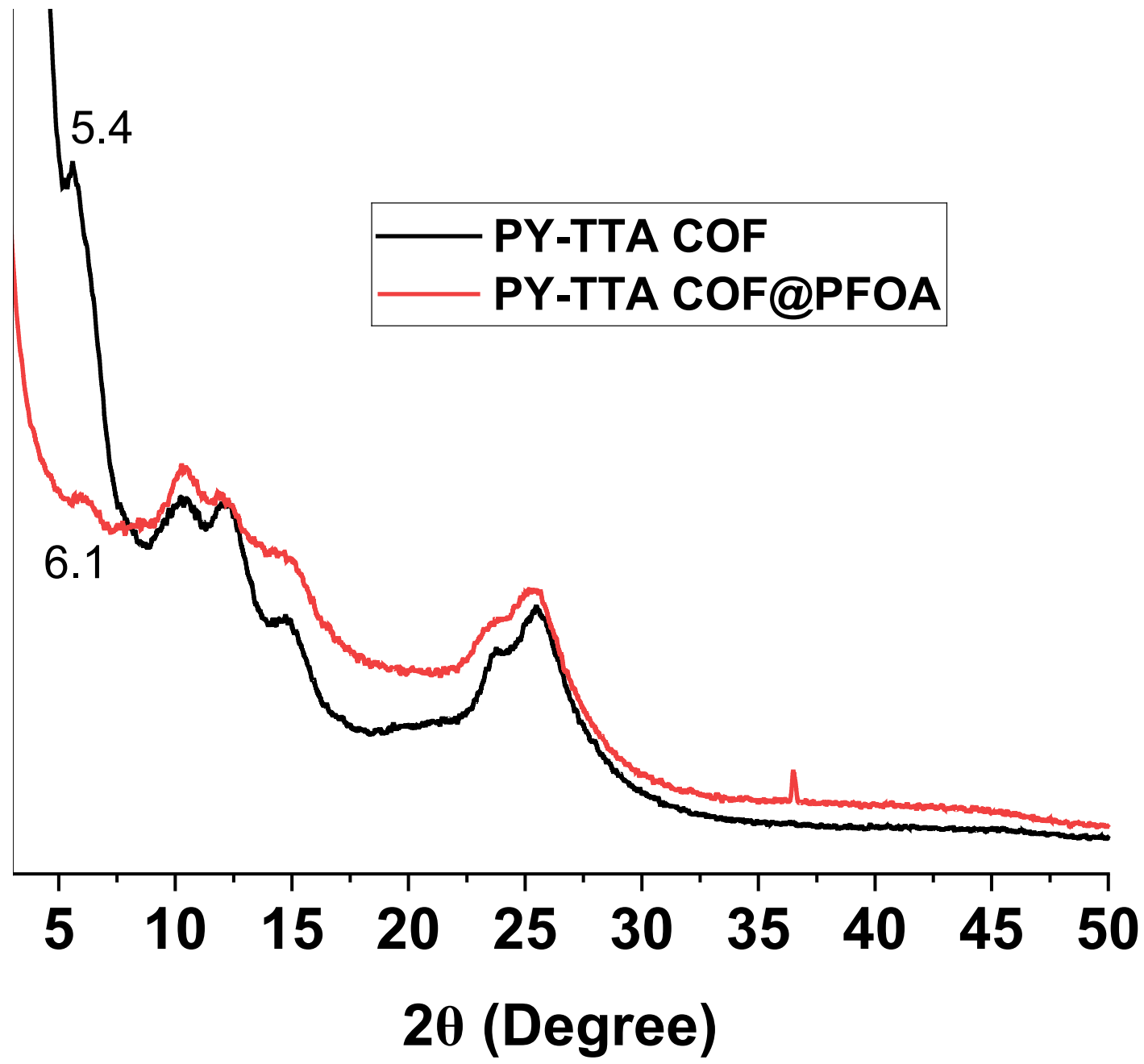

Figure S24. Powder X-ray diffraction (PXRD) patterns of pristine PY-TTA COF (black) and PFOA-loaded PY-TTA COF@PFOA (red). The reduction in peak intensity and a shift of the first diffraction peak toward higher 2θ values are consistent with guest-induced structural reorganization following PFOA incorporation. The preservation of the characteristic diffraction features confirms retention of framework crystallinity after guest uptake.

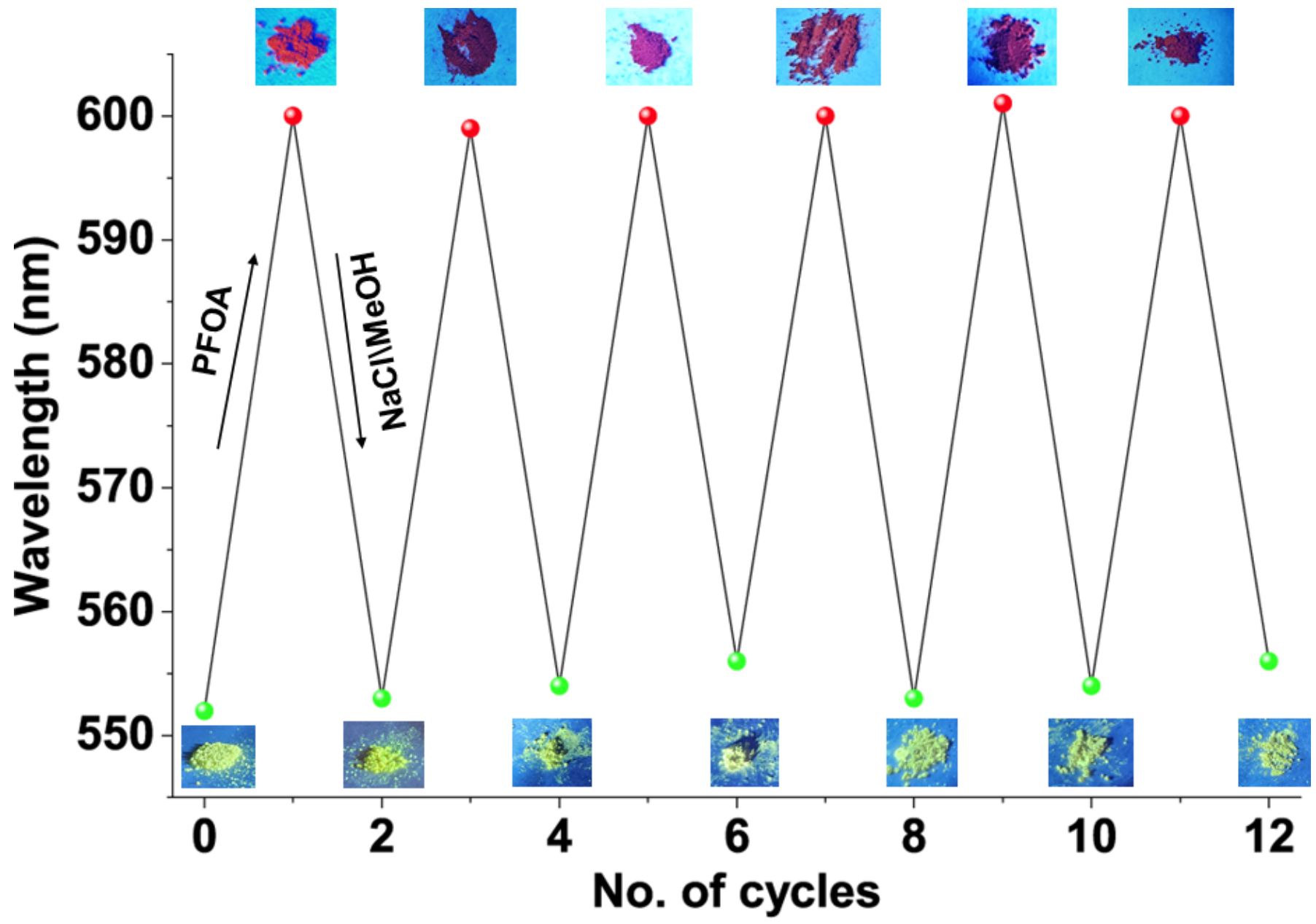

Figure S25. Reversible solid-state fluorescence switching of PY-TTA COF during six consecutive PFOA loading–regeneration cycles. Photographs and emission wavelength changes demonstrate repeated green-to-red fluorescence switching upon PFOA incorporation and recovery of the original emission state following regeneration with saturated NaCl/water/methanol solution.

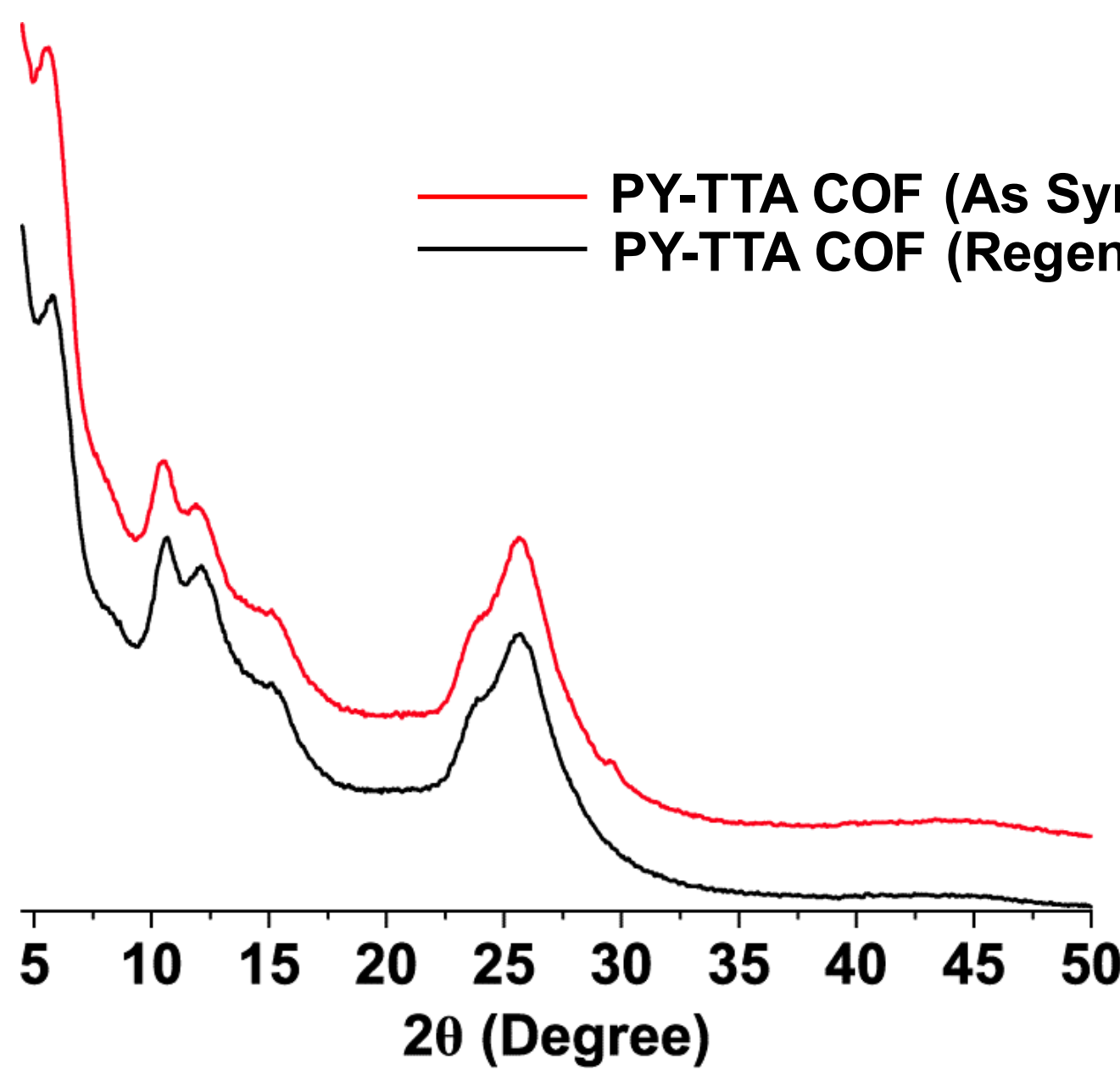

Figure S26. Powder X-ray diffraction (PXRD) patterns of pristine PY-TTA COF (red) and regenerated PY-TTA COF (black). The preservation of the principal diffraction features after regeneration indicates that the framework remains structurally intact and retains its crystallinity following repeated adsorption–desorption cycles.

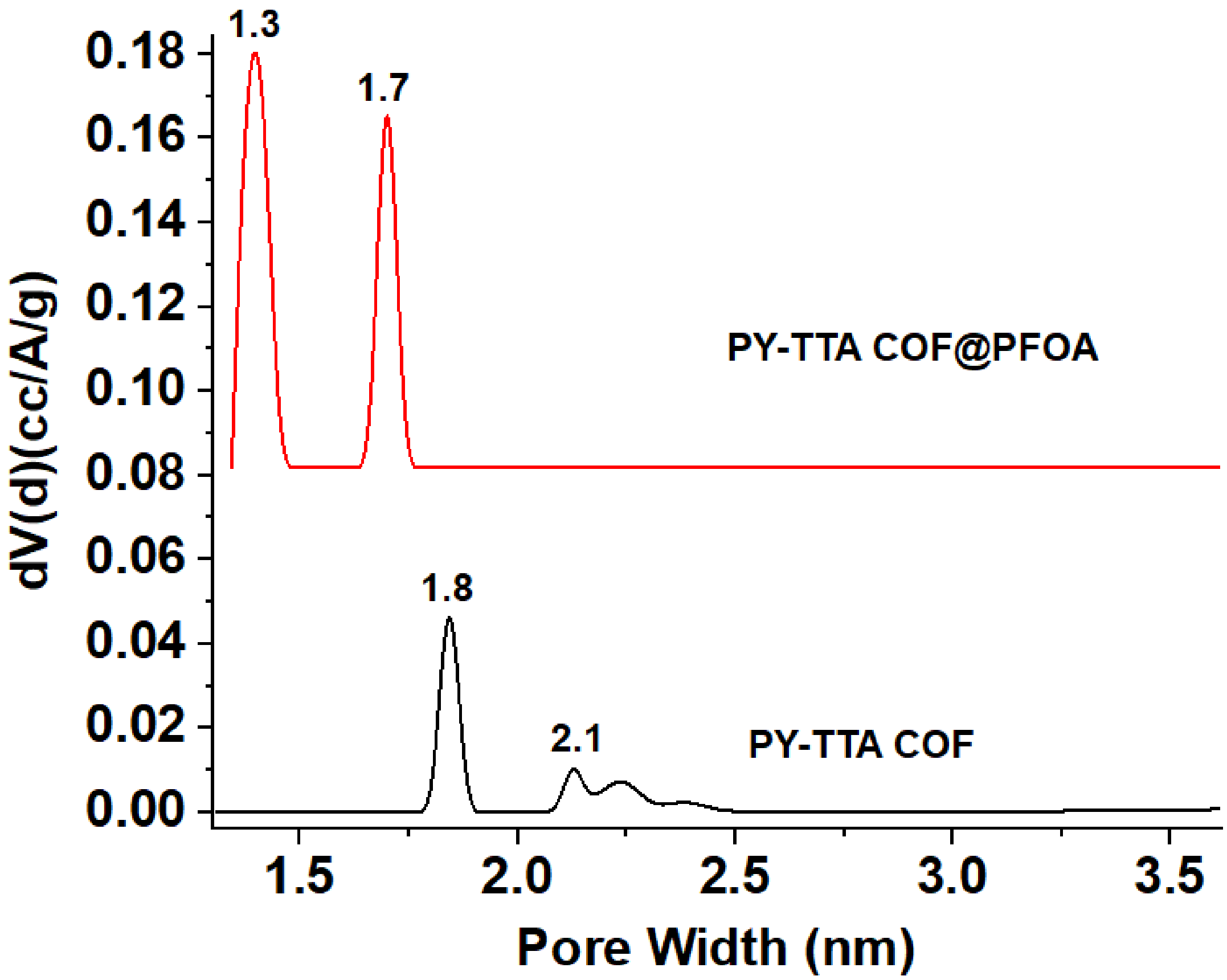

Figure S27. NLDFT-derived pore size distribution plots of PY-TTA COF (black) and PY-TTA COF@PFOA (red). The pristine PY-TTA COF exhibits a dominant pore width centered at ~1.8 nm, consistent with the pore dimensions predicted from the structural model. Upon PFOA incorporation, the pore size distribution shifts toward smaller effective pore widths (~1.3 nm), consistent with guest occupancy within the pore channels and modification of the accessible pore environment.

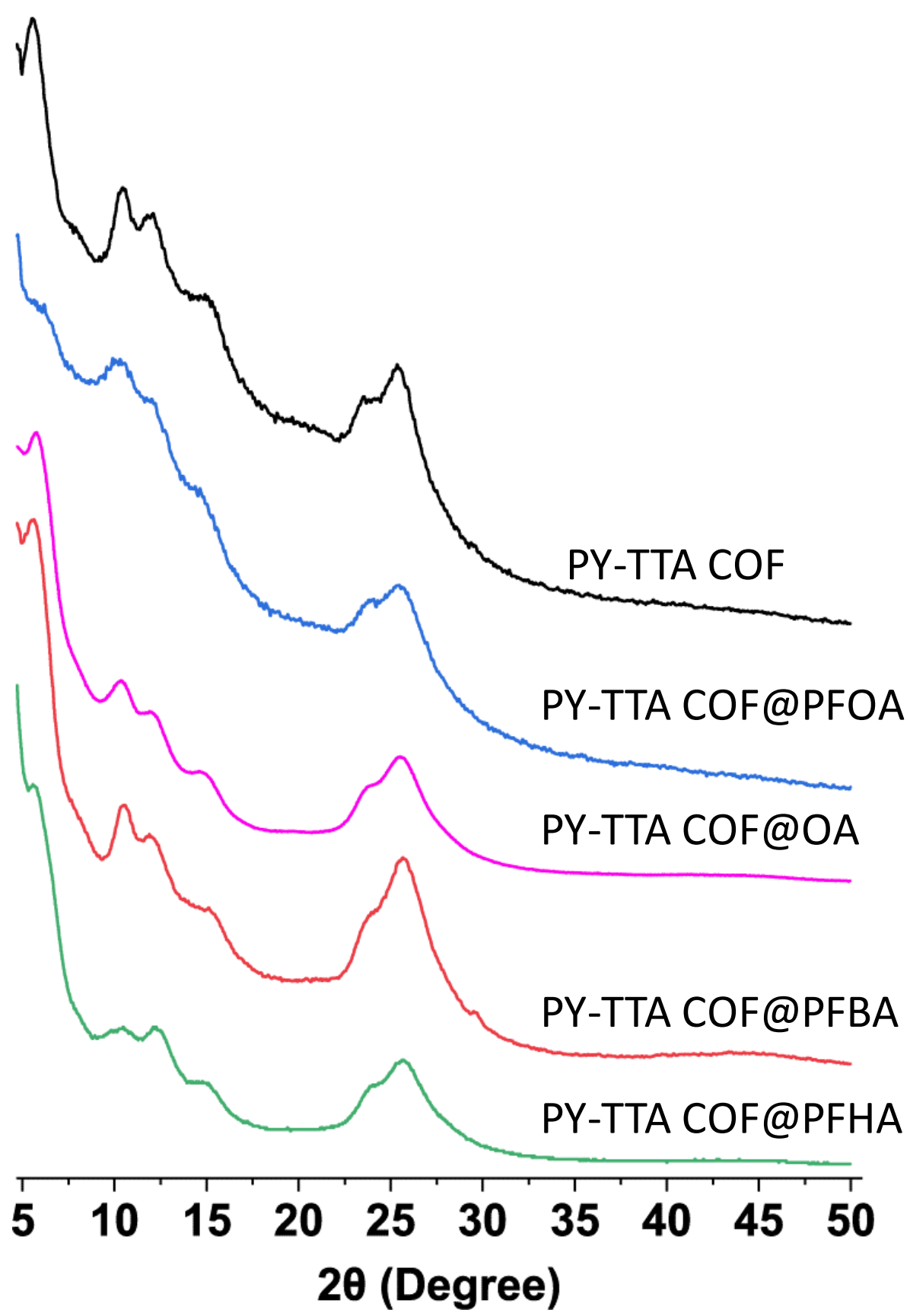

Figure S28. Powder X-ray diffraction (PXRD) patterns of PY-TTA COF and its guest-loaded analogues: PY-TTA COF@PFOA, PY-TTA COF@OA, PY-TTA COF@PFBA, and PY-TTA COF@PFHA. The characteristic diffraction features are retained across all samples, indicating preservation of the overall framework while allowing guest-dependent changes in the diffraction profiles.

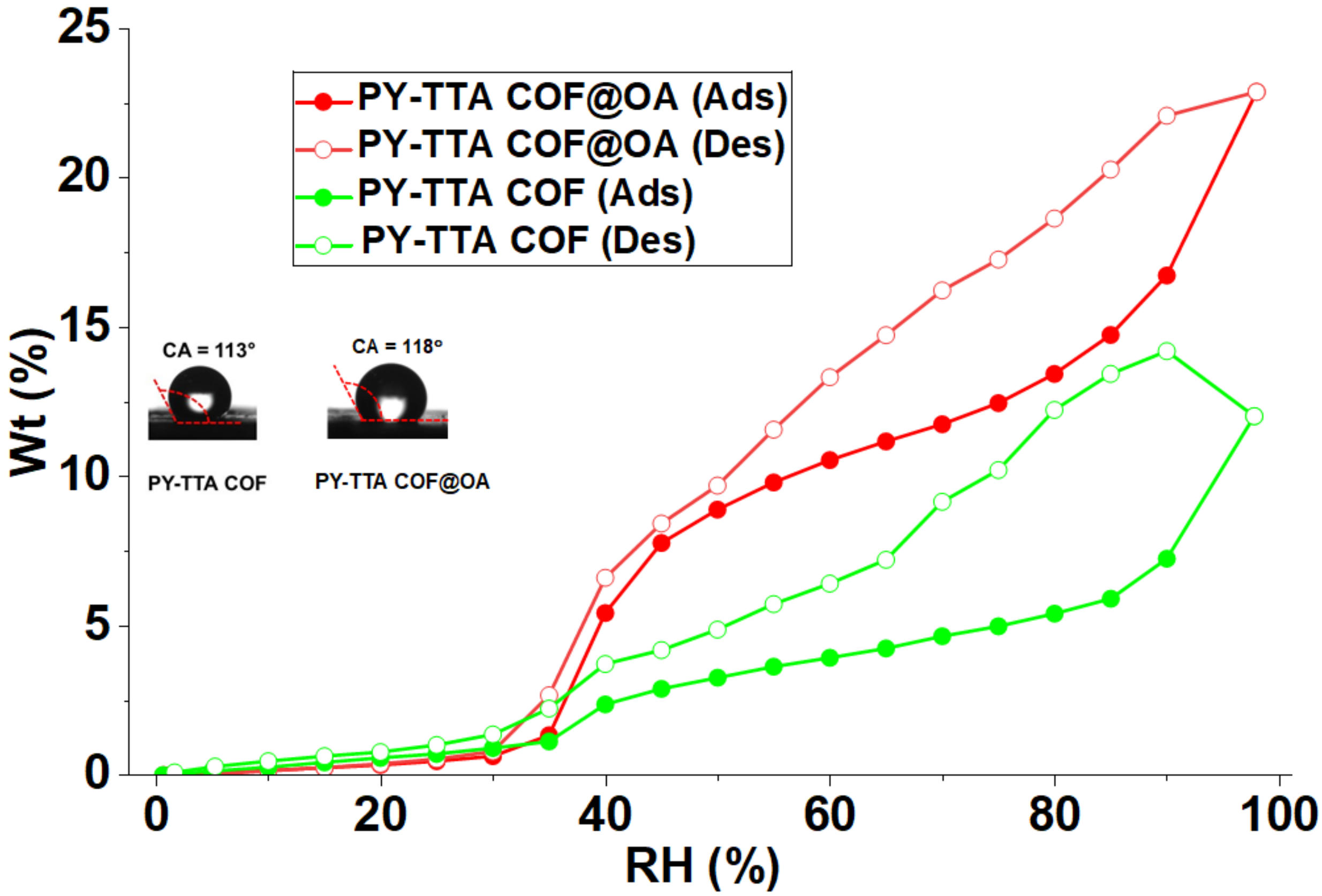

Figure S29. Water adsorption–desorption isotherms of PY-TTA COF@OA. The inset shows the corresponding water contact angle of the OA-treated COF. The sorption behavior and wettability remain comparable to those of the pristine PY-TTA COF, consistent with a comparatively weak perturbation of the COF by OA.

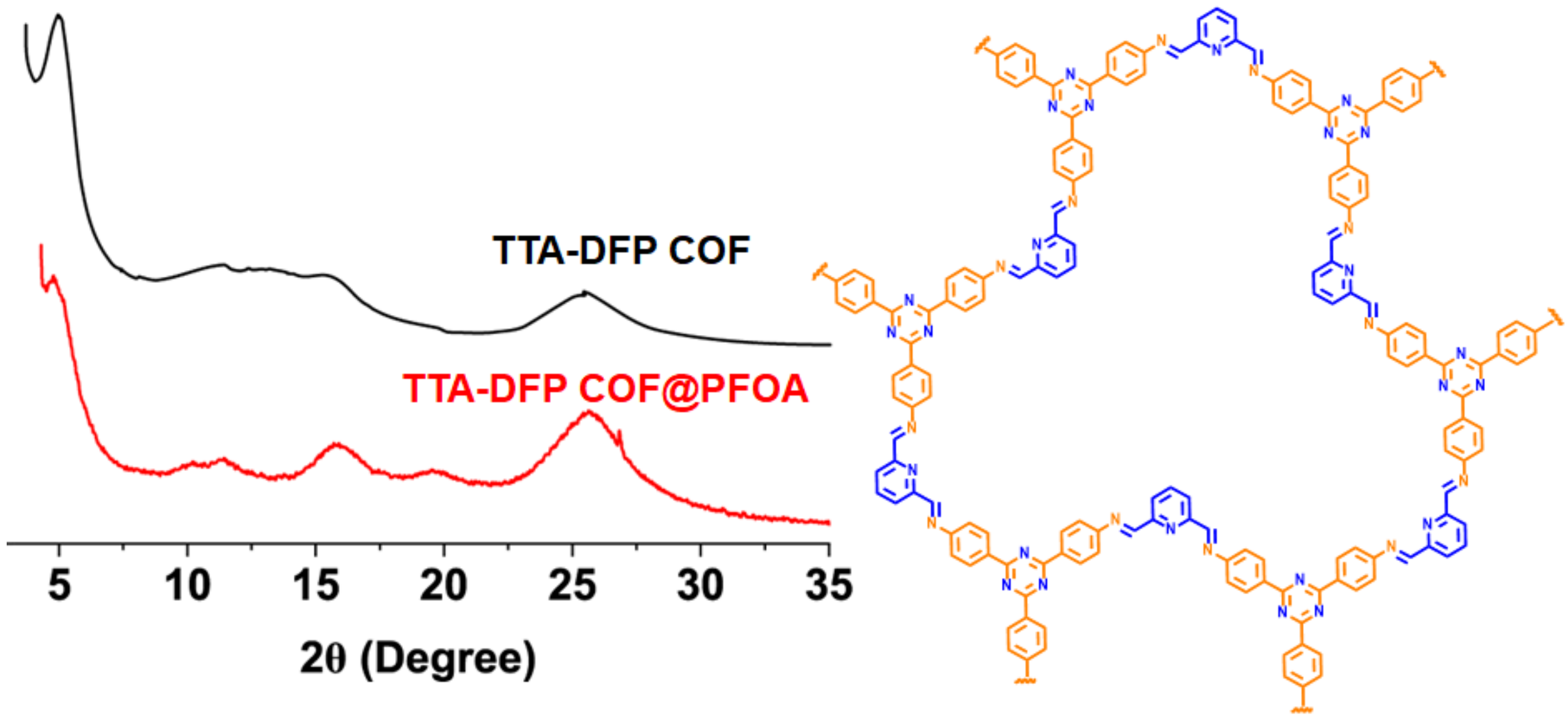

Figure S30. Powder X-ray diffraction (PXRD) patterns of TTA-DFP COF and TTA-DFP COF@PFOA. The inset shows the chemical structure of the pyrene-free TTA-DFP COF used as a control framework. In contrast to PY-TTA COF, exposure to PFOA produces only minor changes in the PXRD profile of TTA-DFP COF, indicating that the pronounced guest-responsive structural behavior observed for PY-TTA COF is closely associated with the presence of the pyrene-functionalized pore environment.

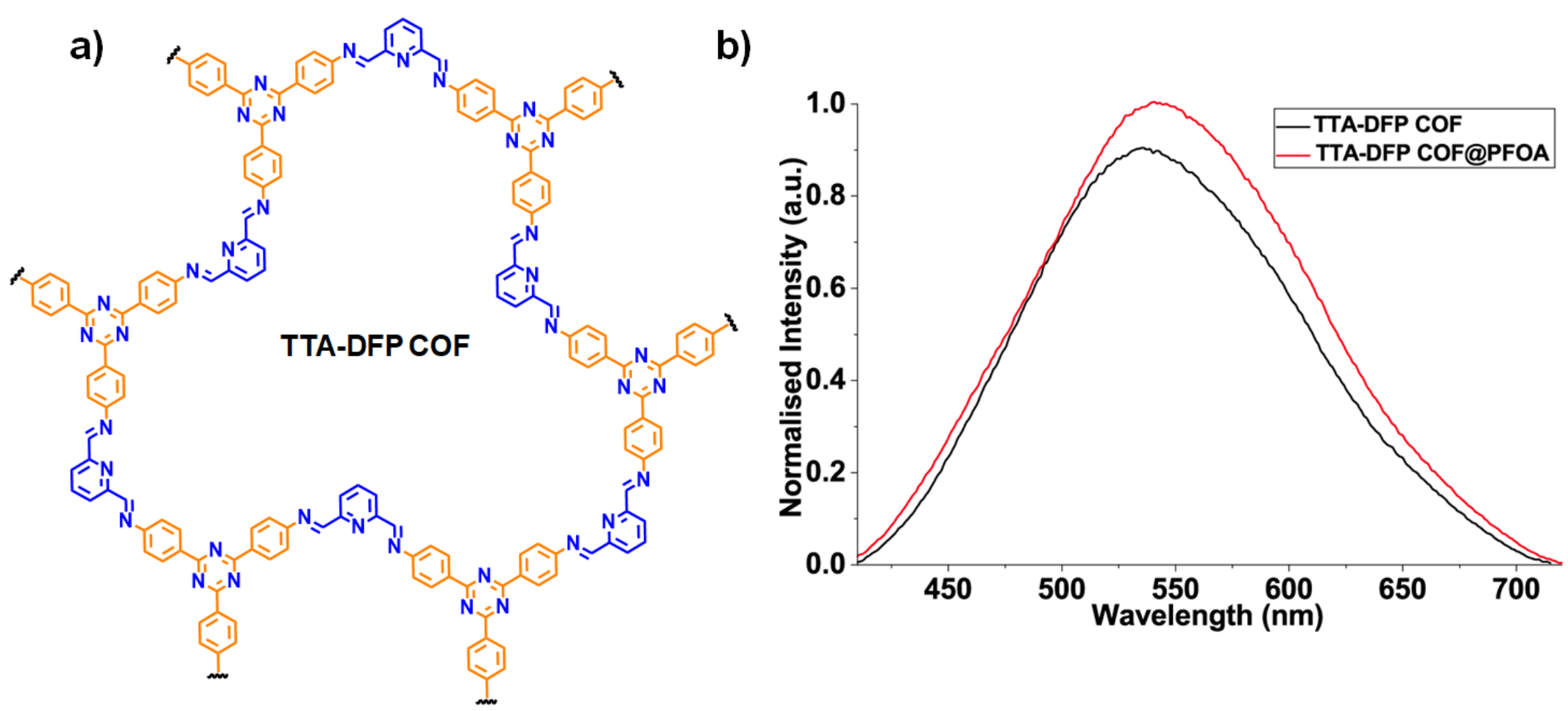

Figure S31. (a) Chemical structure of the pyrene-free analogue TTA-DFP COF used as a control framework. (b) Fluorescence emission spectra of TTA-DFP COF before and after PFOA treatment. In contrast to PY-TTA COF, the pyrene-free TTA-DFP COF exhibits only minor fluorescence changes upon exposure to PFOA, highlighting the critical role of the pyrene pendants in the guest-induced fluorescence response and molecular-locking mechanism.

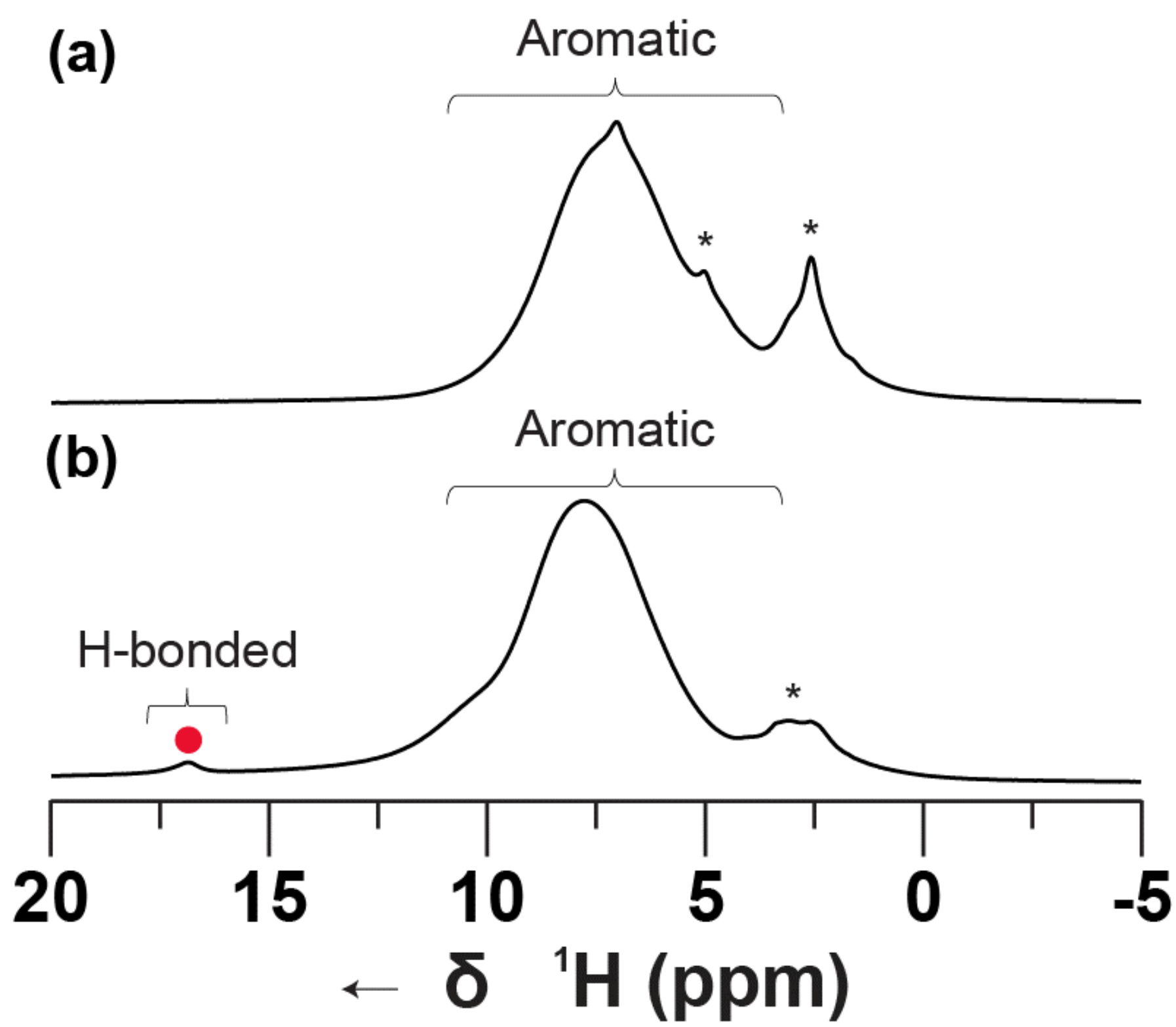

Figure S32. Fast MAS (40 kHz) ^{1}H solid-state NMR spectra of (a) pristine PY-TTA COF and (b) PY-TTA COF@PFOA. Asterisks (*) denote signals arising from adsorbed solvent molecules.

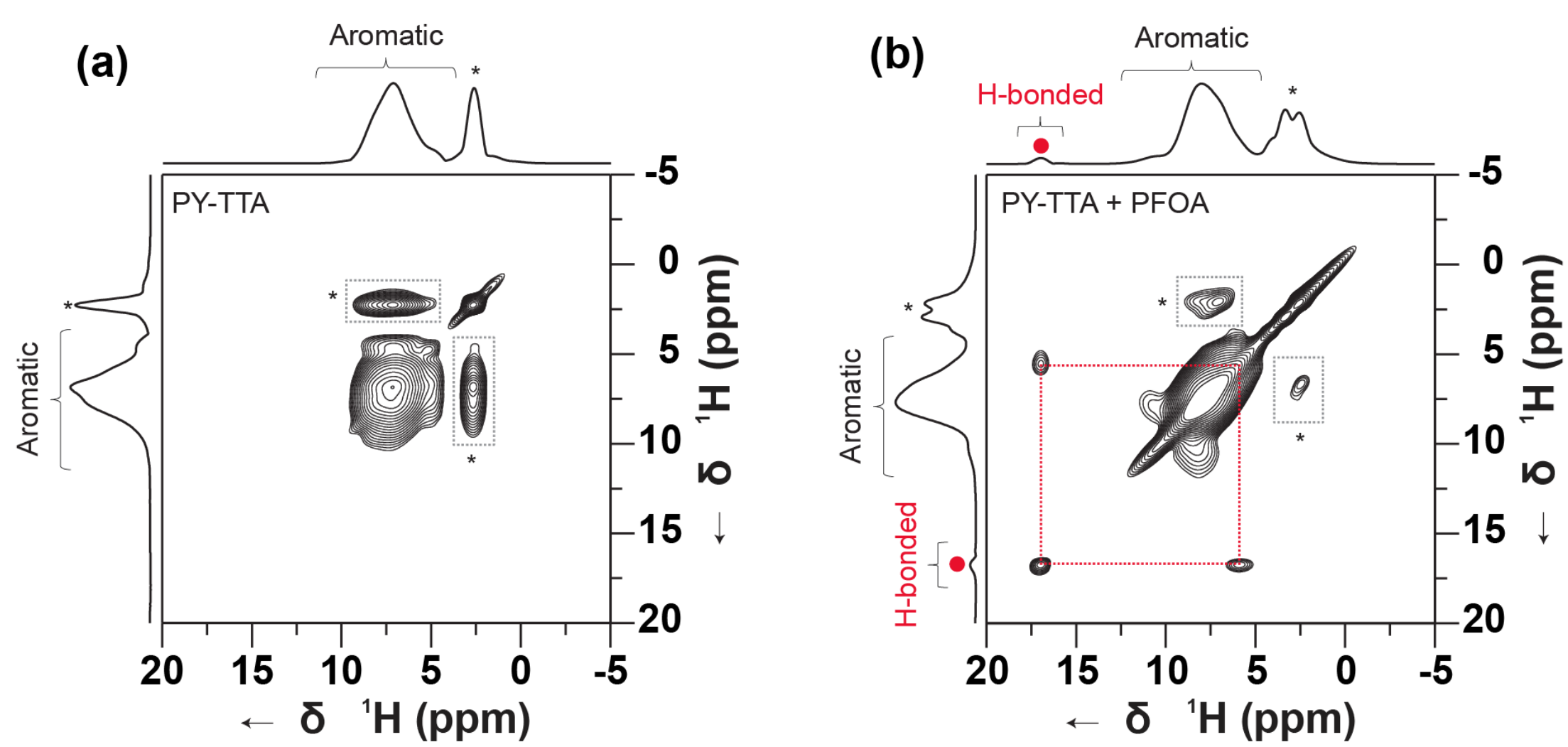

Figure S33. Fast MAS (40 kHz) ^{1}H–^{1}H NOESY-type solid-state NMR spectra of (a) pristine PY-TTA COF and (b) PY-TTA COF@PFOA. Asterisks (*) denote signals arising from adsorbed solvent molecules.

Table S1. Comparison of representative fluorescence-based PFOA sensors reported in the literature.

No	Sensing Materials	Analyte	Medium	Detection Mode	LOD (nM)
1	Guanidinocalix[5]arene[13]	PFOA	MeOH	Fluorescence "Turn-off"	61.81
2	PDI-MOF[14]	PFOA	DMF	Fluorescence "Turn-on"	3.1
3	MOF In(tcpp)[15]	PFOA	Water	Fluorescence "Turn-off"	3.1
4	Zn- cage[16]	PFOA	MeOH	Fluorescence "Turn-off"	61.81
5	meso-Ester-BODIPY[17]	PFOA	Water	Fluorescence "Turn-on"	2.8
6	Polymer[18]	PFOA	Water	Fluorescence "Turn-on"	0.1932
7	PPE/ ^{F}PPE Polymer[19]	PFOA	Water	Fluorescence "Turn-off/on"	142.5
8	TG-PD COF[5]	PFOA	Water	Fluorescence "Turn-on"	4.3
9	AMI cavitand[20]	PFOA	Water	Fluorescence "Turn-on"	130
10	2,6-bis(3,5-diethyl-*1H*-pyrrol-2-yl)pyridine[21]	PFOA	Hexane/Water	Fluorescence "Turn-on"	0.6
11	MOF (Eu-TPDC-NH_2)[22]	PFOA	DMF	Fluorescence "Turn-on"	23.1
12	PY-TTA COF (This work)	PFOA	Water	Fluorescence "Turn-on"	6.6

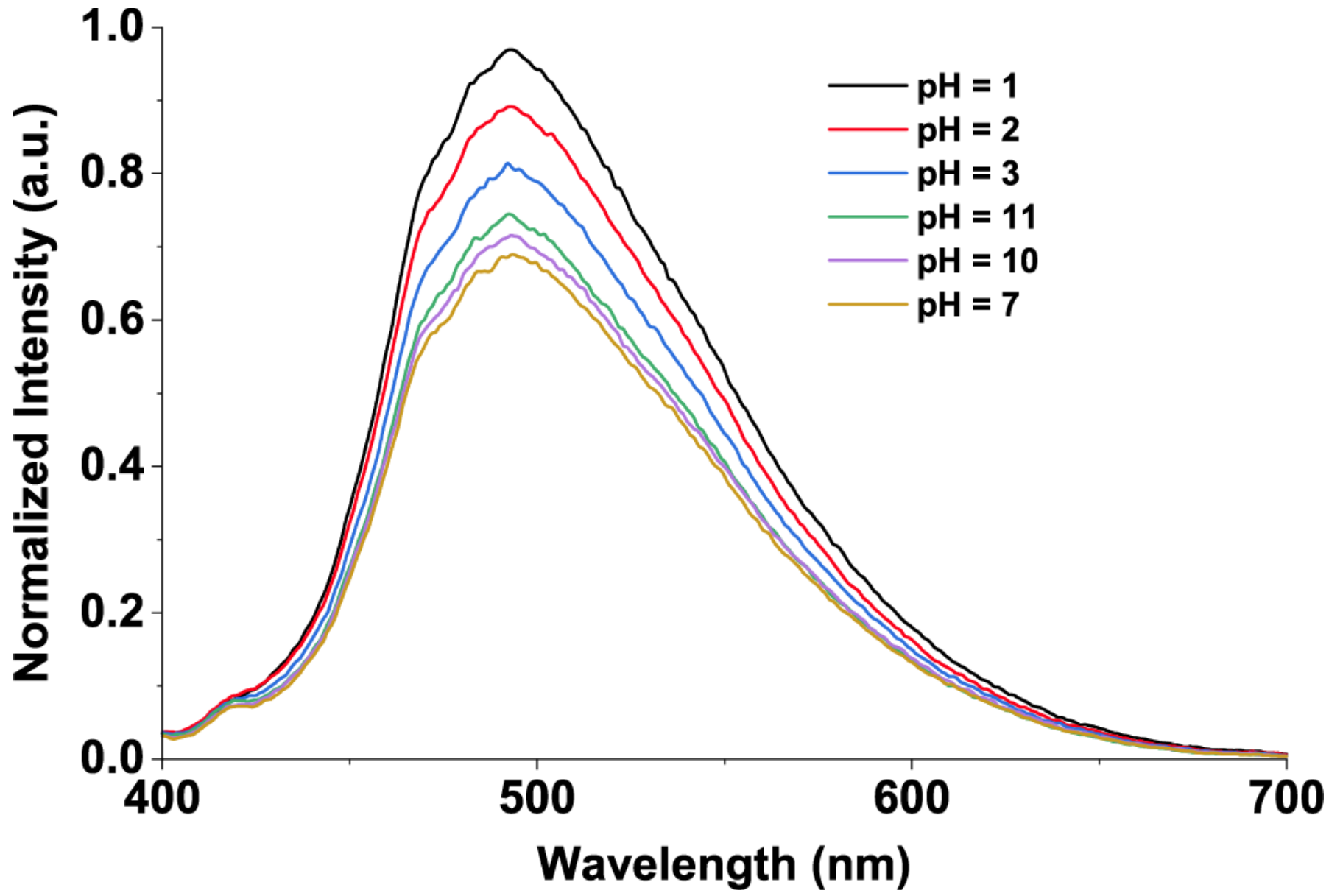

Figure S34. Fluorescence emission spectra of PY-TTA COF recorded at different pH values (pH 1–11). Although modest variations in fluorescence intensity are observed across the investigated pH range, the changes are substantially smaller than the fluorescence enhancement induced by PFOA binding.

16. Semiempirical and DFT calculations

Semiempirical and DFT calculations based on the GFN2-xTB and r^{2}SCAN-3c methods, respectively, were utilized in order to investigate the interactions of PFOA with the COF and identify the most stable adsorption sites. The GFN2-xTB method is chosen, as it has been shown to provide reasonable geometries and interaction energies for non-covalent complexes especially in the case of molecule binding in porous materials.[23, 24] Moreover, GFN2-xTB has been shown to efficiently identify the protonation sites in organic molecules.[25] Firstly, a molecular model of the COF was constructed. A 5-layer COF model based on the 2 x 2 x 5 supercell was constructed, with dangling bonds saturated with hydrogen atoms and its geometry was optimized. The COF model is shown in Figure S35. Subsequently, the most stable adsorption sites of a PFOA molecule in the COF model are identified by applying the automated Interaction Site Screening (aISS) of the docking submodule of xTB code.[26] Subsequently, the geometries of the most stable PFOA-COF conformers are minimized with the GFN2-xTB by applying strict criteria. This is done by considering the PFOA in its neutral and deprotonated form as well. Finally,

single point energy calculations are performed with the r^2SCAN-3c composite method combined with the SMD solvation model and considering water as solvent.[27, 28]

The results for the most stable adsorption configurations of both neutral and deprotonated PFOA are reported in Tables S2 and S3, and the geometries are shown in Figures S36 and S37. When considering the PFOA initially as neutral, a proton transfer is observed after the geometry optimizations from the PFOA towards the imine, triazine or pyridine groups of the COF, with the most stable protonation site being the imine group. The protonated pyridine and triazine are computed to be energetically higher by ~16 and ~43 kJ/mol respectively compared to the protonated imine. In some other cases, PFOA may retain the proton and form hydrogen-bonds with the pyridine groups of the COF. This case is energetically higher by ~22 kJ/mol compared to the deprotonated PFOA. The majority of the adsorption sites are stabilized mainly by hydrogen bonding and dispersion interactions. Dispersion interactions are observed between the aryl -C–H from the pyrene group of the COF and the $–CF_3$ groups of the PFOA. Hydrogen bonding may occur either between the carboxylate groups of the deprotonated PFOA with the protonated imine/triazine/pyridine, or between the proton of the neutral PFOA with the pyridine groups of the COF. These results are consistent with the findings from the solid-state NMR analysis.

When PFOA is considered as deprotonated, a weaker hydrogen bond between the oxygen atoms of the carboxylate moiety and the aryl or imine -C-H groups is observed. Additional stabilization of the complex is due to dispersion interactions between the $-CF_3$ and the aryl -CH groups of the COF.

Table S2. Lowest-energy adsorption configurations of PFOA within the pores of the PY-TTA COF model obtained using the r^{2}SCAN-3c/SMD(Water)/def2-mTZVPP method after geometry optimizations with the GFN2-xTB method. Relative energies (kJ mol^{-1}) are reported together with the dominant host–guest interactions, the protonation state of PFOA (neutral or deprotonated), and the corresponding protonation site within the framework. Relative energies are referenced to the most stable adsorption configuration.

**Conformation**	**GFN2-xTB Relative Energy (kJ mol^{-1})**	**r^{2}SCAN-3c/SMD(Water)/def2-mTZVPP Relative energies (kJ/mol)**	**PFOA Form**	**Primary Interaction(s)**	**Protonation Site**
1	0.0	7.1	Deprotonated	COO⁻···HN hydrogen bonding; C–F···π interactions	Imine
2	29.8	21.6	Neutral	Hydrogen bonding with pyridine	PFOA retained proton
3	31.6	42.2	Neutral	Hydrogen bonding with pyridine	PFOA retained proton
4	38.6	42.6	Deprotonated	COO⁻···HN hydrogen bonding; C–F···π interactions	Triazine
5	50.9	0.0	Deprotonated	COO⁻···HN hydrogen bonding; C–F···π interactions	Imine
6	53.4	28.5	Deprotonated	COO⁻···HN hydrogen bonding; C–F···π interactions	Imine
7	55.4	24.9	Neutral	Hydrogen bonding with imine; C–F···π interactions	PFOA retained proton
8	56.1	38.6	Deprotonated	COO⁻···HN hydrogen bonding; C–F···π interactions	Triazine
9	57.0	17.0	Deprotonated	COO⁻···HN hydrogen bonding; C–F···π interactions	Pyridine
10	57.5	16.3	Deprotonated	COO⁻···HN hydrogen bonding; C–F···π interactions	Pyridine

Table S3. Lowest-energy adsorption configurations of deprotonated PFOA within the pores of the PY-TTA COF model obtained using r^{2}SCAN-3c/SMD(Water)/def2-mTZVPP method after geometry optimizations with the GFN2-xTB method. Relative energies (kJ mol^{-1}) are reported together with the dominant host–guest interactions. Relative energies are referenced to the most stable adsorption configuration.

Conformation #N	GFN2-xTB Relative Energy (kJ mol^{-1})	r^{2}SCAN-3c/SMD(Water)/def2-mTZVPP Relative energies (kJ/mol)	Dominant Host–Guest Interaction(s)
1	0.0	0.0	COO$^-$···HC hydrogen bonding; C–F···π interactions
2	3.3	22.1	COO$^-$···HC hydrogen bonding; C–F···π interactions
3	18.1	26.0	COO$^-$···HC hydrogen bonding; C–F···π interactions
4	38.9	30.9	COO$^-$···HC hydrogen bonding; C–F···π interactions

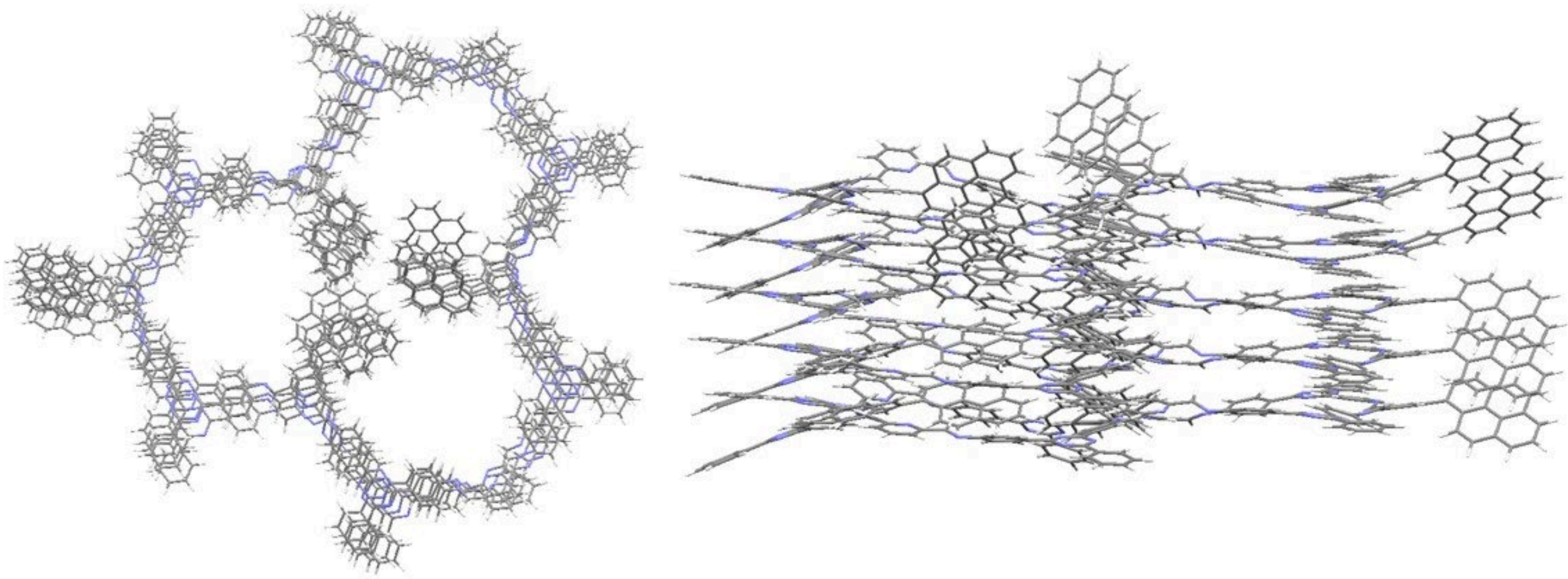

Figure S35. Structural model of PY-TTA COF used in the semiempirical GFN2-xTB calculations. Top and side views of the COF fragment employed to investigate PFOA adsorption, host–guest interactions, and the relative stability of the optimized binding configurations.

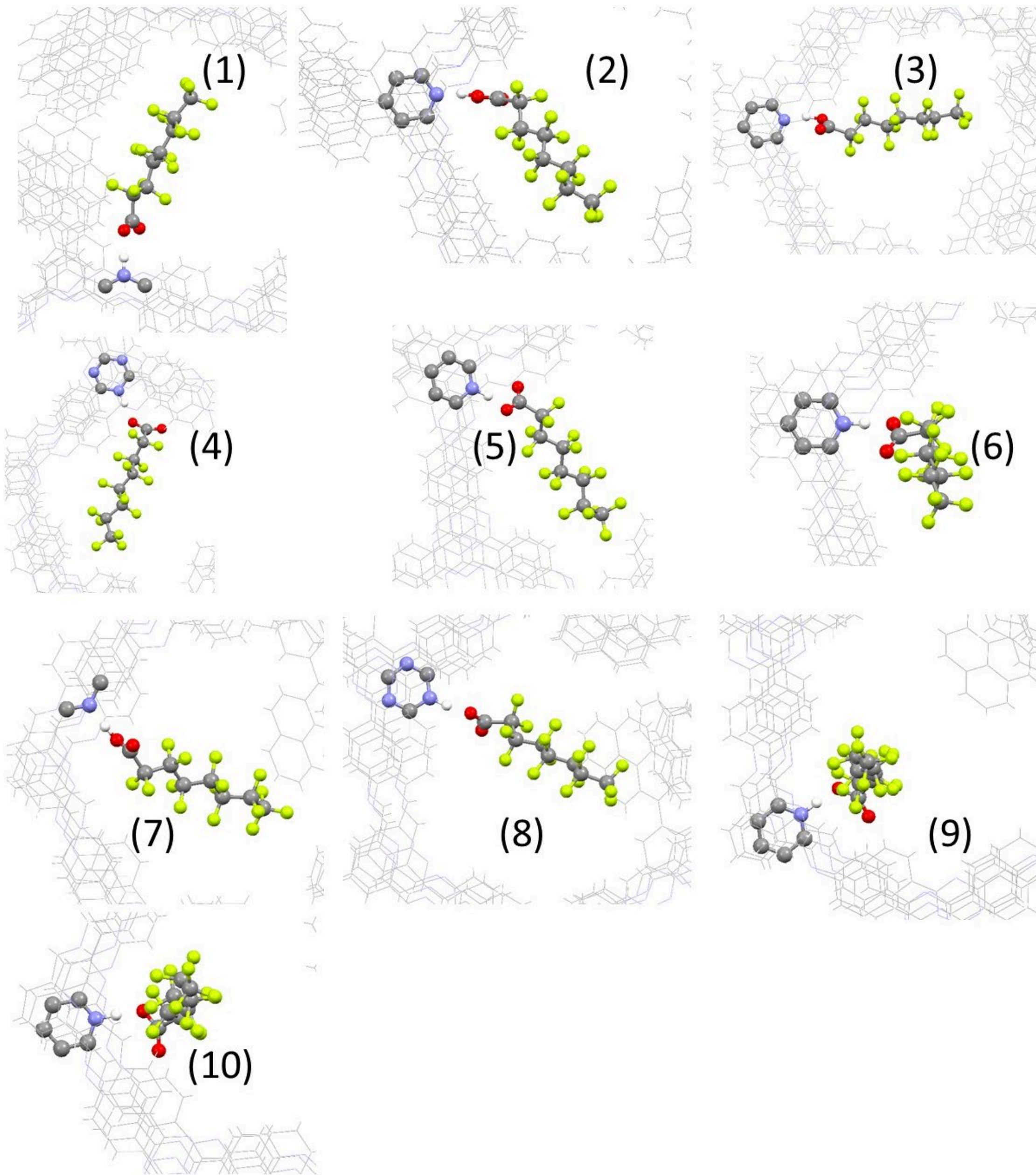

Figure S36. Lowest-energy adsorption configurations obtained by initially considering neutral PFOA within the pores of the PY-TTA COF model using GFN2-xTB calculations. Configurations (1–10) correspond to the structures listed in Table S2 and illustrate the diversity of favorable host–guest arrangements involving hydrogen-bonding interactions between PFOA and framework nitrogen sites, together with C–F···π interactions between the fluorinated tail and the aromatic pore environment.

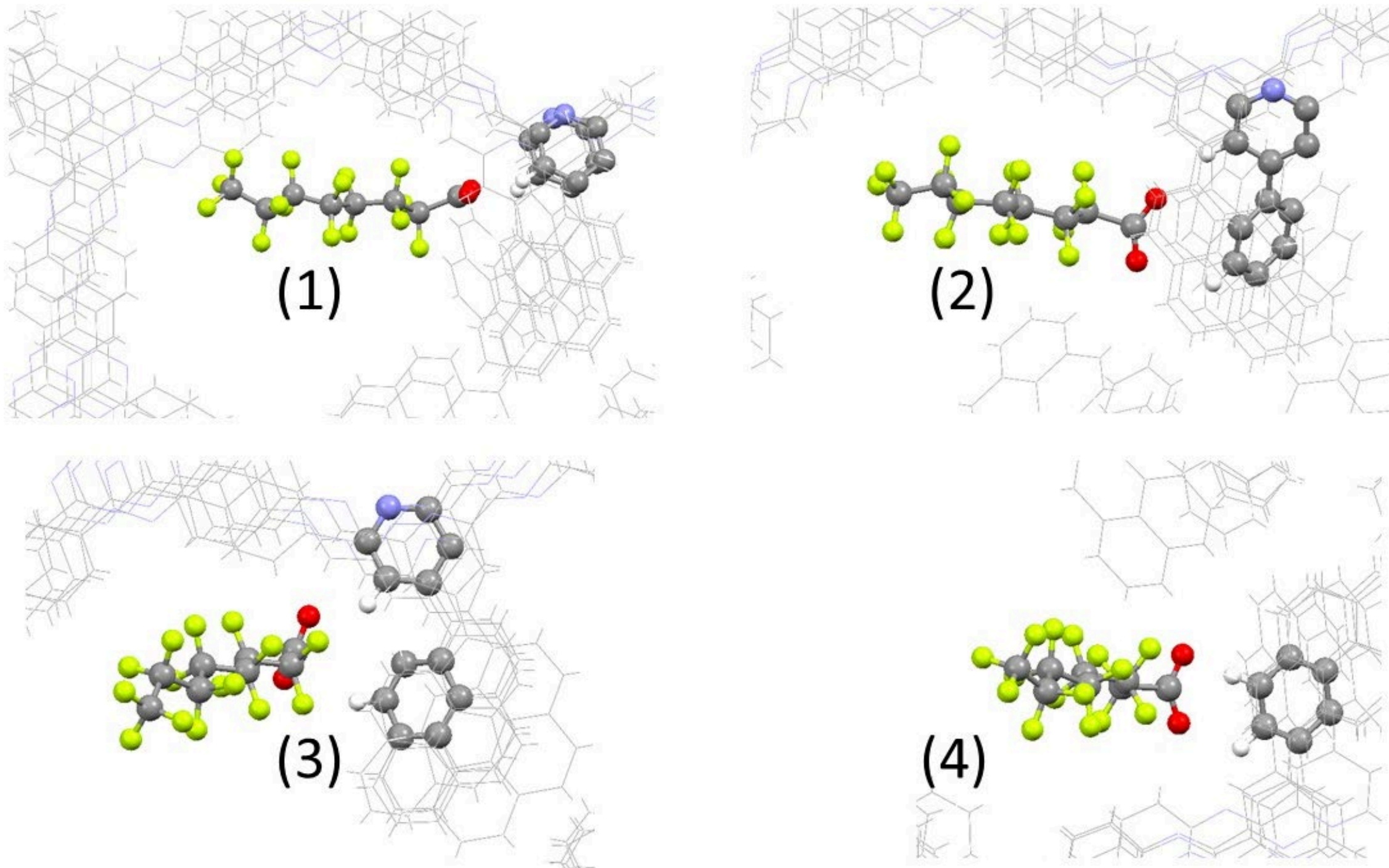

Figure S37. Lowest-energy adsorption configurations of deprotonated PFOA within the pores of the PY-TTA COF model obtained from GFN2-xTB calculations. Configurations (1–4) correspond to the structures summarized in Table S3. The optimized geometries reveal adsorption modes stabilized by $COO^{-}\cdots HC$ interactions and C–F⋯π contacts between the fluorinated tail of PFOA and the aromatic surfaces of the framework.

17. References

(1) Price, D. J.; Brooks III, C. L. A modified TIP3P water potential for simulation with Ewald summation. *The Journal of chemical physics* **2004**, *121* (20), 10096-10103.
(2) Mayo, S. L.; Olafson, B. D.; Goddard, W. A. DREIDING: a generic force field for molecular simulations. *Journal of Physical chemistry* **1990**, *94* (26), 8897-8909.
(3) Garai, B.; Shetty, D.; Skorjanc, T.; Gándara, F.; Naleem, N.; Varghese, S.; Sharma, S. K.; Baias, M.; Jagannathan, R.; Olson, M. A. Taming the topology of calix [4] arene-based 2D-covalent organic frameworks: interpenetrated vs noninterpenetrated frameworks and their selective removal of cationic dyes. *Journal of the American Chemical Society* **2021**, *143* (9), 3407-3415.
(4) Skorjanc, T.; Shetty, D.; Gándara, F.; Pascal, S.; Naleem, N.; Abubakar, S.; Ali, L.; Mohammed, A. K.; Raya, J.; Kirmizialtin, S. Covalent organic framework based on azacalix [4] arene for the efficient capture of dialysis waste products. *ACS applied materials & interfaces* **2022**, *14* (34), 39293-39298.
(5) Jrad, A.; Das, G.; Alkhatib, N.; Prakasam, T.; Benyettou, F.; Varghese, S.; Gándara, F.; Olson, M.; Kirmizialtin, S.; Trabolsi, A. Cationic covalent organic framework for the fluorescent sensing and cooperative adsorption of perfluorooctanoic acid. *Nature Communications* **2024**, *15* (1), 10490.
(6) Abraham, M. J.; Murtola, T.; Schulz, R.; Páll, S.; Smith, J. C.; Hess, B.; Lindahl, E. GROMACS: High performance molecular simulations through multi-level parallelism from laptops to supercomputers. *SoftwareX* **2015**, *1*, 19-25.
(7) McSherry, T. M. A general steepest descent algorithm. *IEEE Transactions on Aerospace and Electronic Systems* **1976**, (1), 12-22.
(8) Van Gunsteren, W. F.; Berendsen, H. J. A leap-frog algorithm for stochastic dynamics. *Molecular Simulation* **1988**, *1* (3), 173-185.
(9) Darden, T.; York, D.; Pedersen, L. Particle mesh Ewald: An N log (N) method for Ewald sums in large systems. *Journal of chemical physics* **1993**, *98* (12), 10089-10092.
(10) Frisch, M.; Trucks, G.; Schlegel, H.; Scuseria, G.; Robb, M.; Cheeseman, J.; Scalmani, G.; Barone, V.; Petersson, G.; Nakatsuji, H. Gaussian 16 Revision C. 01. 2016; GaussianInc. *Wallingford CT* **2016**, *421*.
(11) Halder, A.; Kandambeth, S.; Biswal, B. P.; Kaur, G.; Roy, N. C.; Addicoat, M.; Salunke, J. K.; Banerjee, S.; Vanka, K.; Heine, T.; et al. Decoding the Morphological Diversity in Two Dimensional Crystalline Porous Polymers by Core Planarity Modulation. *Angewandte Chemie International Edition* **2016**, *55* (27), 7806-7810. DOI: https://doi.org/10.1002/anie.201600087.
(12) Prakasam, T.; Sharma, S. K.; Ravaux, F.; Benyettou, F.; Lusi, M.; Sabu, V.; Bazin, P.; Delclos, T.; Jagannathan, R.; Whelan, J.; et al. 2D covalent organic framework via catenation. *Chem* **2025**, *11* (2). DOI: 10.1016/j.chempr.2024.09.006 (accessed 2025/02/20).
(13) Zheng, Z.; Yu, H.; Geng, W.-C.; Hu, X.-Y.; Wang, Y.-Y.; Li, Z.; Wang, Y.; Guo, D.-S. Guanidinocalix[5]arene for sensitive fluorescence detection and magnetic removal of perfluorinated pollutants. *Nature Communications* **2019**, *10* (1), 5762. DOI: 10.1038/s41467-019-13775-1.
(14) Dalapati, R.; Hunter, M.; Sk, M.; Yang, X.; Zang, L. Fluorescence Turn-on Detection of Perfluorooctanoic Acid (PFOA) by Perylene Diimide-Based Metal–Organic Framework. *ACS Applied Materials & Interfaces* **2024**, *16* (25), 32344-32356. DOI: 10.1021/acsami.4c03389.
(15) Yin, H.-Q.; Tan, K.; Jensen, S.; Teat, S. J.; Ullah, S.; Hei, X.; Velasco, E.; Oyekan, K.; Meyer, N.; Wang, X.-Y.; et al. A switchable sensor and scavenger: detection and removal of fluorinated chemical species by a luminescent metal–organic framework. *Chemical Science* **2021**, *12* (42), 14189-14197, 10.1039/D1SC04070G. DOI: 10.1039/D1SC04070G.

(16) He, Y.; Luo, D.; Lynch, V. M.; Ahmed, M.; Sessler, J. L.; Chi, X. Porous adaptive luminescent metallacage for the detection and removal of perfluoroalkyl carboxylic acids. *Chem* **2023**, *9* (1), 93-101. DOI: 10.1016/j.chempr.2022.09.004 (accessed 2026/06/10).
(17) Cho, S.; Kim, Y. J-Aggregate-Triggering BODIPYs: an Ultrasensitive Chromogenic and Fluorogenic Sensing Platform for Perfluorooctanesulfonate. *Chemistry–A European Journal* **2023**, *29* (71), e202302897.
(18) Concellón, A.; Swager, T. M. Detection of per-and polyfluoroalkyl substances (PFAS) by interrupted energy transfer. *Angewandte Chemie* **2023**, *135* (47), e202309928.
(19) Concellón, A.; Castro-Esteban, J.; Swager, T. M. Ultratrace PFAS detection using amplifying fluorescent polymers. *Journal of the American Chemical Society* **2023**, *145* (20), 11420-11430.
(20) Lian, R.; Yang, Y.-D.; Moreno, J. L., Jr.; Eggimann, B. L.; Chen, J.; Gibson-Elias, L. J.; Williams, C. G.; Jiang, P.; Lee, A. J.; Mueller, L. J.; et al. Self-Aggregative Recognition and Extraction of Perfluorooctanesulfonate with Flexible Cationic Water-Soluble Deep Cavitands. *Journal of the American Chemical Society* **2025**, *147* (26), 22768-22777. DOI: 10.1021/jacs.5c04762.
(21) Yang, Y. D.; Zhang, Q.; Jin, X.; Chau, C. V.; Yang, J.; Henkelman, G.; Chi, X.; Gong, H. Y.; Hooley, R. J.; Sessler, J. L. Readily visualized perfluorooctanoic acid detection using a small molecule chemosensor. *Angewandte Chemie International Edition* **2025**, *64* (19), e202501245.
(22) Su, L.; Niu, Q.; Liu, J.; Liu, X.; Wang, Y.; Ge, Z.; Ye, Y.; Li, Z. Ratiometric luminescence detection of PFOA by amine-functionalized lanthanide metal-organic-frameworks. *Chemical Engineering Journal* **2025**, *520*, 166124. DOI: https://doi.org/10.1016/j.cej.2025.166124.
(23) Bannwarth, C.; Caldeweyher, E.; Ehlert, S.; Hansen, A.; Pracht, P.; Seibert, J.; Spicher, S.; Grimme, S. Extended tight-binding quantum chemistry methods. *WIREs Computational Molecular Science* **2021**, *11* (2), e1493. DOI: https://doi.org/10.1002/wcms.1493.
(24) Spicher, S.; Bursch, M.; Grimme, S. Efficient Calculation of Small Molecule Binding in Metal–Organic Frameworks and Porous Organic Cages. *The Journal of Physical Chemistry C* **2020**, *124* (50), 27529-27541. DOI: 10.1021/acs.jpcc.0c08617.
(25) Pracht, P.; Bauer, C. A.; Grimme, S. Automated and efficient quantum chemical determination and energetic ranking of molecular protonation sites. *Journal of Computational Chemistry* **2017**, *38* (30), 2618-2631. DOI: https://doi.org/10.1002/jcc.24922.
(26) Plett, C.; Grimme, S. Automated and Efficient Generation of General Molecular Aggregate Structures. *Angewandte Chemie International Edition* **2023**, *62* (4), e202214477. DOI: https://doi.org/10.1002/anie.202214477.
(27) Grimme, S.; Hansen, A.; Ehlert, S.; Mewes, J.-M. r^2SCAN-3c: A “Swiss army knife” composite electronic-structure method. *The Journal of Chemical Physics* **2021**, *154* (6). DOI: 10.1063/5.0040021 (accessed 6/25/2026).
(28) Marenich, A. V.; Cramer, C. J.; Truhlar, D. G. Universal Solvation Model Based on Solute Electron Density and on a Continuum Model of the Solvent Defined by the Bulk Dielectric Constant and Atomic Surface Tensions. *The Journal of Physical Chemistry B* **2009**, *113* (18), 6378-6396. DOI: 10.1021/jp810292n.
